# Clinical Performance and Communication Skills of ChatGPT Versus Physicians in Emergency Medicine: Simulated Patient Study

**DOI:** 10.2196/68409

**Published:** 2025-07-17

**Authors:** ChulHyoung Park, Min Ho An, Gyubeom Hwang, Rae Woong Park, Juho An

**Affiliations:** 1 Department of Biomedical Informatics, Ajou University School of Medicine, Suwon, Republic of Korea; 2 Center for Biomedical Informatics Research, Ajou University Medical Cencer, Suown, Republic of Korea; 3 Department of Medical Sciences, Graduate School of Ajou University, Suwon, Republic of Korea; 4 BK21 R&E Initiative for Advanced Precision Medicine, Suwon, Republic of Korea; 5 Department of Emergency Medicine, Ajou University School of Medicine, 164 Worldcup-ro, Yeongtong-gu, Suwon, 16499, Republic of Korea, 82 0312195016

**Keywords:** artificial intelligence, large language model, ChatGPT, emergency medicine, clinical performance examination, history taking, clinical reasoning, empathy, patient experience

## Abstract

**Background:**

Emergency medicine can benefit from artificial intelligence (AI) due to its unique challenges, such as high patient volume and the need for urgent interventions. However, it remains difficult to assess the applicability of AI systems to real-world emergency medicine practice, which requires not only medical knowledge but also adaptable problem-solving and effective communication skills.

**Objective:**

We aimed to evaluate ChatGPT’s (OpenAI) performance in comparison to human doctors in simulated emergency medicine settings, using the framework of clinical performance examination and written examinations.

**Methods:**

In total, 12 human doctors were recruited to represent the medical professionals. Both ChatGPT and the human doctors were instructed to manage each case like real clinical settings with 12 simulated patients. After the clinical performance examination sessions, the conversation records were evaluated by an emergency medicine professor on history taking, clinical accuracy, and empathy on a 5-point Likert scale. Simulated patients completed a 5-point scale survey including overall comprehensibility, credibility, and concern reduction for each case. In addition, they evaluated whether the doctor they interacted with was similar to a human doctor. An additional evaluation was performed using vignette-based written examinations to assess diagnosis, investigation, and treatment planning. The mean scores from ChatGPT were then compared with those of the human doctors.

**Results:**

ChatGPT scored significantly higher than the physicians in both history-taking (mean score 3.91, SD 0.67 vs mean score 2.67, SD 0.78, *P*<.001) and empathy (mean score 4.50, SD 0.67 vs mean score 1.75, SD 0.62, *P*<.001). However, there was no significant difference in clinical accuracy. In the survey conducted with simulated patients, ChatGPT scored higher for concern reduction (mean score 4.33, SD 0.78 vs mean score 3.58, SD 0.90, *P*=.04). For comprehensibility and credibility, ChatGPT showed better performance, but the difference was not significant. In the similarity assessment score, no significant difference was observed (mean score 3.50, SD 1.78 vs mean score 3.25, SD 1.86, *P*=.71).

**Conclusions:**

ChatGPT’s performance highlights its potential as a valuable adjunct in emergency medicine, demonstrating comparable proficiency in knowledge application, efficiency, and empathetic patient interaction. These results suggest that a collaborative health care model, integrating AI with human expertise, could enhance patient care and outcomes.

## Introduction

Recent advancements in artificial intelligence (AI) have raised interest in its potential to complement or even replace human expertise across various fields, particularly in medicine [1]. AI systems, such as ChatGPT (OpenAI), have demonstrated notable clinical capabilities, including success in professional medical examinations like the United States Medical Licensing Examination [2]. In a study by Sarraju et al [3], conversational generative AI models provided appropriate responses to 84% of questions on cardiovascular disease prevention, demonstrating their capability to handle open-ended clinical prompts rather than multiple-choice questions. In subsequent studies, ChatGPT has shown promise in more complex tasks, providing knowledge and management recommendations for hepatic cirrhosis and hepatocellular carcinoma [4]. Furthermore, Ayers et al [5] reported that ChatGPT generated empathetic responses to patient inquiries on a web-based platform comparable to those of human physicians, underscoring its potential to engage effectively with patients.

Emergency medicine is characterized by an urgent need for rapid diagnosis and immediate treatment. The high patient load and strict time constraints in emergency departments increase the risk of essential clinical information being overlooked or misinterpreted, including symptom presentation and diagnostic test results [6,7]. In addition, extended shifts, often spanning 24 hours, contribute to clinician fatigue and the risk of human error. AI technologies offer promising solutions to these challenges by enabling rapid, fatigue-free data processing. These challenges have motivated initiatives to integrate AI into emergency settings, particularly to enhance diagnostic speed and accuracy in radiology [8]. However, the implementation of AI in emergency medicine remains constrained by technical and operational challenges [9-11]. Emergency practice demands not only technical proficiency but also the flexibility to adapt swiftly to evolving conditions and effective communication skills to engage with patients in high-stress situations. Recent advancements in large language models (LLMs) have introduced conversational interfaces that can adapt responsively to new information, potentially aligning well with the dynamic demands of emergency medicine [12]. However, there remains a lack of research on the application of AI in emergency medicine within environments that closely resemble real-world clinical settings, as many studies have been limited to assessing the ability of AI to solve static examination-based questions rather than clinical practice-based problems [13,14].

The clinical performance examination (CPX) is a form of testing that assesses problem-solving skills in real-world–like clinical situations with simulated patients. It was developed from the recognition that assessing a physician’s clinical skills cannot be fully achieved through traditional examination formats [15]. The CPX provides a dynamic approach to evaluating and refining essential competencies in simulated real-world settings. Through CPX, participants engage in history taking, communication, and patient relationship management and receive feedback for continuous improvement. Studies have shown that CPX enhances physicians’ diagnostic accuracy, communication skills, and adaptability in clinical interactions, all of which are crucial in the field of emergency medicine [16,17]. Furthermore, CPX allows for the assessment of nontechnical skills, such as empathy, which is difficult to evaluate through conventional examination-based scenarios [18]. This ability to observe both technical and interpersonal skills offers a more comprehensive approach, aligning closely with the complex demands of emergency medicine practice.

In this study, we aimed to evaluate ChatGPT within the context of emergency medicine using a framework of CPX combined with a written examination, comparing its performance to that of human doctors. Through this comparative analysis, we sought to provide a deeper understanding of how AI can effectively augment clinical practice, enhance decision-making processes, and support health care professionals.

## Methods

### Ethical Considerations

This cross-sectional study received ethical approval from the institutional review board of Ajou University Medical Center (approval AJOUIRB-OB-2024‐277). To maintain the confidentiality of participants, all identifying information has been appropriately anonymized or omitted, and informed consent (Multimedia Appendix 1) was obtained from all participants before their involvement in the study. All research methods were conducted in accordance with the Declaration of Helsinki. The study did not involve any form of participant remuneration.

### Study Design

**Figure 1.**
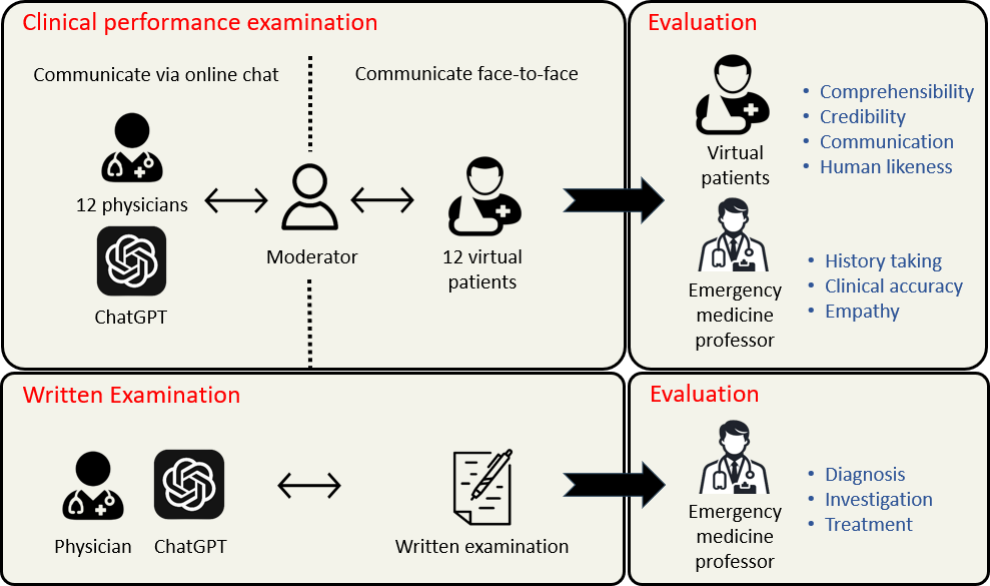
Flow diagram of the study.

The overall study flow is illustrated in Figure 1. This study aimed to compare the performance of ChatGPT and human doctors in simulated emergency medicine scenarios using 2 approaches—CPXs and conventional written examinations.

In the CPX, simulated patients represented standardized clinical cases. ChatGPT and human doctors participated in patient consultations via a neutral moderator to ensure blind evaluation. After each consultation, the emergency medicine professor evaluated the consultation from full conversation scripts. Simulated patients independently rated their consultation experiences.

In the written examination, ChatGPT and human doctors were provided with clinical vignettes describing chief complaints and relevant medical history. They were instructed to identify the most likely diagnosis, suggest appropriate diagnostic investigations, and recommend a suitable treatment plan. The answers were evaluated by an emergency medicine professor.

The primary objective of this study was to compare the clinical competency of ChatGPT and human doctors in the emergency medicine clinical settings in CPX and written examination formats. The secondary objective was to compare patient-reported experience, as evaluated by simulated patients during the CPXs.

### Case Selection

For the CPX, 4 representative cases were selected from 100 Cases in Emergency Medicine and Critical Care by Shamil et al [19]. The selected cases—malaria, acute asthma exacerbation, metastatic spinal cord compression, and acute kidney injury—were chosen based on their clinical urgency and the need for comprehensive symptom assessment and differential diagnosis. In addition, 28 text-based scenarios and 4 image-based clinical vignettes, along with corresponding questions, were selected for the written examinations. The selection was designed to include diverse representations across 16 different specialties, such as cardiology, pediatrics, and obstetrics, with a focus on scenarios commonly encountered in emergency care. For cases involving imaging, an electrocardiogram demonstrating atrial fibrillation, a brain computed tomography scan indicating subarachnoid hemorrhage, a chest x-ray showing a rib fracture, and a knee x-ray of a patient presenting with a knee injury were included in the case presentations. Detailed information about the cases that used written examinations in the study, including each patient’s chief complaint and diagnosis, is presented in Table 1.

**Table 1.** Chief complaints, medical specialties, and diagnosis of cases included in the study.

Case number	Chief complaint	Medical specialty	Disease	Type^a^	Reference^b^
Case 1	Fever, headache, and a rash	Infectious medicine	Bacterial meningitis	1	5
Case 2	Dysuria and weakness	Infectious medicine	Urinary tract infection	1	18
Case 3	Slurred speech and weakness	Neurology	Ischemic stroke	1	41
Case 4	Severe epigastric pain	General surgery	Perforated peptic ulcer	1	60
Case 5	Epigastric pain and nausea	Gastroenterology	Acute pancreatitis	1	64
Case 6	A child acutely short of breath	Pediatrics	Severe asthma exacerbation	1	83
Case 7	Headache in pregnancy	Obstetrics and gynecology	Pre-eclampsia	1	95
Case 8	Shortness of breath and painful swallowing	Otolaryngology	Supraglottitis	1	3
Case 9	Nausea and vomiting in a diabetic	Endocrinology	Diabetic ketoacidosis	1	6
Case 10	Feeling unwell while on chemotherapy	Oncology and hematology	Neutropenic sepsis	1	24
Case 11	Cat bite	Orthopedics surgery	Tenosynovitis	1	50
Case 12	Left iliac fossa pain with fever	Gastroenterology	Diverticulitis	1	61
Case 13	Abdominal pain in early pregnancy	Obstetrics and gynecology	Ectopic pregnancy	1	89
Case 14	Breathlessness in pregnancy	Obstetrics and gynecology	Pulmonary embolism	1	96
Case 15	A productive cough	Respiratory medicine	Acute exacerbation of chronic obstructive pulmonary disease	1	16
Case 16	Productive cough and shortness of breath	Respiratory medicine	Bacterial pneumonia	1	25
Case 17	Acute severe leg pain	Cardiology	Acute ischemia	1	62
Case 18	Right flank pain moving to the groin	Urology	Kidney stone	1	66
Case 19	Worsening ear pain	Otolaryngology	Acute otitis media	1	69
Case 20	Cough and difficulty breathing in an infant	Pediatrics	Bronchiolitis	1	78
Case 21	Pelvic pain	Obstetrics and gynecology	Ovarian cyst torsion	1	91
Case 22	A collapse at work	Cardiology	Massive pulmonary embolism	1	17
Case 23	Upper abdominal pain	General surgery	Acute cholecystitis	1	57
Case 24	Abdominal pain and nausea	General surgery	Acute appendicitis	1	63
Case 25	Ear pain with discharge and facial weakness	Otolaryngology	Malignant otitis media	1	71
Case 26	A swollen eyelid	Ophthalmology	Periorbital preseptal cellulitis	1	73
Case 27	My son has the “runs”	Pediatrics	Gastroenteritis	1	81
Case 28	Abdominal pain and vaginal discharge	Obstetrics and gynecology	Pelvic inflammatory disease	1	92
Case 29	An abnormal electrocardiogram	Cardiology	Atrial fibrillation	2	30
Case 30	Headache, vomiting, and confusion	Neurosurgery	Subarachnoid hemorrhage	2	39
Case 31	Twisted my knee skiing	Orthopedic surgery	Rupture of the anterior cruciate ligament	2	53
Case 32	My ribs hurt	Cardiothoracic surgery	Traumatic pneumothorax	2	59

a The written examination consisted of two types of questions: (1) text-based questions, and (2) image-included questions.

b The questions were developed based on emergency medicine patient cases from the reference “100 Cases in Emergency Medicine and Critical Care” by Shamil et al [19]. The numbers provided correspond to those accessible in the reference.

### Human Doctors and ChatGPT in CPX

In total, 12 human doctors were recruited to represent the medical doctors, comprising 4 third-year emergency medicine residents, 4 fourth-year emergency medicine residents, and 4 general practitioners (GPs) from a tertiary hospital in South Korea. Before participating in the CPX with simulated patients, physicians received general instructions for the examination process (Table S1 in Multimedia Appendix 2). They were informed that all conversations with simulated patients would be conducted via text-based communication through a moderator. They were directed to approach each case as if practicing in a real clinical setting, with the understanding that the information necessary for diagnosis and treatment planning could be obtained by asking questions to the simulated patients.

ChatGPT (version 4.0) was used in this study, with all examinations conducted between September and October 2023. For ChatGPT, a prompt designed to emulate the role of a medical professional was provided (Table S2 in Multimedia Appendix 2). ChatGPT was instructed to perform history taking and, when a physical examination was required, to obtain relevant information through targeted questions. In addition, it was directed to communicate possible diagnoses to the patient and to provide explanations regarding necessary tests and treatment plans.

### Interaction With Simulated Patients in CPX

In total, 12 volunteers were recruited as simulated patients and evenly distributed across 4 clinical scenarios, with 3 simulated patients assigned to each scenario. Each simulated patient participated in 2 separate consultations, presenting their assigned case to both ChatGPT and a human doctor. Depending on the allocation, each simulated patient conducted 1 consultation with ChatGPT and 1 with either a third-year emergency medicine resident (R3), a fourth-year resident (R4), or a GP.

Before the CPX, each patient reviewed their assigned case and received instructions on their role (Table S3 in Multimedia Appendix 2). They were informed that all responses would be delivered through a moderator and that they would participate in 2 consultations, 1 with a human doctor and 1 with ChatGPT. They were also notified that their experience would be evaluated afterward.

During the CPX, patients did not interact face-to-face with either ChatGPT or the physicians; instead, they communicated with assistance from a moderator. All questions from doctors or ChatGPT were provided to patients as text by the moderator, and patients’ responses were similarly relayed in text form by the moderator to maintain consistency. This indirect communication process ensured that patients were blinded to whether they were interacting with ChatGPT or a human doctor. To standardize message formats, doctors’ messages were edited to remove typos and to consolidate brief questions where appropriate (eg, “Do you take any other medications? Like for a cold?” was revised to “Are you taking any other medications, such as cold medicine?”). Although the protocol advised posing one question at a time, some questions were grouped by physicians and later separated by the moderator for consistency. Given ChatGPT’s quicker response capability, its message delivery was deliberately slowed to mirror a doctor’s pace. No additional modifications were made to ChatGPT’s messages.

### Written Examinations

The written examination consisted of 28 text-based clinical cases and 4 image-containing clinical cases. Before the examination, both physicians and ChatGPT were provided with written instructions outlining the format of the test. These included a sample case, sample question and answer, and detailed scoring criteria to ensure consistency in understanding and response approach. The full instructions are presented in Table S3 in Multimedia Appendix 2.

A total of 4 GPs, 4 third-year emergency medicine residents (R3s), and 4 fourth-year residents (R4s) each completed 7 cases, resulting in 28 responses for text-based clinical cases per group. ChatGPT also provided answers to all 28 cases. In addition, 4 image-based clinical cases were evaluated. Due to concerns regarding potential interpretive inaccuracies, GPs did not participate in the image-based clinical cases. Each of the 4 R3s and 4 R4s completed all 4 cases, yielding 32 responses for image-based cases. ChatGPT also completed all 4 image-based cases.

Detailed instructions, including sample questions and grading criteria, were provided to both ChatGPT and the doctors. (Table S4 in Multimedia Appendix 2). The grading criteria encompassed the number of correct keywords, the specificity of responses, and the presence of any inappropriate content.

### Assessment of Outcomes

The conversations were evaluated by a professor of emergency medicine actively involved in resident education. To assess the clinical performance of ChatGPT and physicians, the professor reviewed all conversations and assigned scores from 3 perspectives for each consultation on a 5-point Likert scale (with 1 being very poor and 5 being excellent). These scores included a history-taking score for the thoroughness of information gathering, a clinical accuracy score evaluating the medical correctness of diagnoses and treatment plans, and an empathy score reflecting the degree of attentiveness and responsiveness to the patient’s concerns. For the written examination, which included 3 questions on diagnosis, investigation, and treatment planning, responses from both ChatGPT and the physicians were evaluated by the emergency medicine professor. Scores were assigned for accuracy on a 6-point Likert scale (where 1 indicated incorrect and 6 indicated completely correct) and for completeness on a 3-point Likert scale (where 1 indicated incomplete and 3 indicated comprehensive).

In addition, all virtual patients participating in the CPX completed a 5-point scale survey consisting of 7 questions to evaluate the consultations from multiple perspectives. They assessed the overall comprehensibility and credibility of the consultation, as well as its effectiveness in alleviating patient concerns. Patients also evaluated the credibility and communication of the diagnosis and treatment plan provided by either the physician or ChatGPT. Finally, patients were asked to assess whether the consulting entity seemed more like ChatGPT or a human doctor.

### Statistical Analysis

Scores were presented as mean and SD. We evaluated each performance domain separately rather than combining them into a single score. Mean scores for each domain were calculated separately and compared between ChatGPT and the human doctor group. Group comparisons were conducted for each domain independently using either independent-sample *t* tests or Mann-Whitney *U* tests according to the distribution of the data. In the written examinations, questions were further categorized into easy, medium, and hard levels based on the scores of the physicians. Performance between physicians and ChatGPT was then compared across these defined difficulty levels. A post hoc power analysis was conducted for the CPX outcomes evaluated by the emergency medicine professor based on the observed effect sizes. Effect sizes were estimated using Cohen *d* or rank-biserial correlation, and statistical power was estimated based on these observed effect sizes. A *P* value of less than .05 was considered statistically significant. All statistical analyses were performed using R software, version 4.3.1 (R Core Team).

## Results

### CPX

The results from the CPX of ChatGPT and physicians, based on grading by the emergency medicine professor and patient survey responses, are summarized in Table 2. ChatGPT scored significantly higher than physicians in history taking (mean 3.91, SD 0.67 vs mean 2.67, SD 0.78; *P*<.001). Although ChatGPT also achieved a higher score in clinical accuracy (mean 3.75, SD 0.45 vs mean 3.33, SD 0.98), the difference was not statistically significant (*P*=.25). In terms of empathy, ChatGPT scored an average of 4.50 (SD 0.67), whereas physicians scored 1.75 (SD 0.62), falling below the “poor” rating threshold of 2. This difference was statistically significant (*P*<.001), highlighting ChatGPT’s superior empathetic engagement. Post hoc power analysis revealed that the history taking and empathy scores had high statistical power (0.98 and 0.99, respectively). In contrast, the clinical accuracy score showed low power (0.22), indicating a limited ability to detect significant group differences for this outcome.

Results from a survey completed by a virtual patient immediately after each CPX session showed that ChatGPT scored slightly higher than the human physicians across all categories. However, a statistically significant difference was only observed in the concern-reduction score, which measured the extent to which the consultation alleviated the patient’s worries (ChatGPT: mean 4.33, SD 0.78 vs physicians: mean 3.58, SD 0.90; *P*=.04). In addition, for the similarity assessment score, which evaluated whether the consultant appeared more like ChatGPT or a human physician, no significant difference was found (*P*=.71). Full records of the conversations between ChatGPT and the physicians with virtual patients during the CPX, along with the scores assigned by the emergency medicine professor and their distributions, are available in Multimedia Appendix 3.

**Table 2.** Results from the clinical performance examination of ChatGPT and physicians (all scores were assigned using a 5-point Likert scale, where 1 signifies “very poor,” 2 “poor,” 3 “average,” 4 “good,” and 5 “excellent”; for the similarity score, the scale was as follows: 1 indicated “very similar to ChatGPT,” 2 “somewhat similar to ChatGPT,” 3 “neutral, neither ChatGPT nor a real doctor,” 4 “somewhat similar to a real doctor,” and 5 denoted “very similar to a real doctor”).

Score	ChatGPT, mean (SD)	Overall physicians (n=12), mean (SD)	*P* value	GP^a^ (n=4), mean (SD)	R3^b^ (n=4), mean (SD)	R4^c^ (n=4), mean (SD)
Grading results from an emergency medicine professor						
History taking score	3.91 (0.67)	2.67 (0.78)	<.001	2.75 (0.50)	2.75 (0.50)	2.50 (1.29)
Clinical accuracy score	3.75 (0.45)	3.33 (0.98)	.25	2.75 (0.96)	3.75 (0.50)	3.50 (1.29)
Empathy score	4.50 (0.67)	1.75 (0.62)	<.001	1.50 (0.58)	2.00 (0.82)	1.75 (0.50)
Survey results from virtual patients						
Overall consultation						
Comprehensibility score	4.67 (0.49)	4.50 (0.52)	.43	4.75 (0.50)	4.00 (0.00)	4.75 (0.50)
Credibility score	4.42 (0.51)	3.92 (0.79)	.10	4.00 (0.82)	3.50 (0.58)	4.25 (0.96)
Concern reduction score	4.33 (0.78)	3.58 (0.90)	.04	3.75 (0.50)	3.50 (1.29)	3.50 (1.00)
Diagnosis						
Evaluation score	4.75 (0.45)	4.41 (0.67)	.20	5.00 (0.00)	3.75 (0.50)	4.50 (0.58)
Investigation and treatment plan						
Credibility score	4.50 (0.52)	4.17 (0.83)	.36	4.00 (0.82)	4.00 (1.15)	4.50 (0.58)
Communication score	4.42 (0.79)	4.00 (0.95)	.26	3.75 (0.50)	3.50 (1.29)	4.75 (0.50)
Similarity						
Assessment score	3.50 (1.78)	3.25 (1.86)	.71	2.75 (2.06)	3.25 (2.06)	3.75 (1.89)

a GP: general practitioner.

b R3: third-year emergency medicine resident.

c R4: fourth-year emergency medicine resident.

### Written Examinations

**Figure 2.**
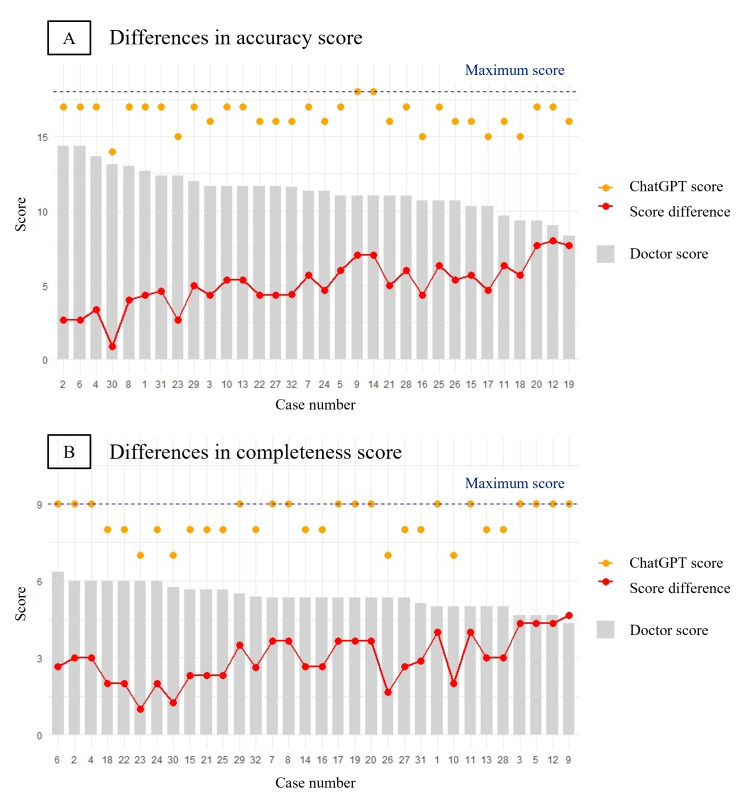
Difference of accuracy and completeness scores of ChatGPT and physicians from written examination.

The grading outcomes of the written examination are summarized in Table 3. ChatGPT achieved higher scores across all categories—diagnosis, investigation, and treatment plan—than the physicians. The mean accuracy scores for ChatGPT were 5.44 (SD 0.72), 5.38 (SD 0.55), and 5.59 (SD 0.50), significantly exceeding the physicians’ scores of 4.11 (SD 1.10), 3.67 (SD 0.93), and 3.74 (SD 0.98), respectively (*P*<.001 for all comparisons). Similarly, ChatGPT’s mean completeness scores were substantially higher, with averages of 2.84 (SD 0.37), 2.56 (SD 0.50), and 2.94 (SD 0.25), compared with the physicians’ scores of 1.84 (SD 0.45), 1.84 (SD 0.37), and 1.72 (SD 0.45) for each category, respectively (*P*<.001 for all). Detailed accuracy and completeness scores of the physicians and ChatGPT for all cases are presented in Figure 2. Complete answer sheets for the written examination provided by the physicians and ChatGPT and the scores assigned by the professor and their distributions are all included in Multimedia Appendix 4.

**Figure 3.**
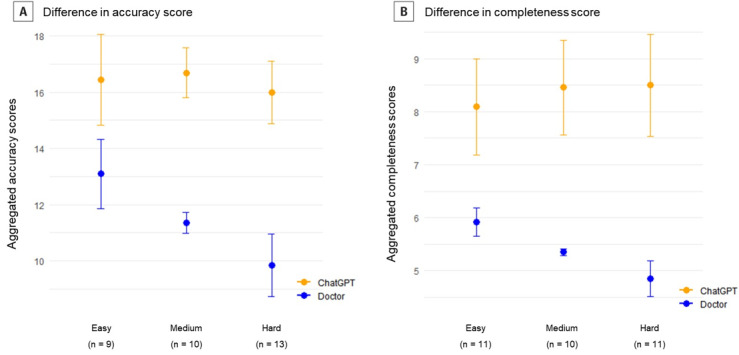
Difference in accuracy and completeness scores of ChatGPT and physicians categorized by difficulty level of the questions.

The differences in scores between ChatGPT and the physicians were further analyzed across various difficulty levels of the written examination questions (Figure 3). The scores for both accuracy and completeness were significantly higher for ChatGPT than for the physicians across all difficulty levels (*P*<.001, for all). Notably, the gap in scores between ChatGPT and the physicians widened on questions of higher difficulty, where the physicians’ scores were lower.

**Table 3.** Results from the written examination of ChatGPT and physicians. The accuracy scale was a 6-point Likert scale (with 1 indicating totally incorrect; 2, mostly incorrect; 3, balance of correct and incorrect; 4, more correct than incorrect; 5, almost entirely correct; and 6, completely correct), and a 3-point scale for completeness (1 for incomplete, 2 for adequate, and 3 for comprehensive).

Score	Participant group, mean (SD)		*P* value	GP^a^ (n=4), mean (SD)	R3^b^ (n=4), mean (SD)	R4^c^ (n=4), mean (SD)
	ChatGPT	Overall physicians (n=12)				
All (n=32)						
Diagnosis						
Accuracy score	5.44 (0.72)	4.11 (1.10)	<.001	3.14 (1.21)	4.36 (0.94)	4.45 (0.79)
Completeness score	2.84 (0.37)	1.84 (0.45)	<.001	1.61 (0.50)	2.02 (0.40)	1.82 (0.39)
Investigation						
Accuracy score	5.38 (0.55)	3.67 (0.93)	<.001	2.96 (0.84)	3.93 (0.85)	3.82 (0.95)
Completeness score	2.56 (0.50)	1.84 (0.37)	<.001	1.79 (0.42)	1.91 (0.29)	1.80 (0.41)
Treatment plan						
Accuracy score	5.59 (0.50)	3.74 (0.98)	<.001	2.96 (0.96)	3.93 (0.85)	4.05 (0.86)
Completeness score	2.94 (0.25)	1.72 (0.45)	<.001	1.68 (0.48)	1.73 (0.45)	1.73 (0.45)
Text-based questions (n=28)						
Diagnosis						
Accuracy score	5.46 (0.58)	3.96 (1.13)	<.001	3.14 (1.21)	4.21 (0.96)	4.54 (0.69)
Completeness score	2.86 (0.36)	1.82 (0.47)	<.001	1.61 (0.50)	2.04 (0.43)	1.82 (0.39)
Investigation						
Accuracy score	5.43 (0.57)	3.61 (0.97)	<.001	2.96 (0.84)	3.96 (0.79)	3.89 (0.96)
Completeness score	2.57 (0.50)	1.86 (0.35)	<.001	1.79 (0.42)	1.93 (0.26)	1.86 (0.36)
Treatment plan						
Accuracy score	5.57 (0.50)	3.67 (0.97)	<.001	2.96 (0.96)	4.00 (0.72)	4.04 (0.84)
Completeness score	2.96 (0.19)	1.70 (0.46)	<.001	1.68 (0.48)	1.71 (0.46)	1.71 (0.46)
Image-included questions (n=4)						
Diagnosis						
Accuracy score	5.25 (1.50)	4.50 (0.92)	.09	—^d^	4.62 (0.89)	4.38 (0.96)
Completeness score	2.75 (0.50)	1.91 (0.39)	<.001	—	2.00 (0.37)	1.81 (0.40)
Investigation						
Accuracy score	5.00 (0.00)	3.84 (0.81)	.005	—	4.00 (0.63)	3.69 (0.95)
Completeness score	2.50 (0.58)	1.78 (0.42)	.01	—	1.88 (0.34)	1.69 (0.48)
Treatment plan						
Accuracy score	5.75 (0.50)	3.94 (0.98)	.002	—	3.81 (1.05)	4.06 (0.93)
Completeness score	2.75 (0.50)	1.75 (0.44)	.001	—	1.75 (0.45)	1.75 (0.45)

a GP: general practitioner.

b R3: third-year emergency medicine resident.

c R4: fourth-year emergency medicine resident.

d Not applicable.

## Discussion

### Principal Findings

In this study, we compared the performance of ChatGPT and human doctors using 2 evaluation formats—CPX and written examinations. In the CPX, evaluations were conducted by an emergency medicine professor and virtual patients. ChatGPT received significantly higher scores than human doctors in the domains of history taking and empathy. Although ChatGPT showed a higher mean score in clinical accuracy, the difference was not statistically significant.

In the virtual patient evaluations, ChatGPT achieved higher mean scores across all domains, including comprehensibility, credibility, and communication. However, a statistically significant difference was observed only in the concern reduction domain. When virtual patients were asked to evaluate human-likeness, there was no significant difference between ChatGPT and human doctors, indicating that patients did not perceive a substantial distinction in human-likeness between the two.

Regarding the written examinations, ChatGPT demonstrated significantly higher scores than human doctors in most domains evaluated by the emergency medicine professor, including diagnosis, investigation, and treatment planning. This trend was consistent across both text-based and image-based clinical questions. In addition, when questions were stratified based on difficulty levels determined by human doctor scores, ChatGPT’s performance remained relatively stable, showing minimal variation according to question difficulty.

### Comparison With Previous Work

The clinical utility of ChatGPT and other LLMs has been increasingly investigated in the field of emergency medicine. Previous studies have demonstrated that LLMs can effectively support patient triage and identify cases requiring critical care based on clinical scenarios, highlighting their potential to assist in acute decision-making processes [20,21]. In addition, ChatGPT has shown promising performance in interpreting electrocardiogram-related questions and providing reliable guidance for imaging test referrals [22,23]. Our findings from the written examinations are consistent with previous studies, further supporting the potential of ChatGPT as a clinical decision support tool in emergency medicine. We extended previous research by evaluating not only the accuracy but also the completeness of responses in open-ended, case vignette–based assessments. A similar finding was reported in a previous study, in which ChatGPT provided more extended responses than those of physicians without compromising quality when answering patients’ questions on a public online forum [5]. This ability to generate comprehensive answers without relying on predefined options is promising for the future application of ChatGPT in real clinical settings within emergency medicine.

However, most previous studies have relied on examination-based assessments, which may oversimplify clinical reasoning by limiting diagnostic flexibility and excluding the iterative, hypothesis-driven nature of real-world medical consultations and patient interactions [24]. Our study addresses this limitation by implementing a CPX format, which enables a more in-depth evaluation of AI performance in scenarios that closely resemble actual clinical practice. This methodology allowed us to assess ChatGPT’s capabilities in a dynamic, human-like context that simulates instantaneous decision-making.

In this study, we found that ChatGPT performed significantly better in history taking, while demonstrating clinical accuracy comparable to that of human doctors. Although previous studies have examined the use of chatbots for history taking, most were developed for narrowly defined purposes or relied on fixed sets of predefined questions presented in conversational form [25]. In contrast, our findings show that a general-purpose conversational AI can actively engage in clinical reasoning and elicit relevant patient history through interactive dialogue. This suggests that the integration of such AI tools into clinical workflows may enhance the quality of history taking and ultimately improve patient care.

We also found that ChatGPT received higher empathy scores and greater concern reduction ratings from patients. More empathetic responses from ChatGPT compared with human doctors have also been observed in previous studies [5,26], and our findings extend this evidence by evaluating full conversational interactions. These results suggest that AI models may not only support clinical reasoning but also complement physicians in areas often compromised under high-stress conditions, such as patient-centered communication and emotional engagement.

Interestingly, patients were unable to distinguish whether they were interacting with ChatGPT or a human physician, yet the resulting empathy scores were significantly higher for ChatGPT. This suggests that communication quality may outweigh the communicator’s identity in shaping perceived empathy. However, previous studies indicate that patient satisfaction can decline when AI-generated responses are explicitly disclosed, underscoring the complexity of transparency in clinical AI use [27]. Determining when and how to disclose AI involvement remains an important ethical and practical challenge, warranting further research into its impact on patient trust and acceptance.

### Ethical and Educational Implications

While our study demonstrates that ChatGPT can perform reasonably well in clinically simulated settings, several barriers must be addressed before independent deployment in real-world practice [28]. A major concern is liability for inaccurate or harmful recommendations, with uncertainty over whether responsibility would fall on software developers, health care providers, or end users [29,30]. Furthermore, the black-box nature of LLMs makes it difficult even for professionals to fully evaluate AI-generated outputs [31]. Robust legal and ethical frameworks will be essential for the safe integration of AI into clinical care [32].

Data privacy also presents a critical challenge. Many LLMs, including ChatGPT, operate through cloud-based services, raising concerns about the transmission and storage of sensitive health information [33,34]. One potential solution is the development of open-source models that can operate within closed, local systems without cloud dependence. However, further research is needed to assess the feasibility, security, and clinical applicability of such approaches.

With the growing integration of AI into clinical practice, its role in medical education has also expanded [35]. AI models such as ChatGPT have been used not only to deliver factual knowledge but also to enhance patient communication skills. They can be useful for simulating patient interactions and providing immediate feedback [36]. However, concerns remain that excessive reliance on AI may undermine the development of independent critical thinking and clinical reasoning skills [37]. To address this, AI-integrated curricula must be designed to harness the strengths of AI while actively cultivating human competencies in critical thinking, clinical judgment, and ethical decision-making [38]. Balancing these elements will be essential to maintain independent expertise in an increasingly AI-augmented health care environment.

### Limitations

This study has several limitations. First, the CPX format used in this study does not fully replicate the real-world clinical environment in which physicians interact with patients in the emergency department. The simulations were conducted via text-based messenger in a blinded format, which limited access to nonverbal cues and other clinically relevant information typically available during face-to-face encounters. As a result, participants could not demonstrate the full range of clinical skills required in actual patient care. In addition, while moderation was necessary to anonymize participants and correct typographical errors, the process occasionally involved segmenting and restructuring physicians’ input for clarity. These adjustments may have disrupted the natural flow of conversation and removed pauses and hesitations, which are essential for conveying empathy [39]. For example, splitting long, intricate sentences into shorter, specific segments can give patients the impression that the conversation is overly focused on medical facts and interaction is less patient-centered. Future research should compare human performance and AI in more realistic settings including voice-based or in-person interactions to reflect nonverbal cues and conversational dynamics. Second, all CPX evaluations were performed by a single emergency medicine professor. Although the assessor was experienced, the use of a single evaluator may have introduced bias or subjectivity in scoring, particularly in domains such as empathy or communication quality. To address this issue, the evaluator calibrated the scoring process by applying the rubric to CPX manuscripts previously used for educational purposes and discussing borderline ratings with other physician investigators to ensure consistent interpretation. Future research should involve multiple independent raters to reduce subjectivity and enhance scoring consistency. Third, the relatively small number of participants may have limited the statistical power to detect modest differences between groups. Larger studies are needed to validate and extend these findings. Fourth, the clinical scenarios used in both CPX and written examinations were limited to a predefined set of representative cases. While selected to reflect a range of emergency conditions, the findings may not be generalizable to all clinical contexts or specialties. Future studies should include a broader range of clinical situations and patient presentations to enhance generalizability. Finally, this study did not include structured interviews or surveys of participating physicians to capture their perspectives on ChatGPT. Instead, we conducted brief debriefings after CPX sessions, which revealed that physicians initially underestimated ChatGPT’s capabilities. They highlighted its continuous availability and rapid response generation as key strengths and noted that lower empathy scores served as a reminder of the importance of empathic communication in practice. Clinicians also emphasized that legal and liability issues must be resolved before broader AI adoption. Future research should incorporate formal clinician interviews to systematically explore these insights and guide safe AI integration.

### Conclusion

In conclusion, the AI model demonstrated robust capabilities in clinical reasoning, diagnostic accuracy, and empathic communication in simulated emergency medicine scenarios. These findings highlight the potential of AI to serve as a supportive role in clinical practice. Future efforts should focus on developing collaborative frameworks that integrate AI into existing clinical workflows and harness AI’s strengths safely and effectively in real-world practice.

## Supplementary material

10.2196/68409 Multimedia Appendix 1 Publication consent.

10.2196/68409 Multimedia Appendix 2 Instructions for ChatGPT, physicians, and virtual patients for clinical performance examination and written examinations.

10.2196/68409 Multimedia Appendix 3 Full clinical performance examination transcripts and grading results.

10.2196/68409 Multimedia Appendix 4 Written examination answer sheets and grading results.

## Abbreviations

AI: artificial intelligence
CPX: clinical performance examination
GP: general practitioner
LLM: large language model
