# Supplementary figures and images for: Clinical Performance and Communication Skills of ChatGPT Versus Physicians in Emergency Medicine: Simulated Patient Study
Source: JMIR Med Inform. 2025 Jul 17;13:e68409. doi: 10.2196/68409 (PMC12289221; doi:10.2196/68409)

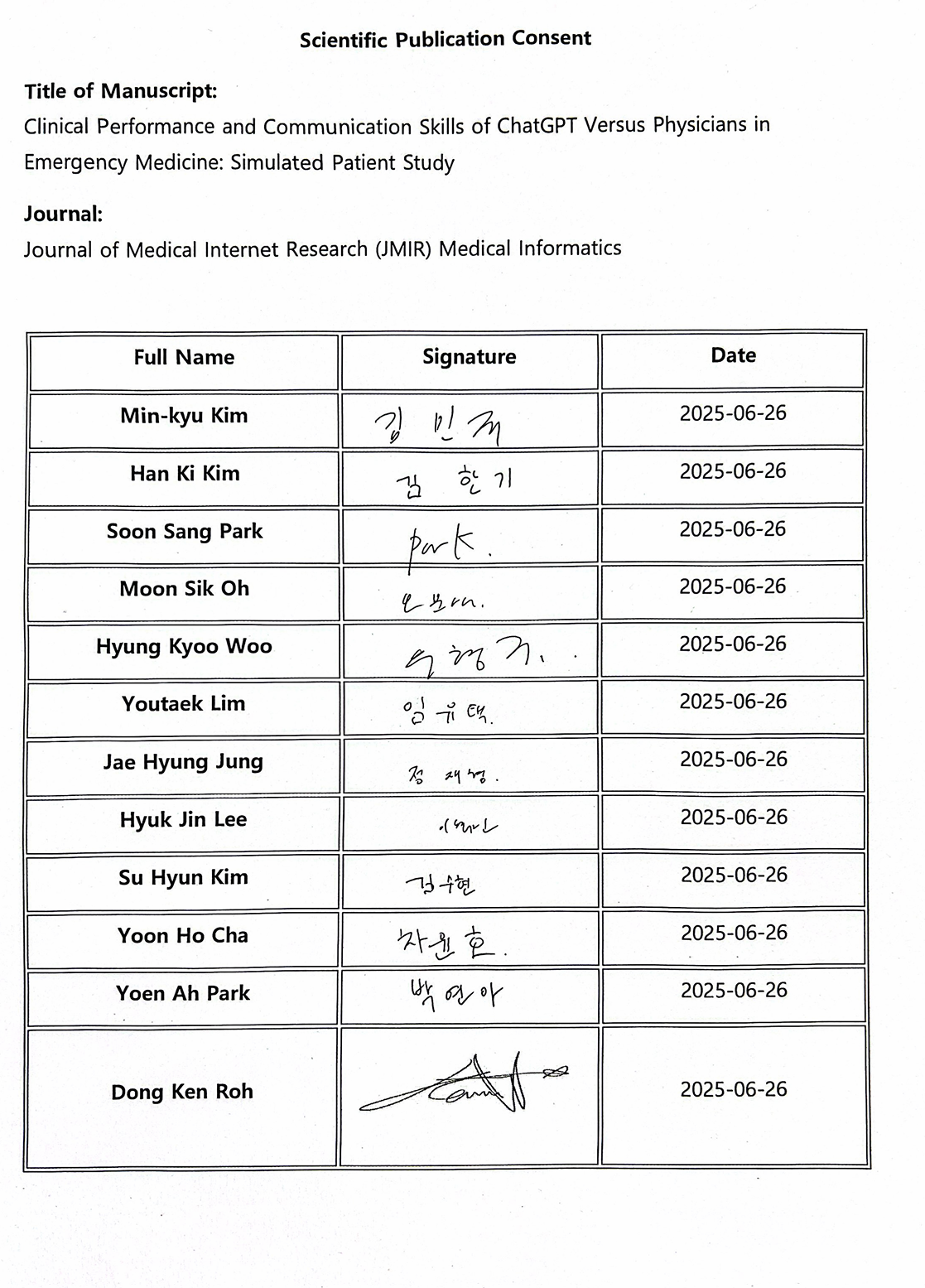

Supplement: Multimedia Appendix 1 [file medinform-v13-e68409-s001.png]
