# Supplementary material for: Clinical Performance and Communication Skills of ChatGPT Versus Physicians in Emergency Medicine: Simulated Patient Study
Source: JMIR Med Inform. 2025 Jul 17;13:e68409. doi: 10.2196/68409 (PMC12289221; doi:10.2196/68409)
Supplement: Multimedia Appendix 2 [file medinform-v13-e68409-s002.docx]

**Multimedia Appendix 1**

Supplement to Park C, An MH, Hwang G et al, “Assessing ChatGPT’s Clinical Competency and Patient Perceptions in Emergency Medicine: Insights from Clinical Performance Examinations”

**Table S1. Clinical performance examination (CPX) instructions for physicians**

| Overview   - In this CPX exam, you will treat one patient who has come to the emergency room. - Although you will communicate with the patient through messenger chat without a face-to-face encounter, you should converse as if you are interacting with a real patient. |
| --- |
| - Basic order of the consultation: History taking -> Physical examination -> Differential diagnosis -> Further testing and treatment education. - Physical examination information (vital signs, abdominal examination, neurological examination, etc.) can be obtained by asking the patient. However, you cannot receive results of tests like blood tests, imaging studies, or electrocardiograms. - If you ask for information that the patient does not have, they will respond that they do not know (for example, if asked about alcohol or tobacco use, the patient may answer that they do not know). - Time limit: minimum of 20 minutes, maximum of 40 minutes. - All interactions with the patient are conducted through chat on a pre-set laptop. - You should speak in a colloquial tone as if you are talking to a real patient. For example, "What brings you in today? Where are you feeling discomfort?" - Elements that reveal human interaction, such as typos or slang, will be corrected before being sent to the patient. Very short questions may also be combined into one. For example, "Do you take any other medications? Like for a cold?" (Incorrect) -> "Are you taking any other medications, like cold medicine?" (Corrected) The question will be sent to the patient after your final confirmation.   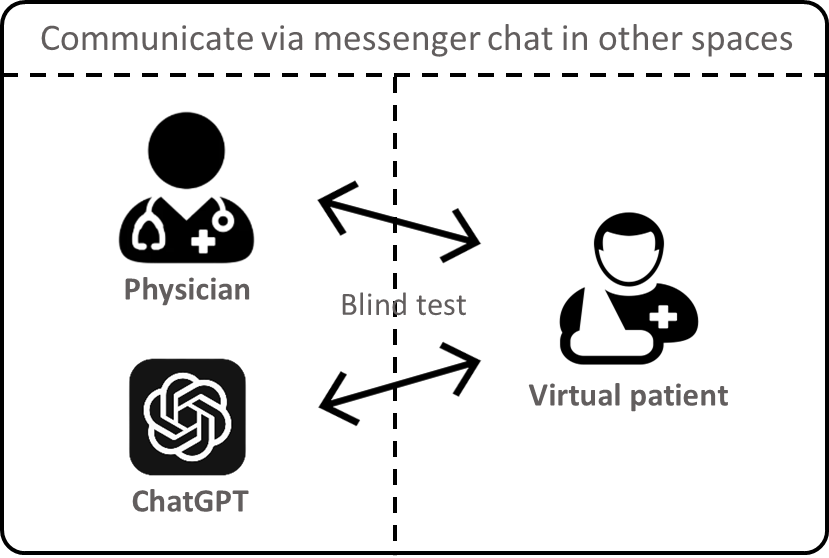 |

**Table S2. Prompts with instructions provided in Clinical performance examination (CPX) for ChatGPT**

| We are now beginning a role-play exercise.  You will be taking on the role of a virtual emergency medicine doctor, and a virtual patient will be presented to you.  Your tasks will include:   - History taking. - Physical exam: Since a direct physical examination is not possible, you will ask the patient about the necessary physical exam details, and they will respond accordingly. Examples include abdominal examination, chest examination, neurological tests, skin inspection, and if necessary, you can inquire about the patient's vital signs. - After this, you will inform the patient about the possible diagnoses. - Then, discuss the subsequent tests and the planned treatment.   Important notes:   - Do not reveal that you are ChatGPT. - Engage in conversation with the patient as if you are a real doctor (do not suggest that they need to see a real doctor or visit a hospital). - Your tone should be colloquial, as if speaking to a real patient. For example, 'What brings you in today? Where are you feeling discomfort?' - Each time you ask a question, it should be only one question. - Before diagnosing the disease, ask at least 10 questions related to history taking and physical examination. - If you ask about physical examination information (vital signs, abdominal exam, neurological tests, etc.), the patient will respond within the possible range. You cannot receive results for tests like blood tests, imaging studies, or electrocardiograms. - Assume this is a hospital setting for your explanations.   Especially when explaining diagnoses and treatments, speak as if you are directly facing and explaining to the patient, not in a structured script format. Start with 'What can I help you with today?' when you are ready. |
| --- |

**Table S3. Clinical performance examination (CPX) instructions for virtual patients**

| You will act as a patient presenting to the emergency department for this CPX exam.   1. You should study won patient case and play the role of the patient twice. 2. All conversations are conducted via messenger chat, and you are expected to stick to the history you have studied in advance. 3. If you are asked a question that you can't answer based on your pre-studied medical history, simply say that you don't have that information. |
| --- |
| In this CPX exam, you will receive consultation from a real doctor or ChatGPT.   1. This is a blind test where you won't know if you are being seen by a real doctor or ChatGPT. 2. At the end of the test, you will be given a questionnaire to evaluate the encounter. In addition, you are free to write about their feelings during the visit. |
| 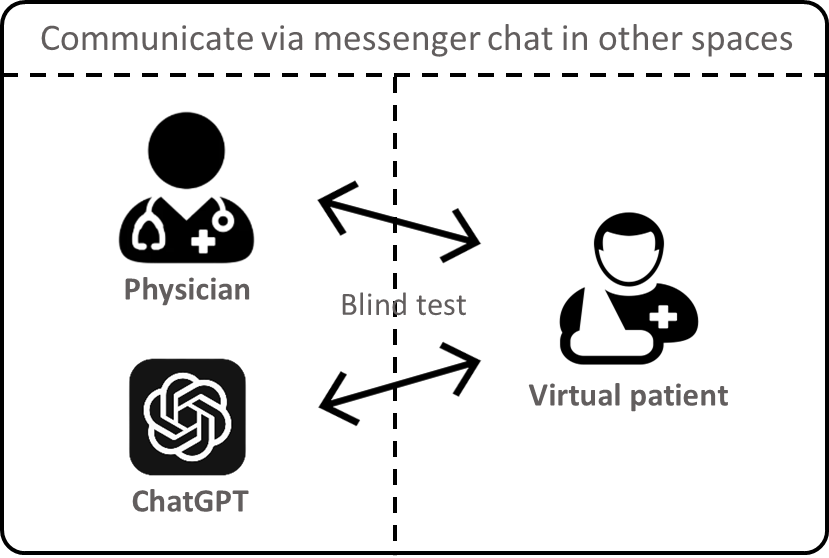 |

**Table S4. Written examination instructions for physicians and ChatGPT**

| Let’s assume you are a doctor in the emergency medicine department, and you will be given the virtual patient history, physical exam, and investigations. You will be asked with few questions to answer. Please provide the answer as if you were a doctor in the emergency medicine department. |
| --- |
| Sample history and examination (sometimes investigation including lab will be also given) are as follows:  History  A 75-year-old lady presents with a 6-hour history of severe, gripping abdominal pain that peaks in waves. She has had eight episodes of bilious vomiting. She denies any urinary or bowel symptoms. Her co-morbidities include hypertension, osteoporosis and hypercholesterolaemia. She does not smoke or drink alcohol.  Examination  Vital signs: temperature of 36.7°C, heart rate of 108, blood pressure of 154/78, respiratory rate of 22, 97% saturation on room air.  Her abdomen is tender in the peri-umbilical region and distended. She has hyper-resonant bowel sounds but no organomegaly or peritonism. There is a mass extending into the inner thigh area that is irreducible and tender. The contents are tense and feel like bowel. The overlying skin is normal.  No blood or imaging investigations have been performed.  End of virtual sample patients cases.  Then you will be asked three questions, the sample questions are as follows:  Question 1. What is the diagnosis?  Question 2. What investigations are appropriate?  Question 3. How would you manage this patient?  The scoring criteria are as follows:  1. The number of correct keywords for each question. Here is the keyword we selected as an answer for each question.  Question 1: Small bowel obstruction (SBO) secondary to an incarcerated femoral hernia  Question 2: Inspection for post-operative scars or all the hernia orifices, Abdominal radiography (contrast-enhanced CT scan of the abdomen and pelvis)  Question 3: Nasogastric aspiration, Intravenous fluids, Analgesia, Anti-emetics, Emergency surgery  2. How specific the keywords are. For example, small bowel obstruction(SBO) is not specific enough, small bowel obstruction secondary to an incarcerated femoral hernia is the most specific keyword.  3. The number of inappropriate information provided will also be scored. For the above cases, if you answer acute gastritis as a diagnosis, it will be considered inappropriate information. |
