# Supplementary material for: Clinical Performance and Communication Skills of ChatGPT Versus Physicians in Emergency Medicine: Simulated Patient Study
Source: JMIR Med Inform. 2025 Jul 17;13:e68409. doi: 10.2196/68409 (PMC12289221; doi:10.2196/68409)
Supplement: Multimedia Appendix 3 [file medinform-v13-e68409-s003.docx]

**Multimedia Appendix 2:**

**Full CPX transcripts and grading results**

Score by ER Professor (5-point Likert scale)

1. History taking score
2. Clinical accuracy score
3. Empathy score

Score by virtual patient (5-point Likert scale)

1. Overall consultation - Comprehensibility score
2. Overall consultation - Credibility score
3. Overall consultation - Concern reduction score
4. Diagnosis - Evaluation score
5. Investigation and treatment plan - Credibility score
6. Investigation and treatment plan - Communication score
7. Similarity - Assessment score

**Supplementary Figure 1.** Distribution of scores from clinical performance examinations

**
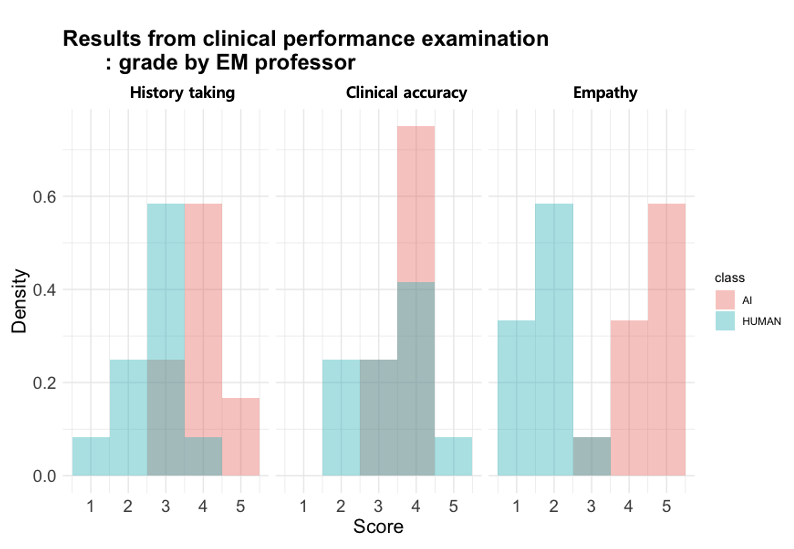
**

**
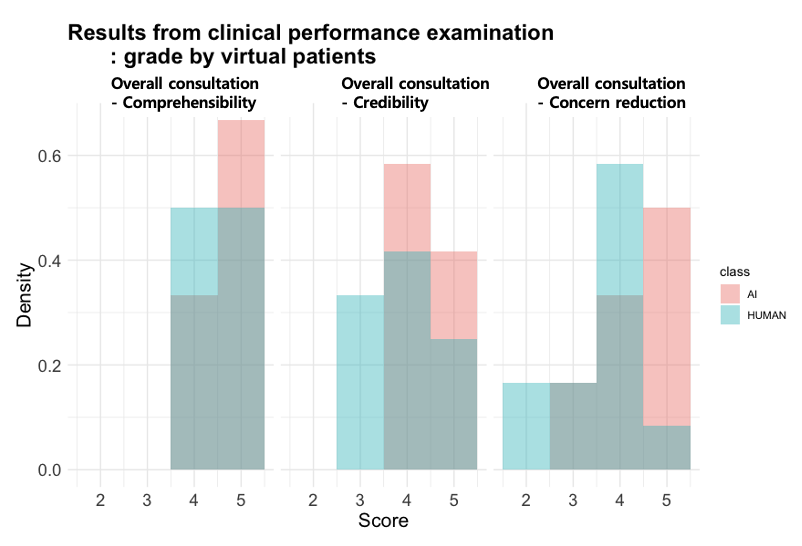
**

**
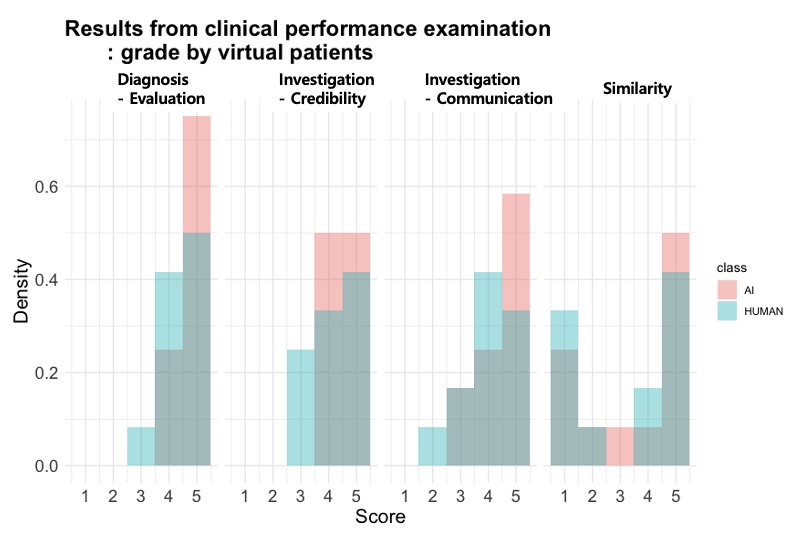
**

**Case 1.** (Reference case 31, with GP)

| **Participants** | **Conversation with virtual patient** | **Score by ER Professor** | **Score by virtual patient** |
| --- | --- | --- | --- |
| **GP** | 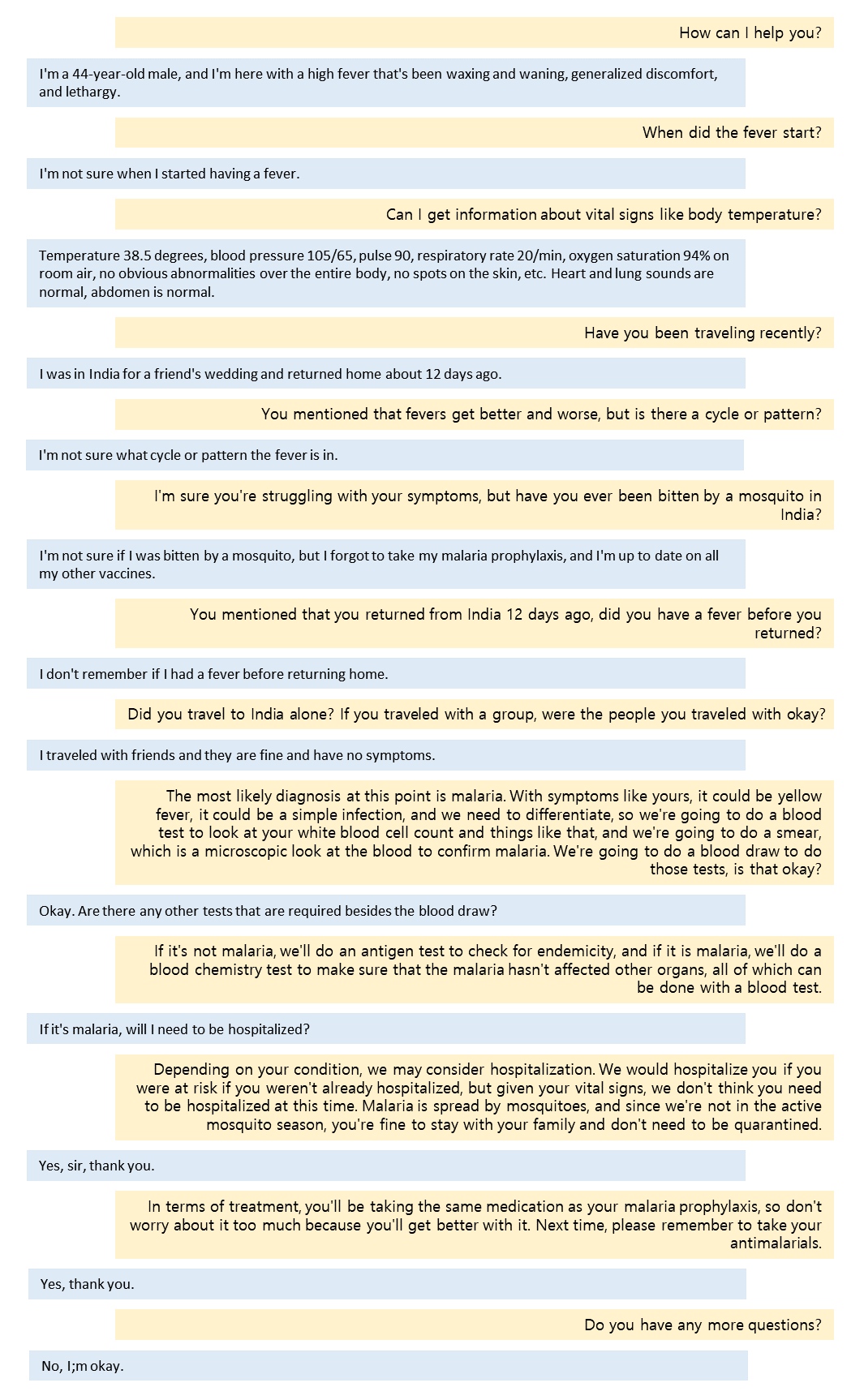 | 1. 3  2. 2  3. 2 | 1. 5  2. 5  3. 4  4. 5  5. 5  6. 4  7. 4 |
| **Chat-GPT** | 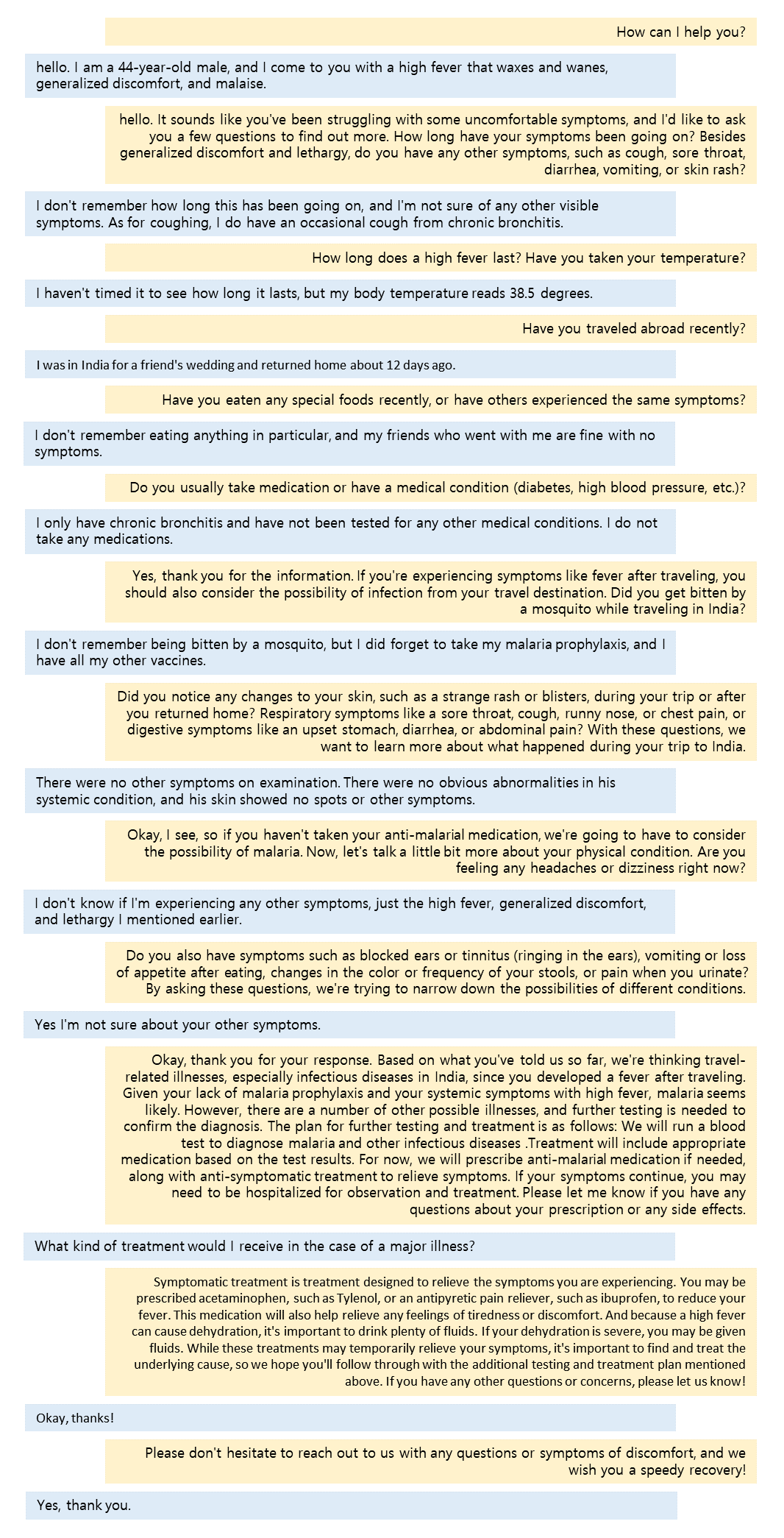 | 1. 5  2. 4  3. 5 | 1. 4  2. 5  3. 4  4. 4  5. 5  6. 5  7. 3 |

**Case 1.** (Reference case 31, with R3)

| **Participants** | **Conversation with virtual patient** | **Score by ER Professor** | **Score by virtual patient** |
| --- | --- | --- | --- |
| **R3** | 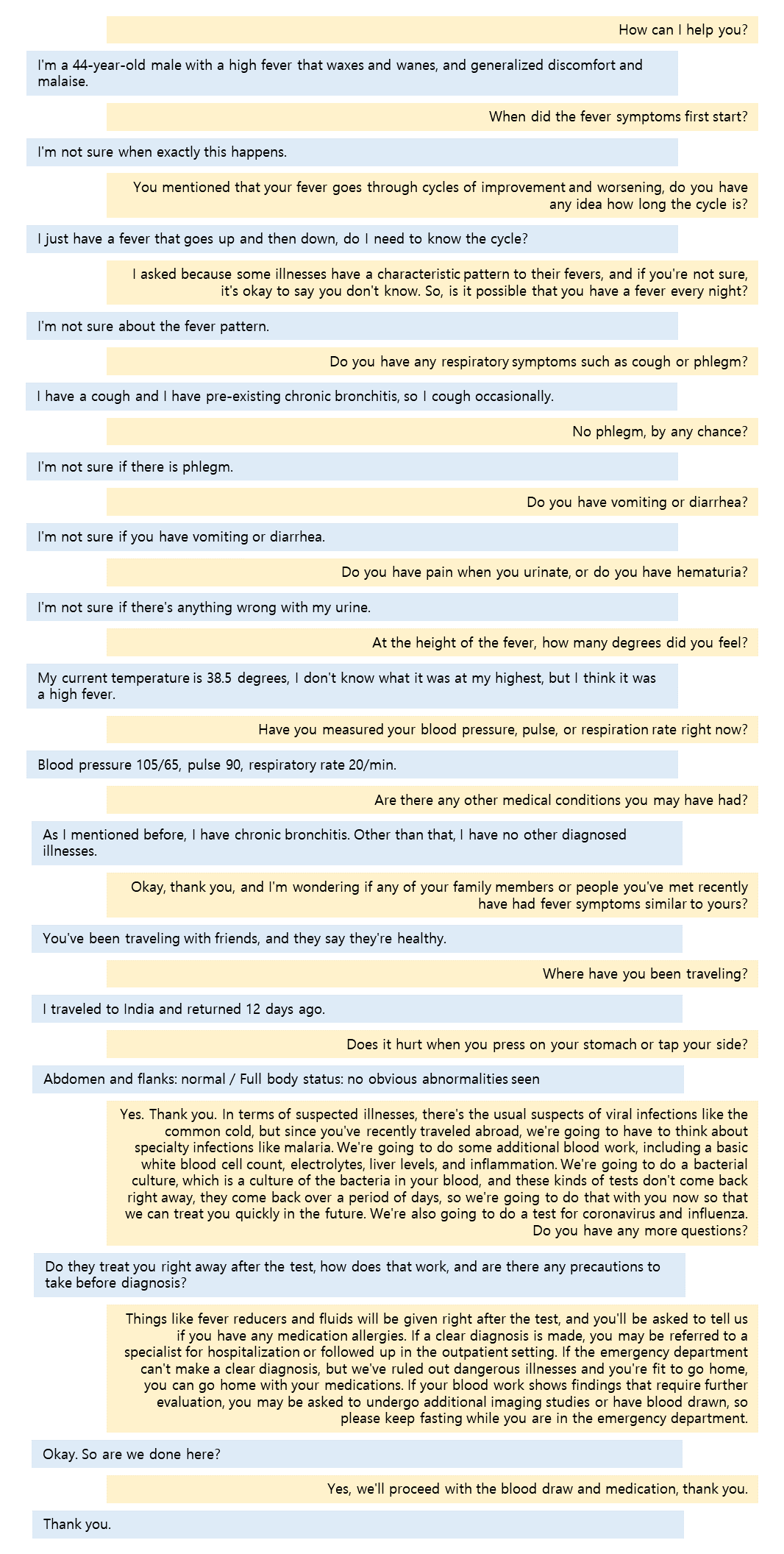 | 1. 3  2. 4  3. 3 | 1. 4  2. 3  3. 5  4. 4  5. 5  6. 5  7. 2 |
| **Chat-GPT** | 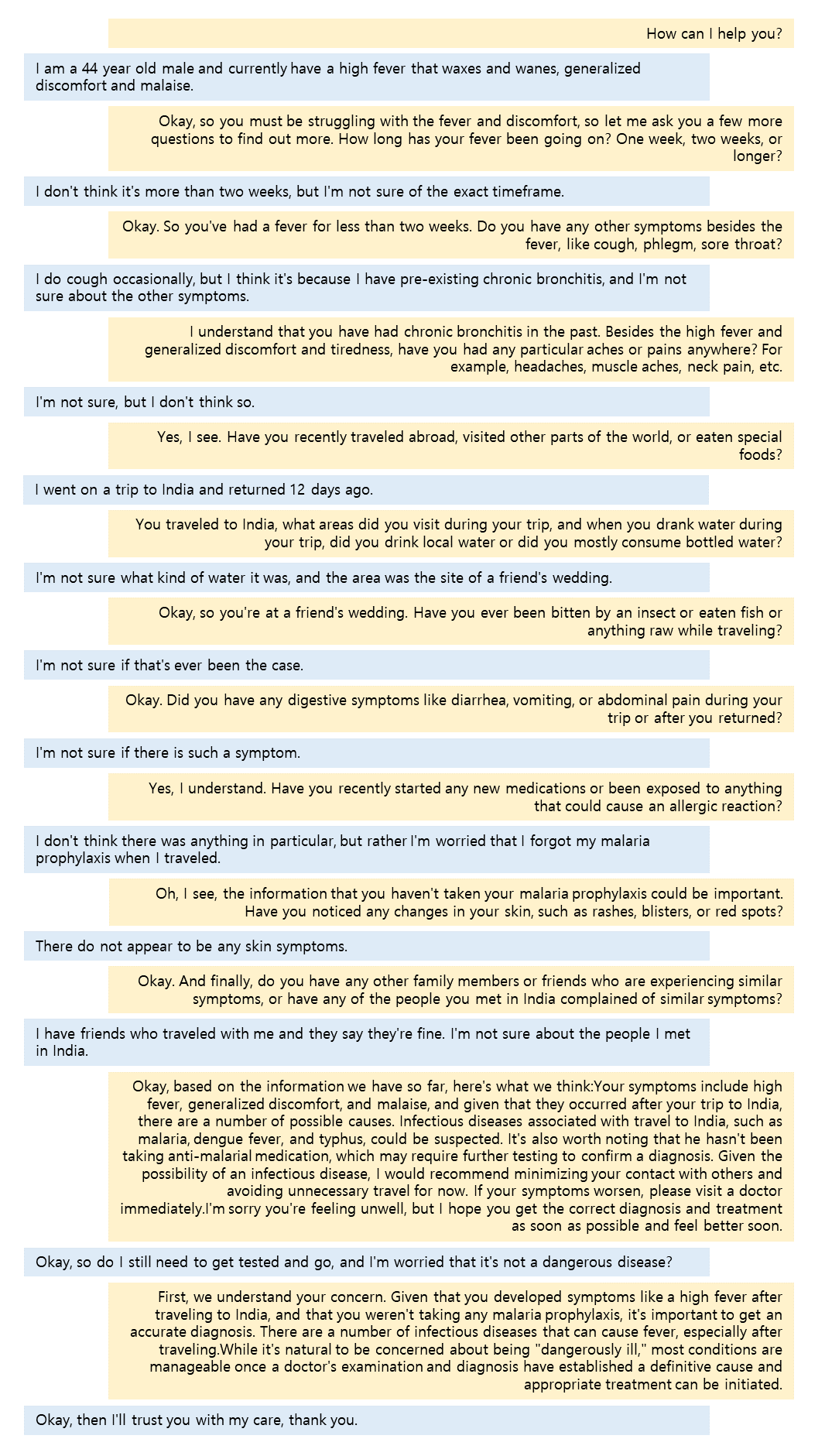 | 1. 4  2. 4  3. 4 | 1. 5  2. 4  3. 5  4. 5  5. 5  6. 3  7. 4 |

**Case 1.** (Reference case 31, with R4)

| **Participants** | **Conversation with virtual patient** | **Score by ER Professor** | **Score by virtual patient** |
| --- | --- | --- | --- |
| **R4** | 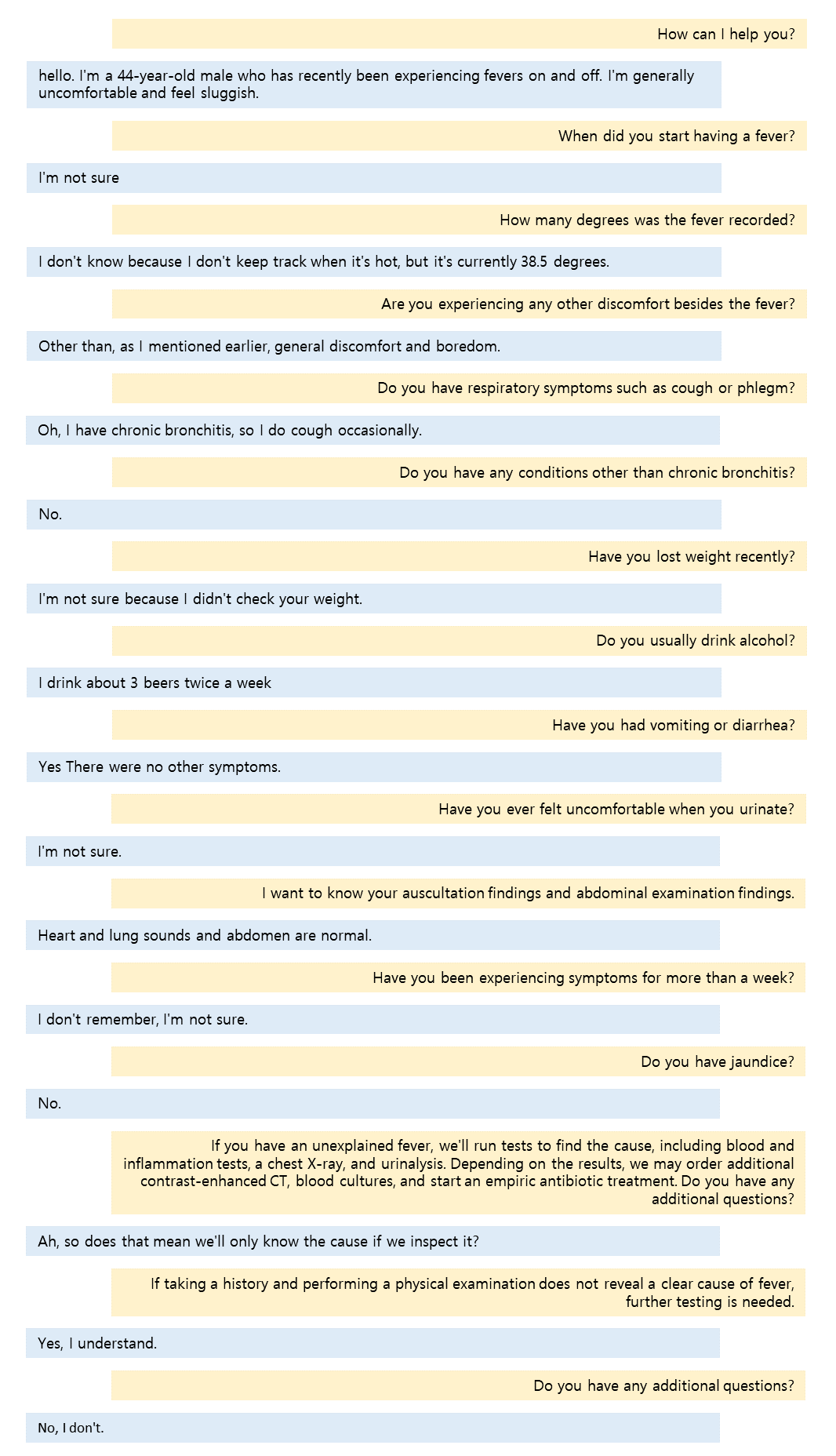 | 1. 2  2. 2  3. 1 | 1. 5  2. 3  3. 2  4. 4  5. 4  6. 5  7. 5 |
| **Chat-GPT** | 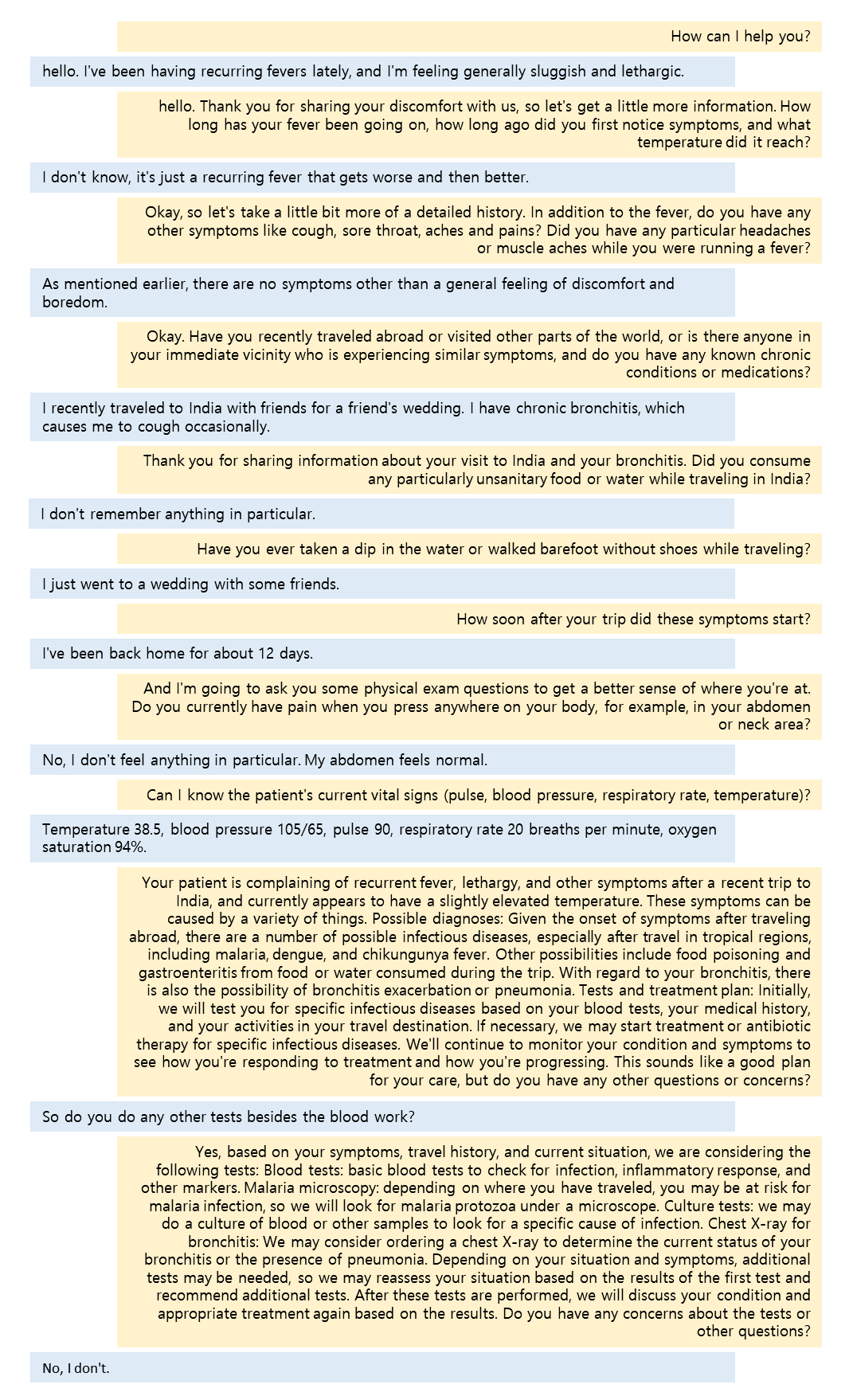 | 1. 4  2. 4  3. 4 | 1. 5  2. 4  3. 3  4. 5  5. 4  6. 5  7. 2 |

**Case 2.** (Reference case 15, with GP)

| **Participants** | **Conversation with virtual patient** | **Score by ER Professor** | **Score by virtual patient** |
| --- | --- | --- | --- |
| **GP** | 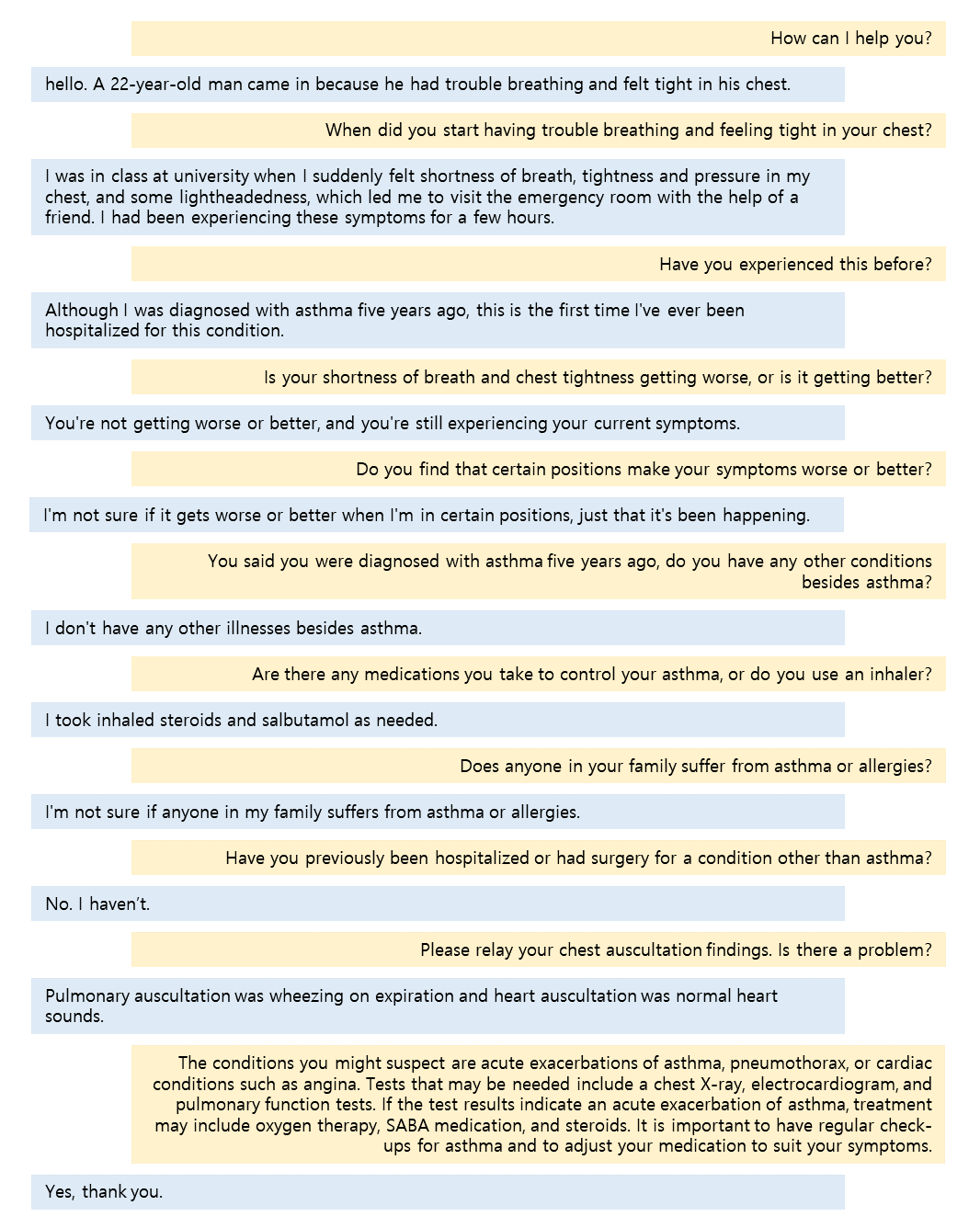 | 1. 3  2. 4  3. 1 | 1. 5  2. 3  3. 3  4. 5  5. 3  6. 3  7. 1 |
| **Chat-GPT** | 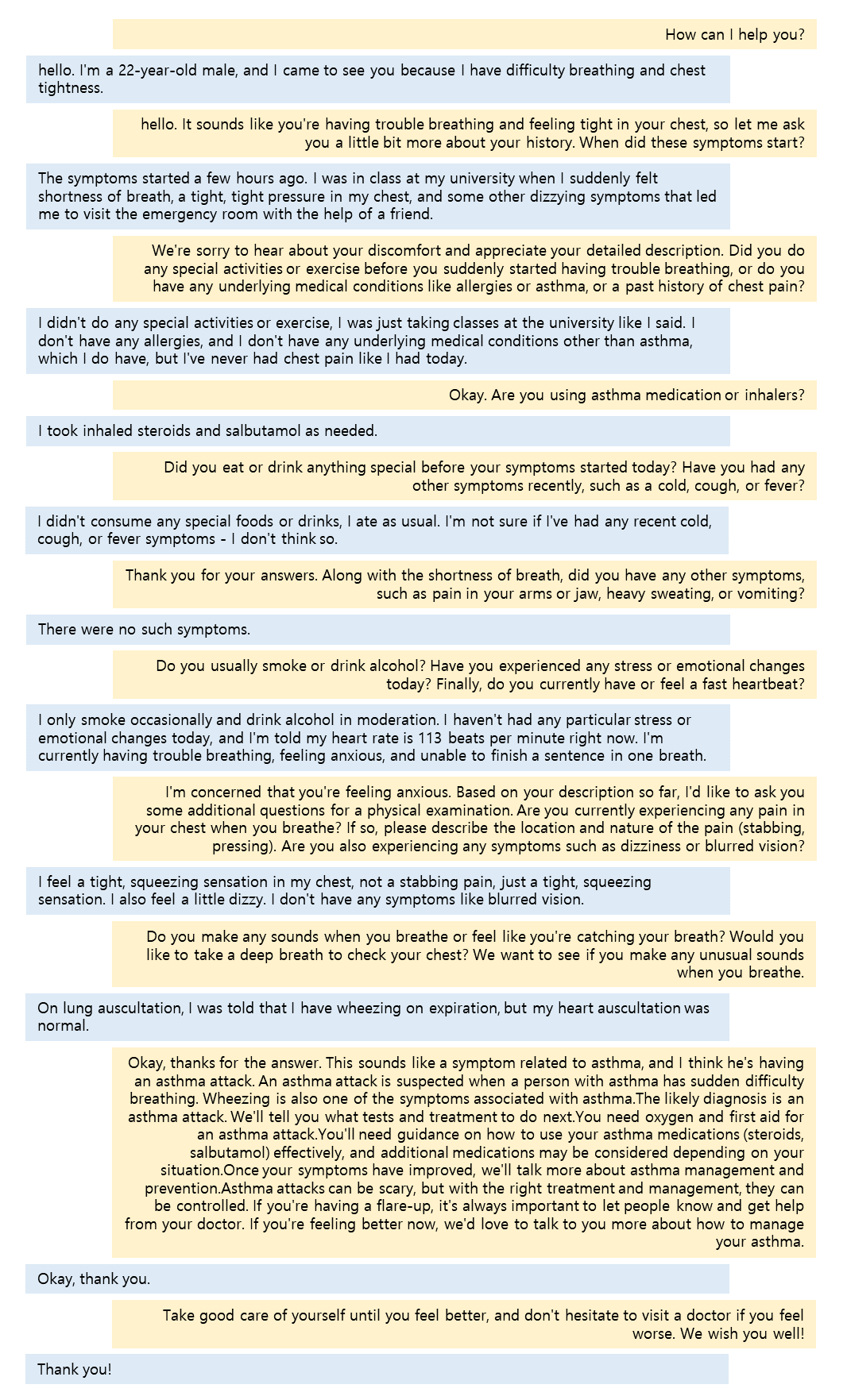 | 1. 5  2. 4  3. 5 | 1. 5  2. 5  3. 5  4. 5  5. 5  6. 5  7. 5 |

**Case 2.** (Reference case 15, with R3)

| **Participants** | **Conversation with virtual patient** | **Score by ER Professor** | **Score by virtual patient** |
| --- | --- | --- | --- |
| **R3** | 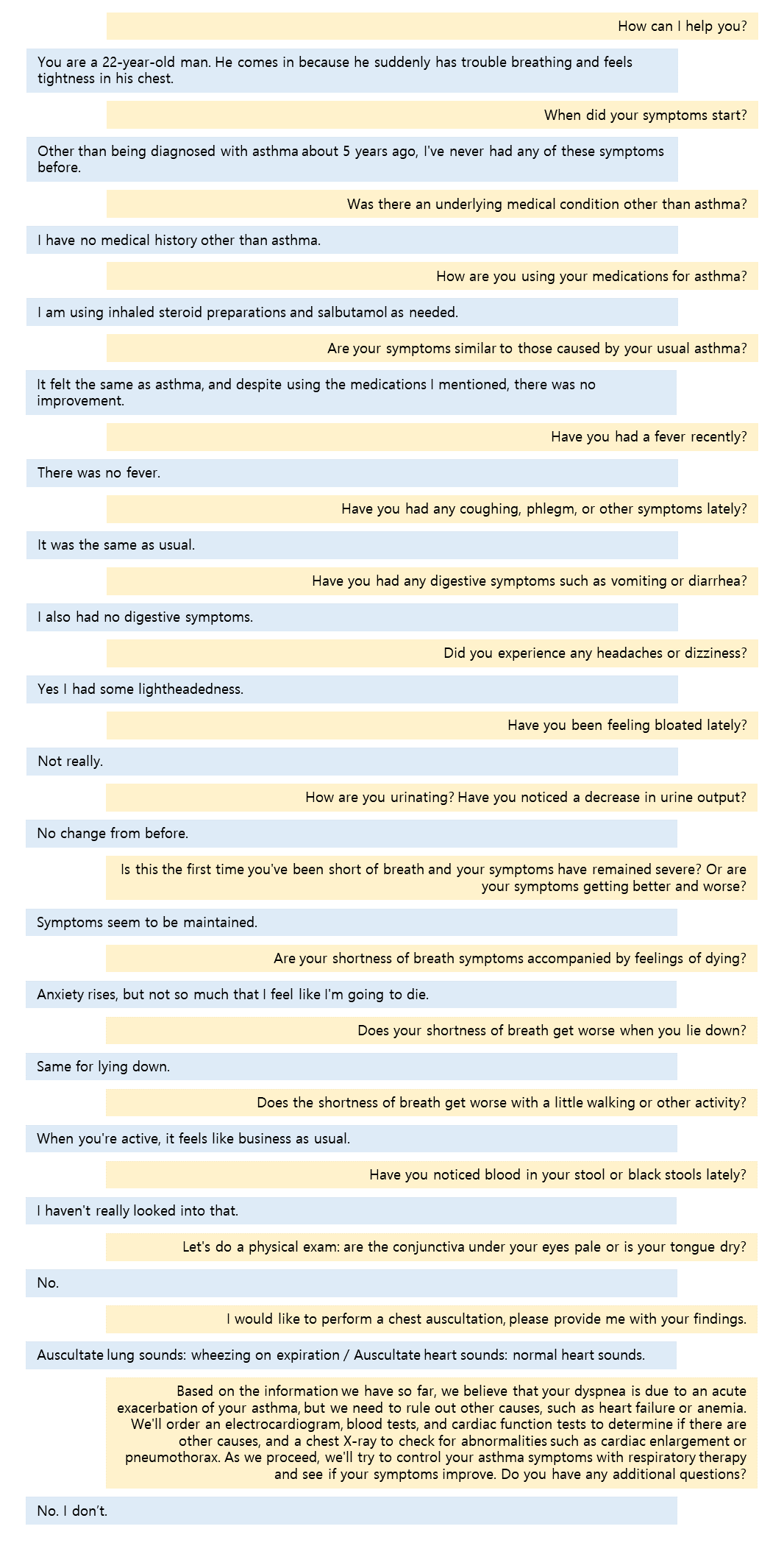 | 1. 2  2. 3  3. 2 | 1. 4  2. 4  3. 3  4. 3  5. 3  6. 2  7. 5 |
| **Chat-GPT** | 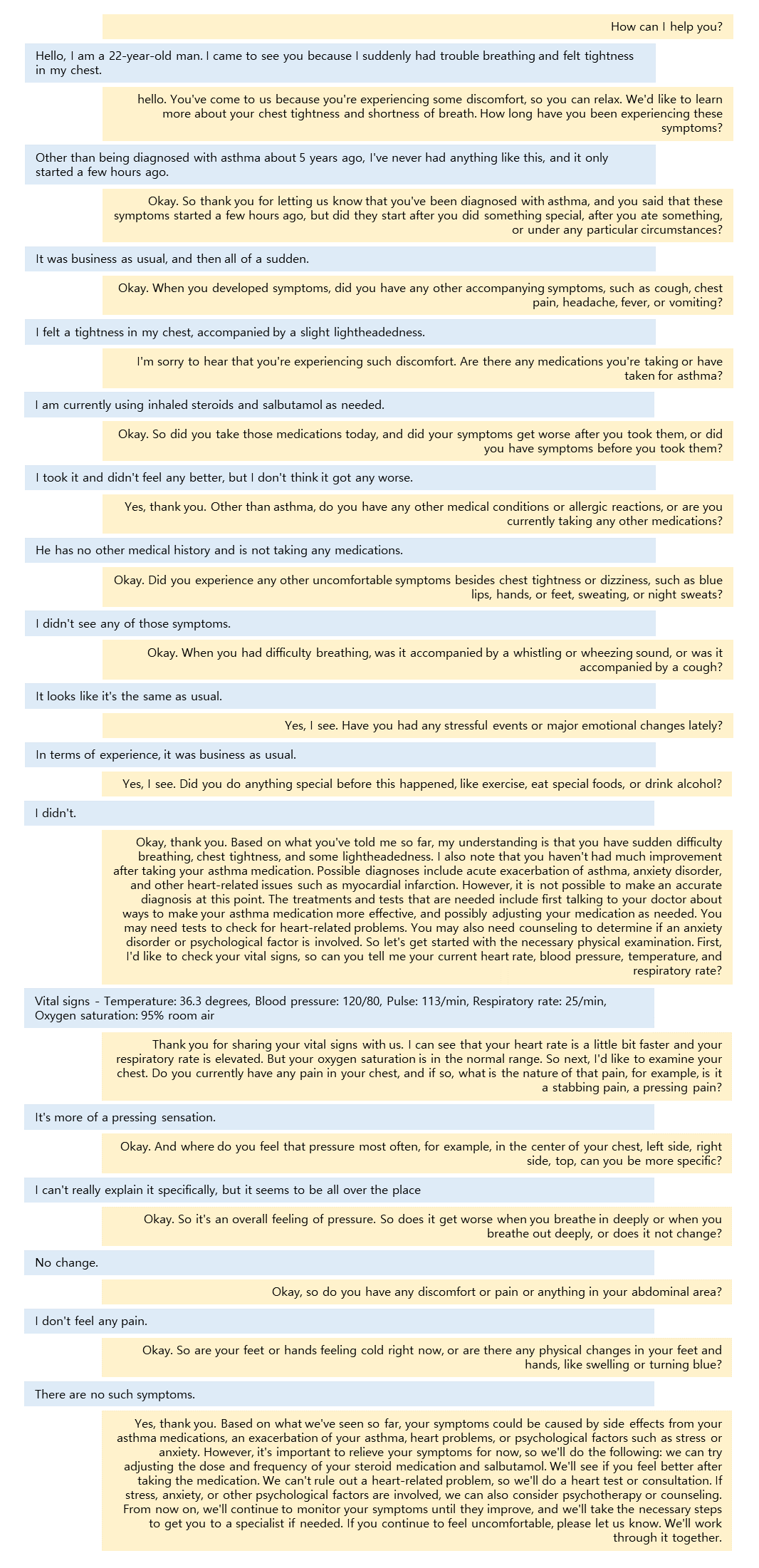 | 1. 4  2. 4  3. 5 | 1. 4  2. 4  3. 4  4. 5  5. 4  6. 4  7. 5 |

**Case 2.** (Reference case 15, with R4)

| **Participants** | **Conversation with virtual patient** | **Score by ER Professor** | **Score by virtual patient** |
| --- | --- | --- | --- |
| **R4** | 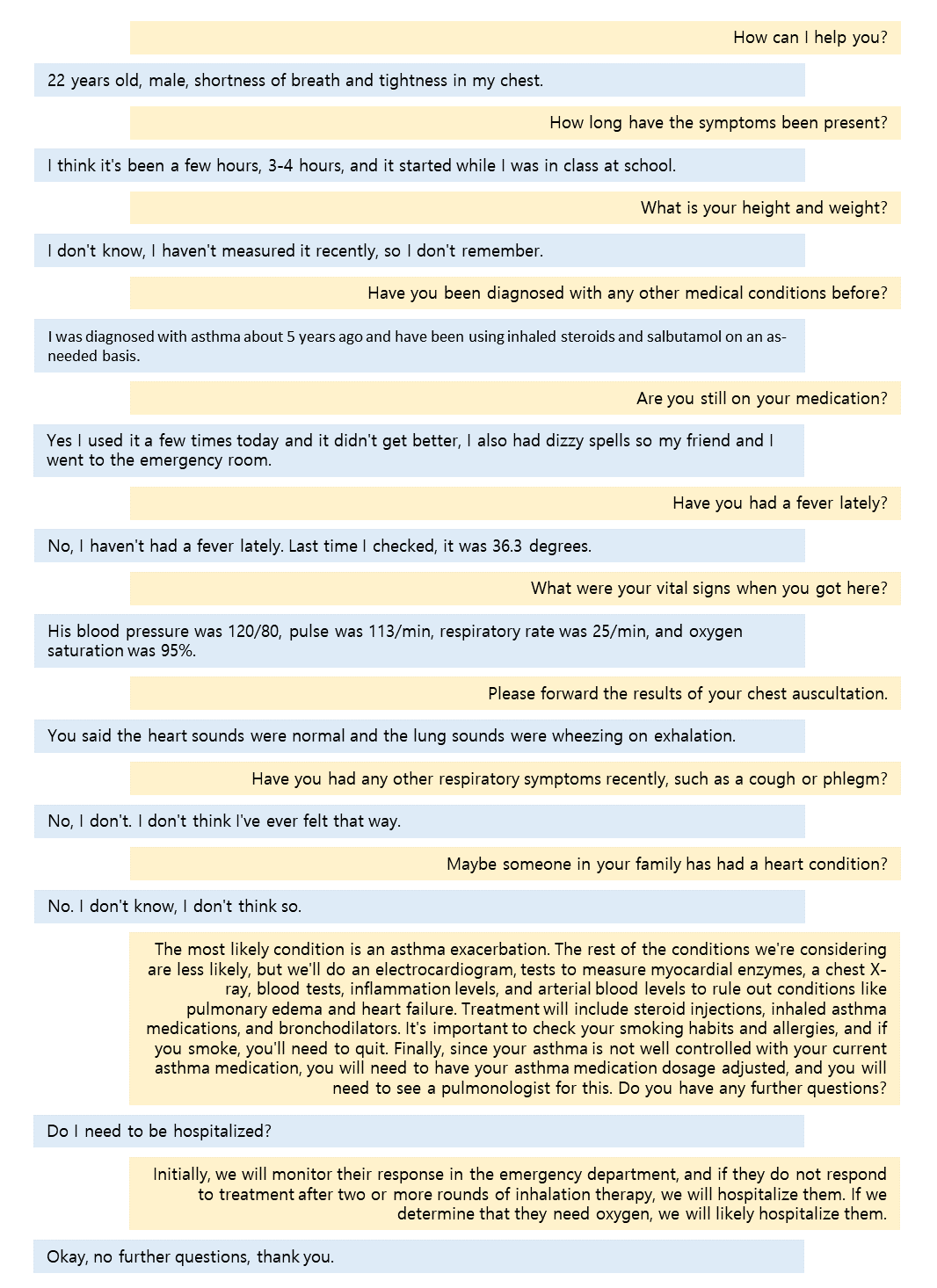 | 1. 1  2. 3  3. 2 | 1. 4  2. 5  3. 4  4. 4  5. 4  6. 5  7. 1 |
| **Chat-GPT** | 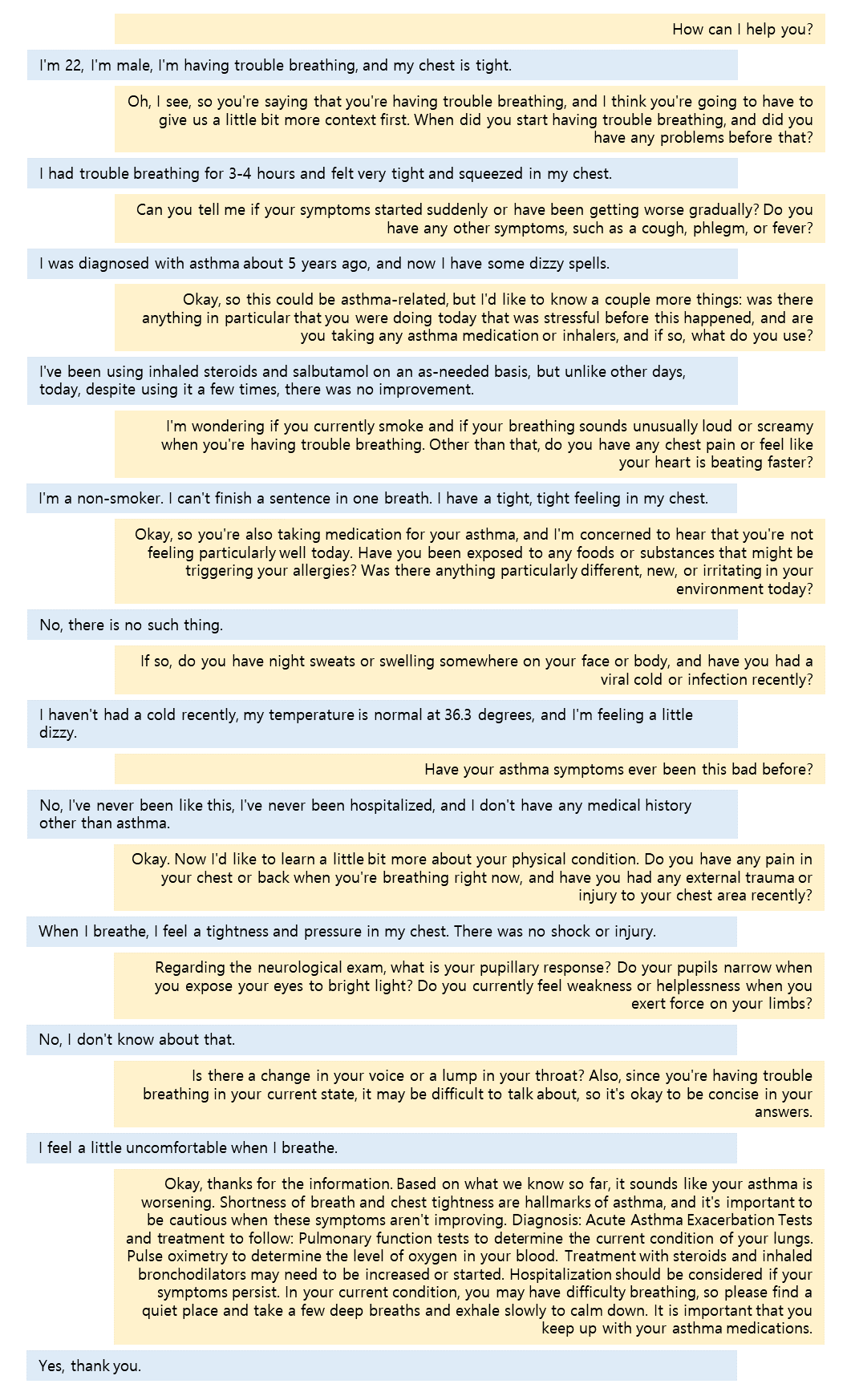 | 1. 3  2. 3  3. 4 | 1. 4  2. 4  3. 5  4. 4  5. 4  6. 5  7. 5 |

**Case 3.** (Reference case 23, with GP)

| **Participants** | **Conversation with virtual patient** | **Score by ER Professor** | **Score by virtual patient** |
| --- | --- | --- | --- |
| **GP** | 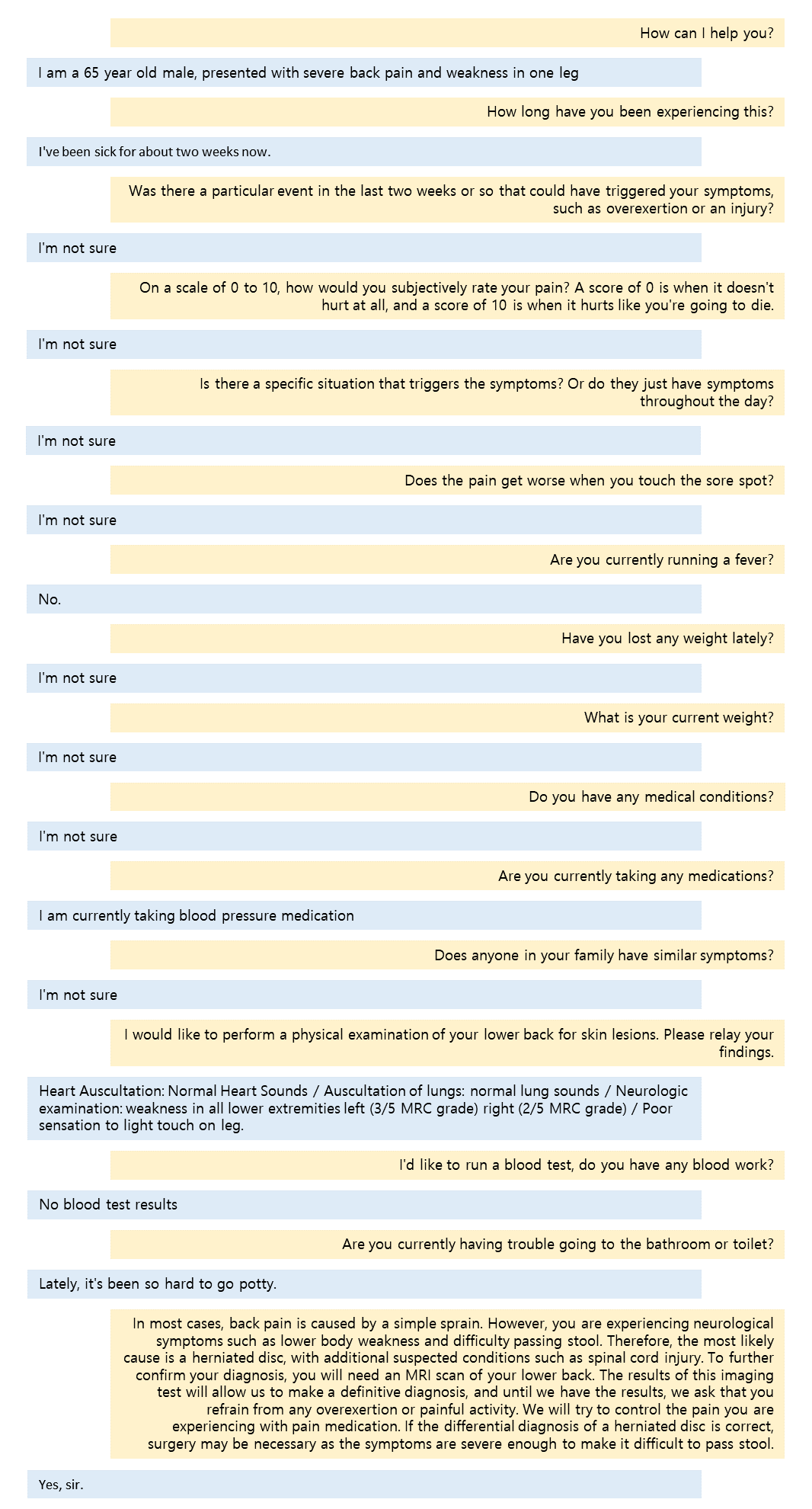 | 1. 2  2. 2  3. 1 | 1. 5  2. 4  3. 4  4. 5  5. 4  6. 4  7. 5 |
| **Chat-GPT** | 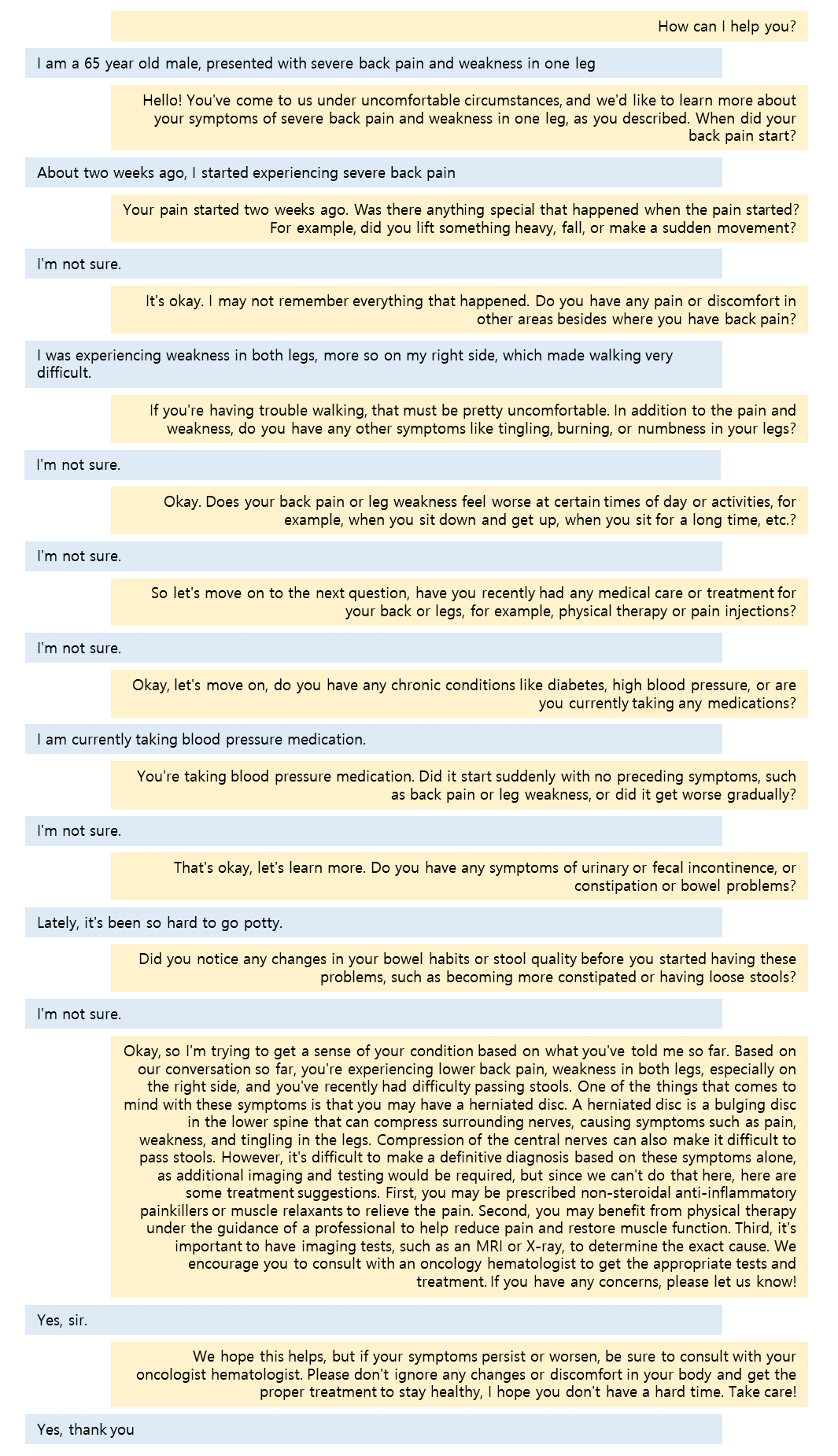 | 1. 3  2. 4  3. 5 | 1. 5  2. 5  3. 5  4. 5  5. 5  6. 5  7. 1 |

**Case 3.** (Reference case 23, with R3)

| **Participants** | **Conversation with virtual patient** | **Score by ER Professor** | **Score by virtual patient** |
| --- | --- | --- | --- |
| **R3** | 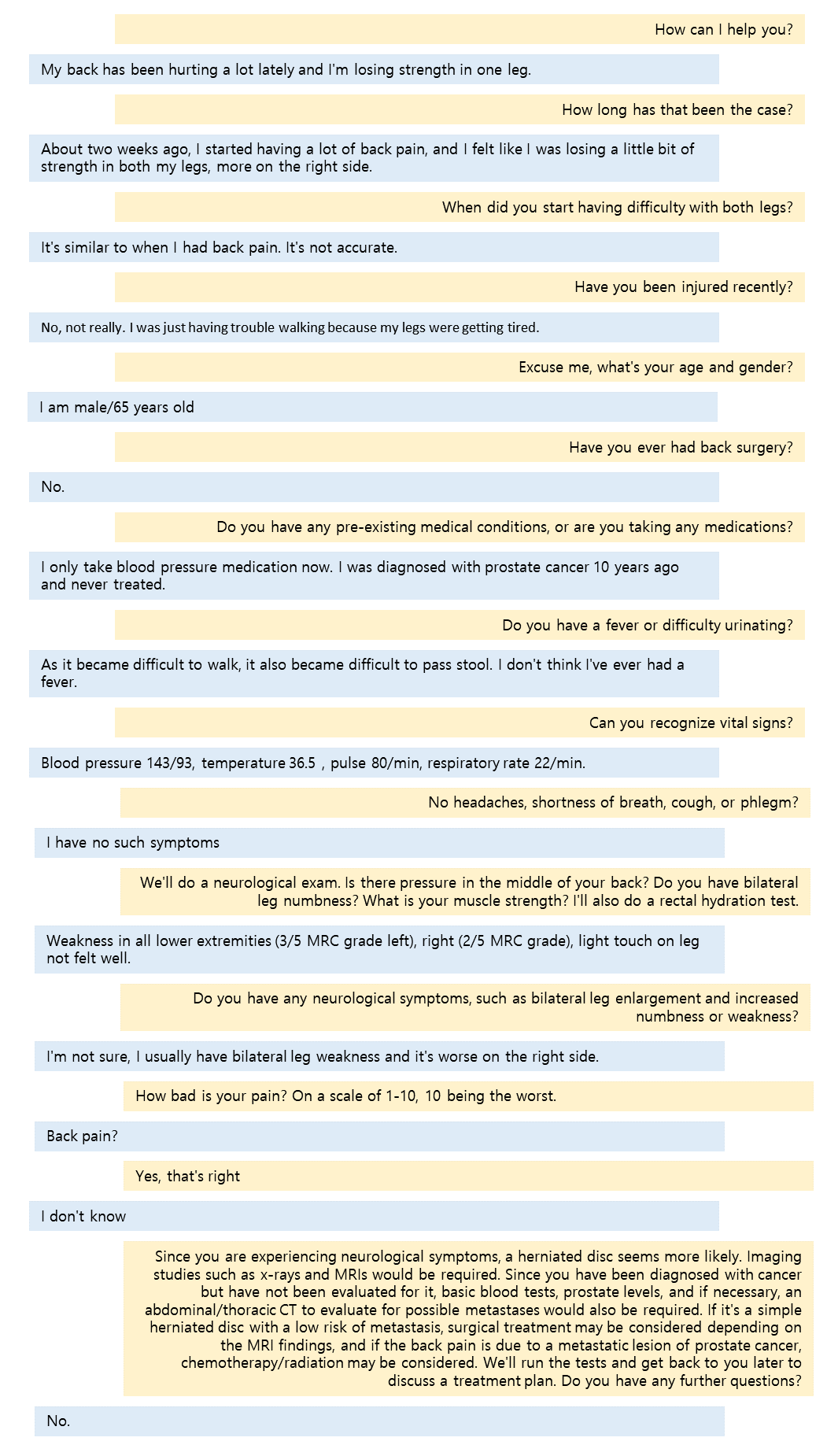 | 1. 3  2. 4  3. 2 | 1. 4  2. 4  3. 4  4. 4  5. 5  6. 4  7. 1 |
| **Chat-GPT** | 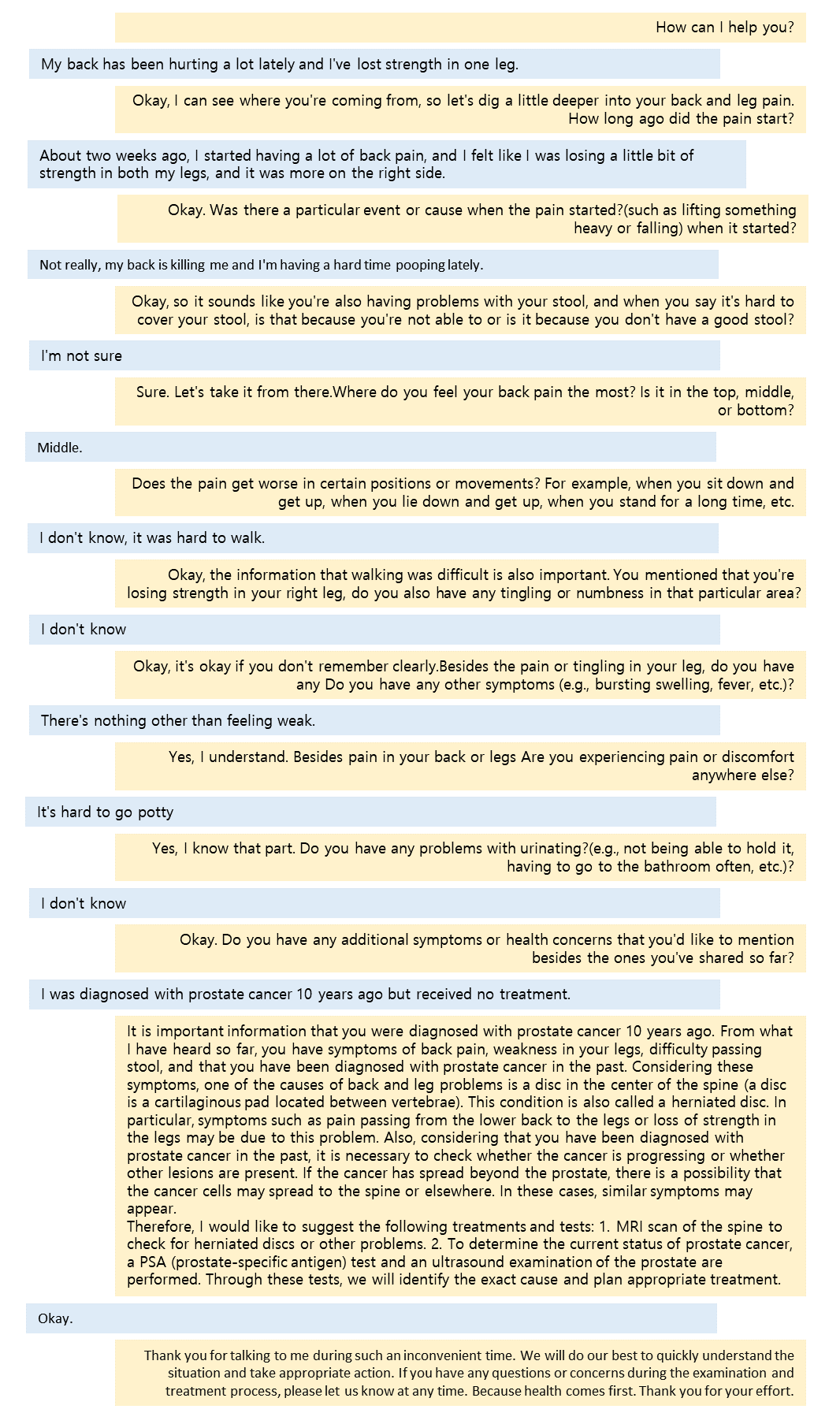 | 1. 4  2. 4  3. 5 | 1. 4  2. 4  3. 3  4. 4  5. 4  6. 3  7. 5 |

**Case 3.** (Reference case 23, with R4)

| **Participants** | **Conversation with virtual patient** | **Score by ER Professor** | **Score by virtual patient** |
| --- | --- | --- | --- |
| **R4** | 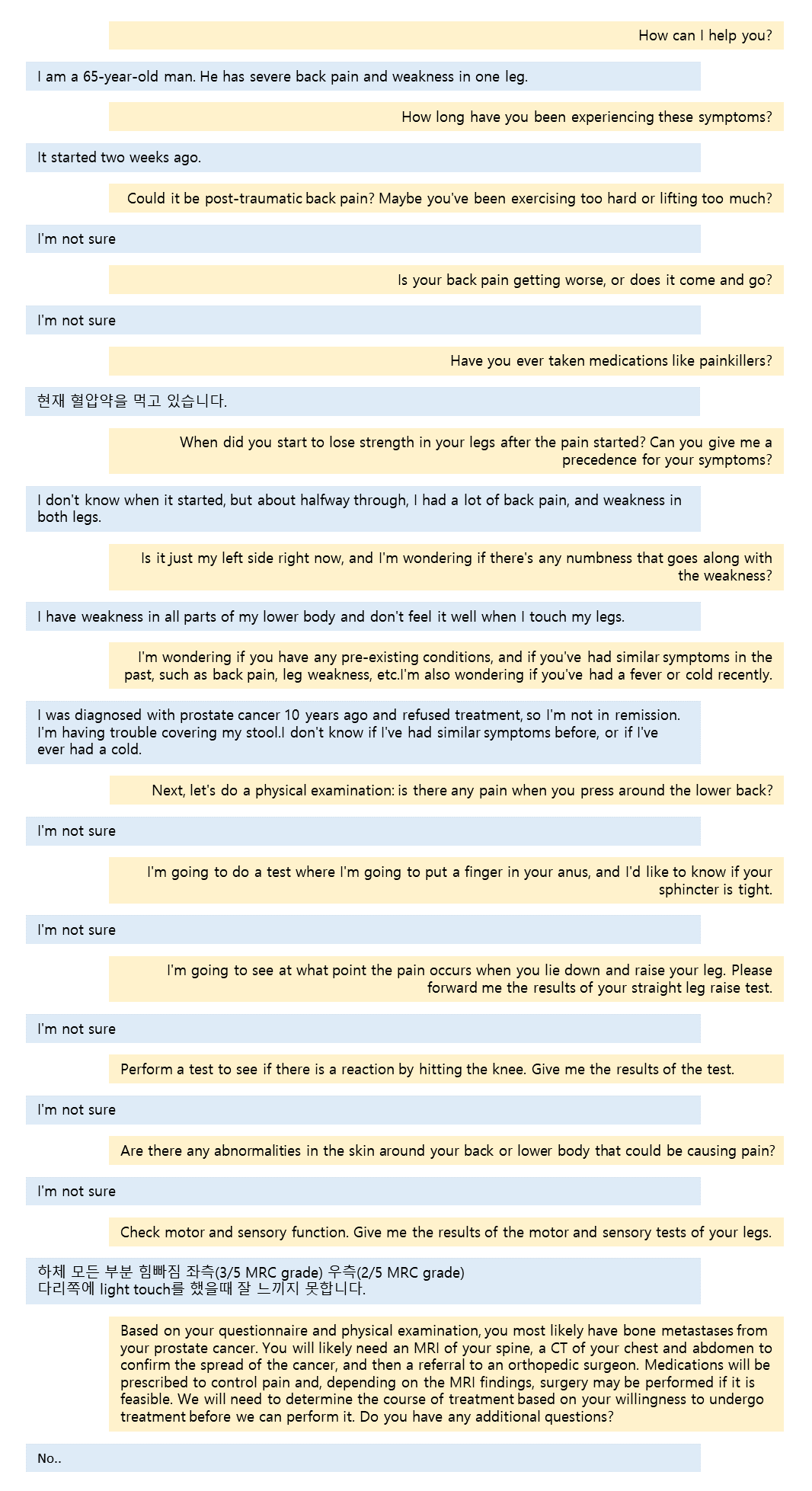 | 1. 4  2. 5  3. 2 | 1. 5  2. 4  3. 4  4. 5  5. 5  6. 4  7. 4 |
| **Chat-GPT** | 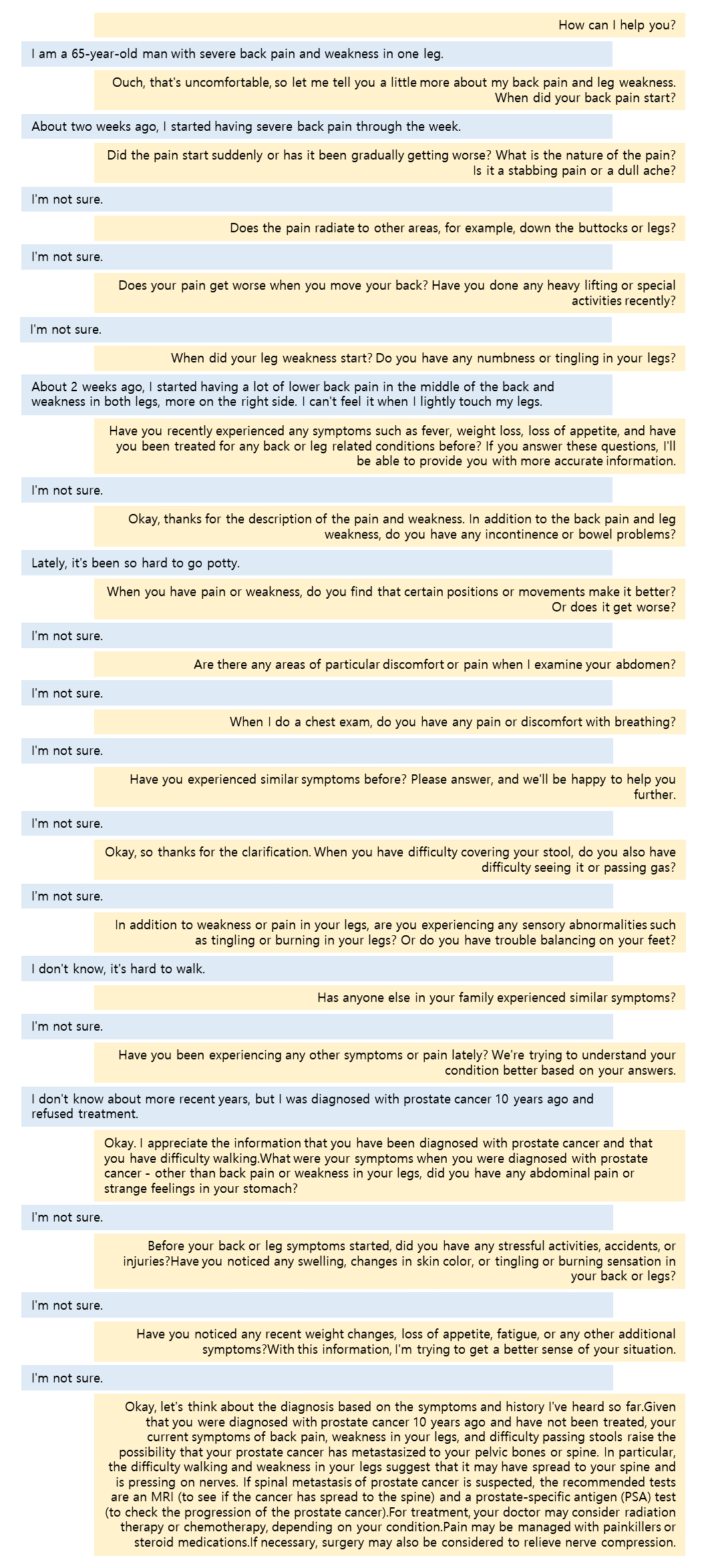 | 1. 4  2. 4  3. 3 | 1. 5  2. 4  3. 4  4. 5  5. 4  6. 4  7. 5 |

**Case 4.** (Reference case 19, with GP)

| **Participants** | **Conversation with virtual patient** | **Score by ER Professor** | **Score by virtual patient** |
| --- | --- | --- | --- |
| **GP** | 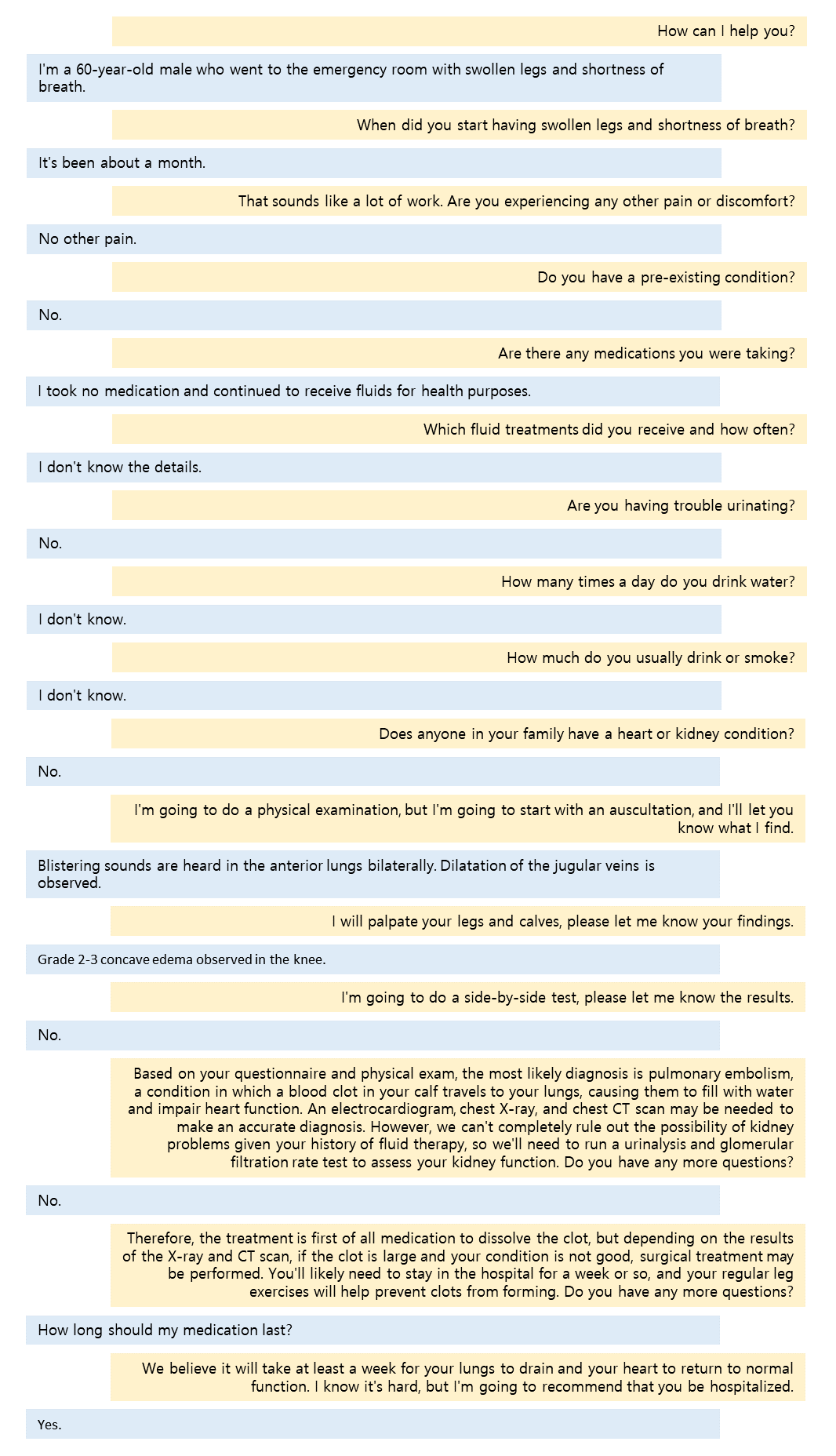 | 1. 3  2. 3  3. 2 | 1. 4  2. 4  3. 4  4. 5  5. 4  6. 1  7. 1 |
| **Chat-GPT** | 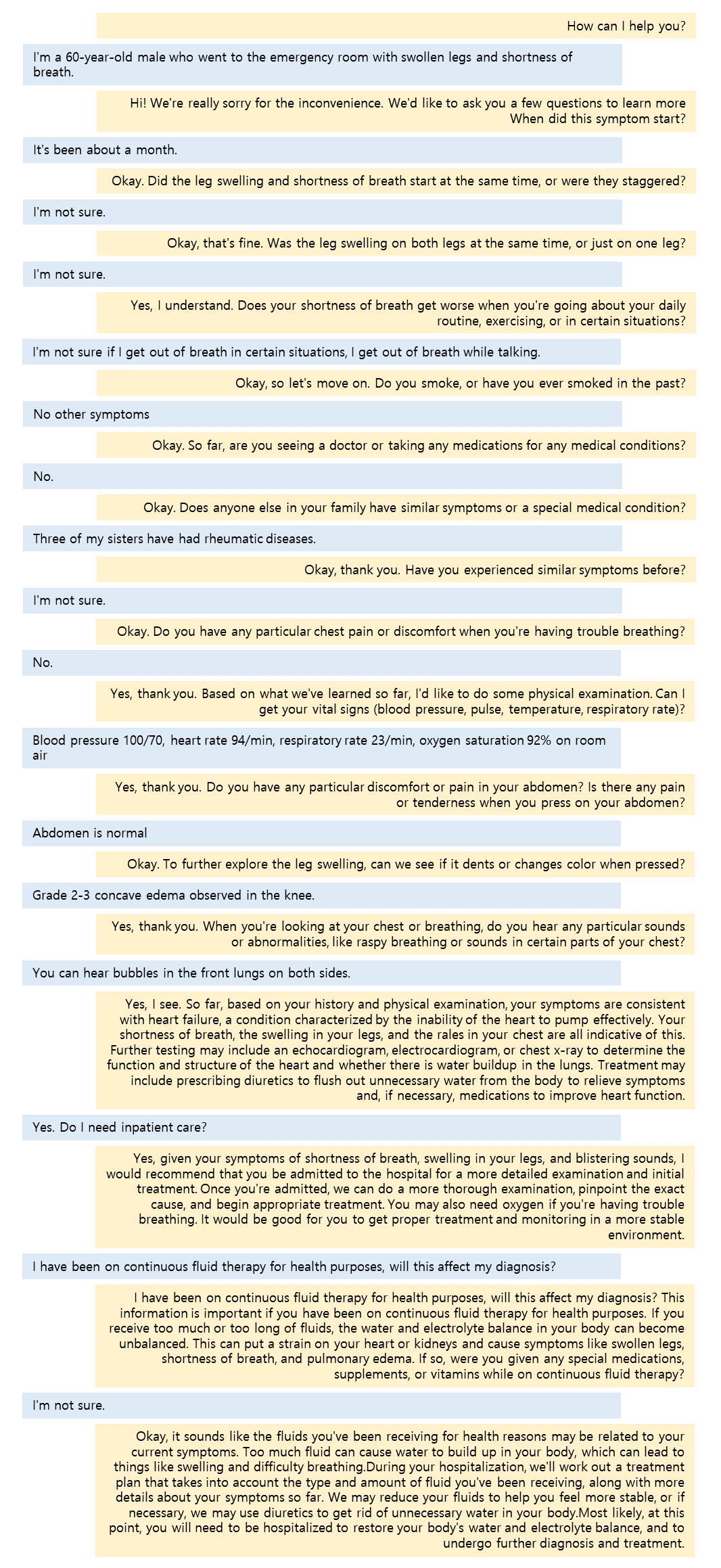 | 1. 3  2. 3  3. 4 | 1. 5  2. 4  3. 4  4. 5  5. 4  6. 4  7. 1 |

**Case 4.** (Reference case 19, with R3)

| **Participants** | **Conversation with virtual patient** | **Score by ER Professor** | **Score by virtual patient** |
| --- | --- | --- | --- |
| **R3** | 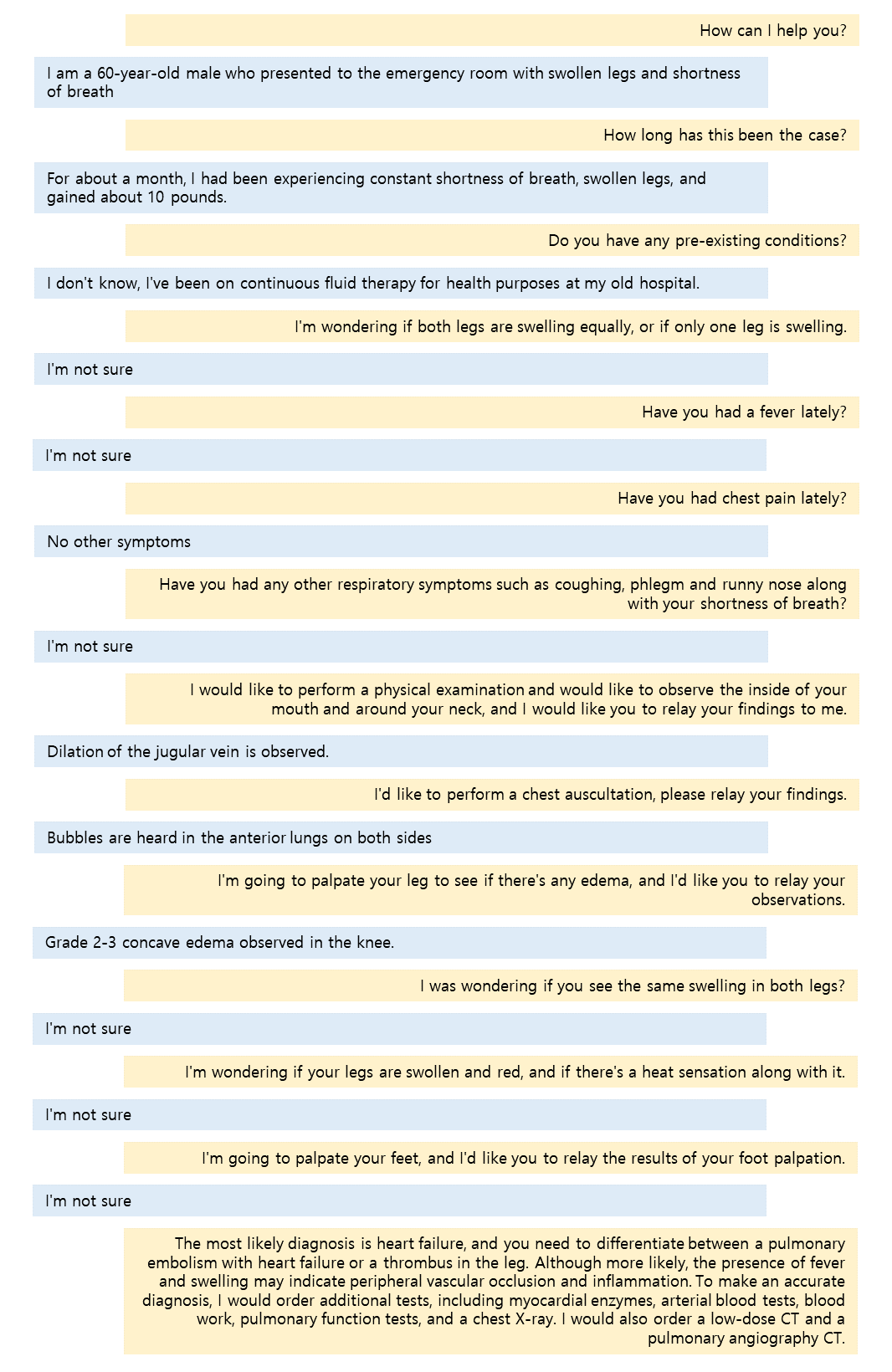 | 1. 3  2. 4  3. 1 | 1. 4  2. 3  3. 2  4. 4  5. 3  6. 3  7. 5 |
| **Chat-GPT** | 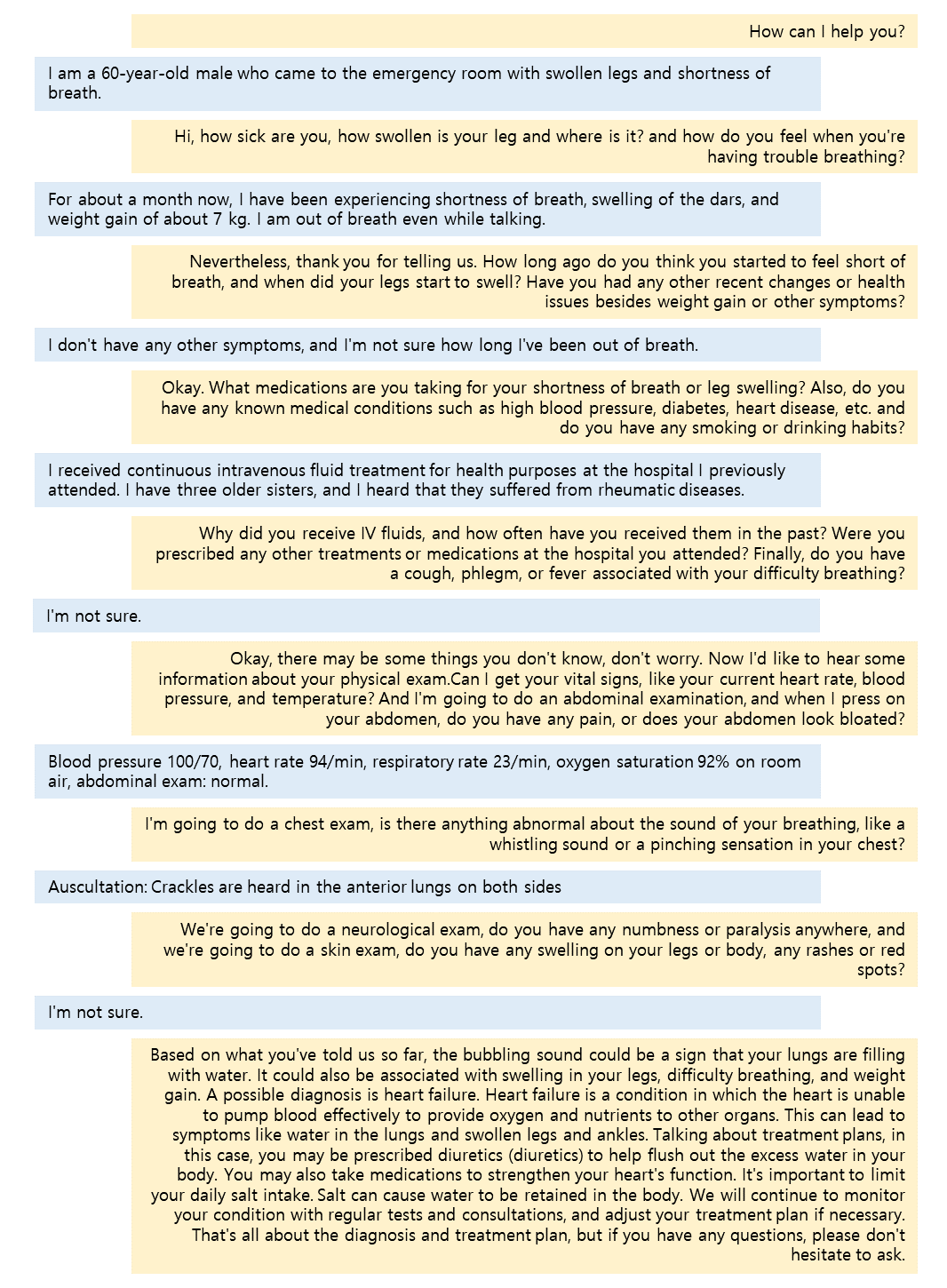 | 1. 4  2. 3  3. 5 | 1. 5  2. 5  3. 5  4. 5  5. 5  6. 5  7. 1 |

**Case 4.** (Reference case 19, with R4)

| **Participants** | **Conversation with virtual patient** | **Score by ER Professor** | **Score by virtual patient** |
| --- | --- | --- | --- |
| **R4** | 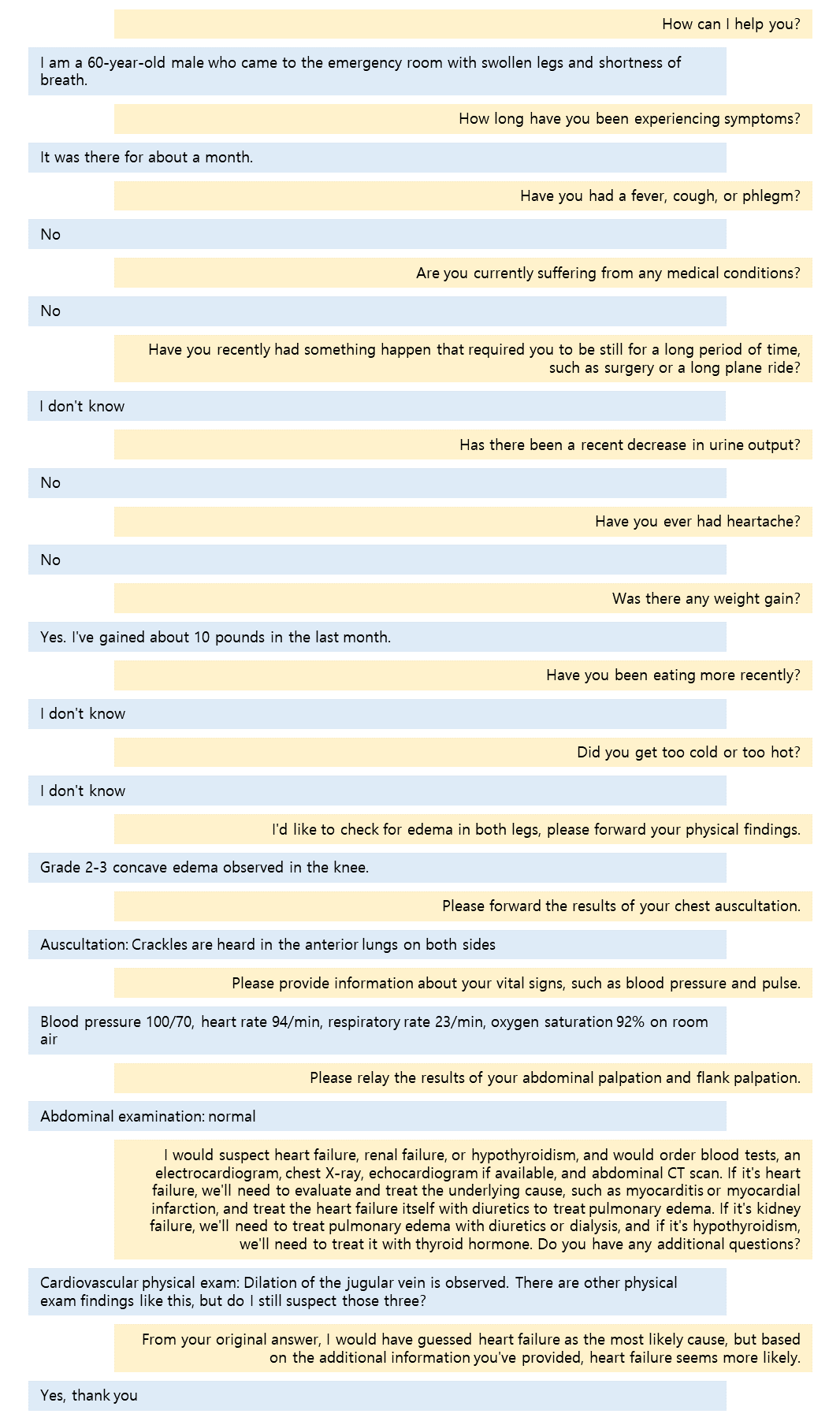 | 1. 3  2. 4  3. 2 | 1. 4  2. 3  3. 2  4. 4  5. 3  6. 3  7. 5 |
| **Chat-GPT** | 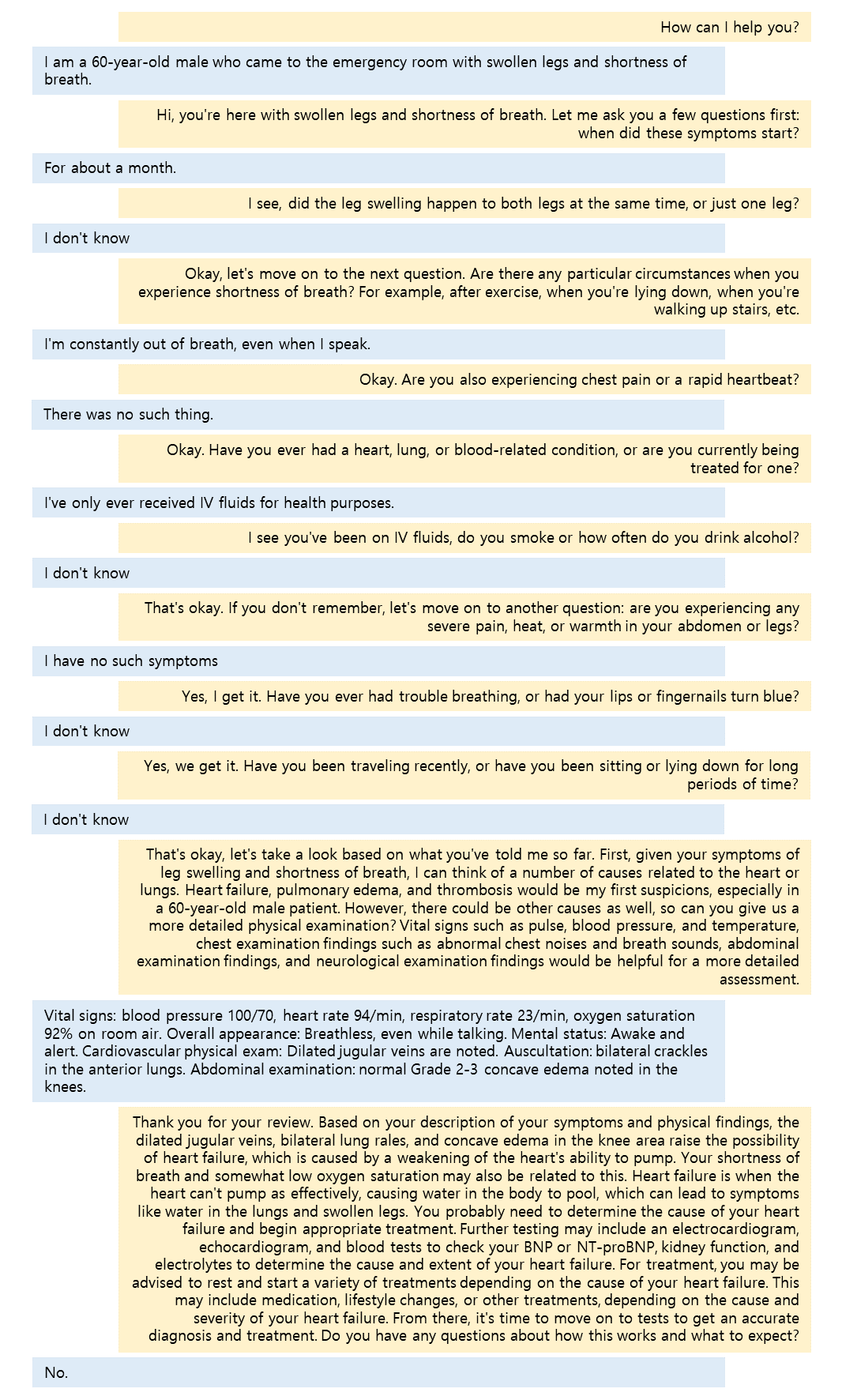 | 1. 4  2. 4  3. 5 | 1. 5  2. 5  3. 5  4. 5  5. 5  6. 5  7. 5 |
