# Supplementary material for: Clinical Performance and Communication Skills of ChatGPT Versus Physicians in Emergency Medicine: Simulated Patient Study
Source: JMIR Med Inform. 2025 Jul 17;13:e68409. doi: 10.2196/68409 (PMC12289221; doi:10.2196/68409)
Supplement: Multimedia Appendix 4 [file medinform-v13-e68409-s004.docx]

**Multimedia Appendix 3:**

**Written examination answer sheets and grading results**

Score by ER Professor

Accuracy scores (6-point Likert scale), Completeness scores (3-point Likert scale)

1. Diagnosis
2. Investigation
3. Management

**Supplementary Figure 2.** Distribution of scores from written examinations

**
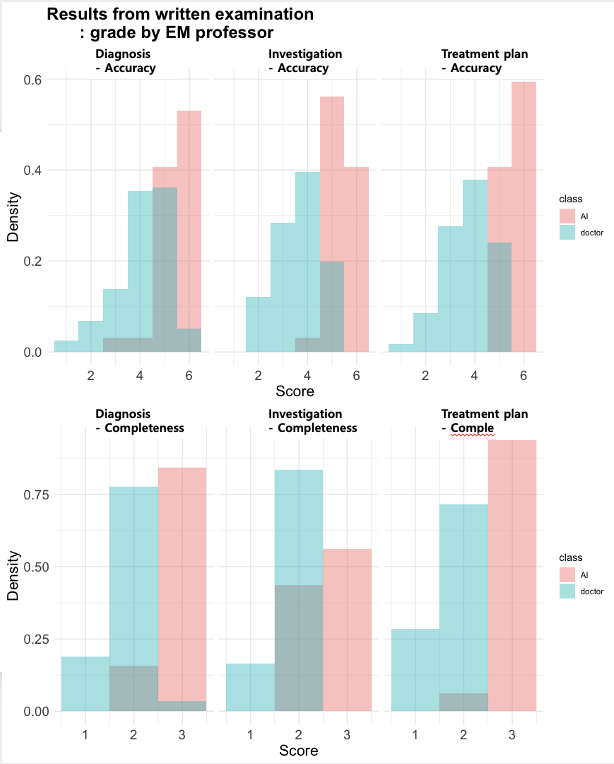
**

**Case 1** (Reference case 5)

| **Participants** | **Generated answer** | **Accuracy scores** | **Completeness scores** |
| --- | --- | --- | --- |
| **GP** | 1. The patient is suffering from meningitis secondary to hayfever  2. Cerebrospinal fluid analysis (spinal tap), Blood culture, WBC, ESR, CRP analyses should be made to confirm infection and systemic inflammation  3. Empiric antibiotics (3^rd^ order cephalosporins and aminoglycosides) should be administered considering the limited etiology of meningitis. Antipyretics for fever control is also necessary | 1. 4  2. 4  3. 5 | 1. 2  2. 2  3. 2 |
| **R3** | 1. Sepsis, complication; meningitis. Or Dx; leukemia, complication; intracranial hemorrhage  2. blood test involving cbc, routine chemistry, coagulation test, inflammatory marker. Brain non-contrast CT. If not contraindicated, CSF study.  3. Empiric antibiotics | 1. 5  2. 5  3. 4 | 1. 2  2. 2  3. 1 |
| **R4** | 1. bacterial meningitis  2. nonenhance brain ct scan, CSF tapping  3. antipyretics, antibiotics, IV hydration | 1. 4  2. 4  3. 5 | 1. 1  2. 2  3. 1 |
| **Chat-GPT** | Based on the provided history and examination findings, the primary diagnosis for this 20-year-old student is likely bacterial meningitis with a complication of disseminated intravascular coagulation (DIC).   1. Diagnosis and Complication:    - Bacterial Meningitis:      - Supporting Signs: Fever, headache, neck stiffness (suggesting meningeal irritation), altered mental status (GCS of 12/15), and a purpuric rash.      - Age Group and Context: Meningitis is a concern in young adults, especially in settings like universities where close contact facilitates the spread of infectious agents.    - Disseminated Intravascular Coagulation (DIC):      - Indications: Purpuric rash and bleeding gums. DIC is a secondary complication characterized by systemic activation of blood clotting, which leads to the formation of blood clots in small vessels and can result in multiple organ damage. It also consumes clotting factors and platelets, leading to a risk of severe bleeding. 2. Immediate Investigations:    - Lumbar Puncture: To obtain cerebrospinal fluid (CSF) for analysis (cell count, glucose, protein, Gram stain, culture, and possibly PCR for bacterial DNA).    - Blood Tests: Complete blood count (CBC), blood cultures, coagulation profile (including INR, APTT, fibrinogen levels), electrolytes, renal function tests, and C-reactive protein or erythrocyte sedimentation rate.    - Imaging: CT scan of the head before lumbar puncture if there are signs of raised intracranial pressure or focal neurological signs (to rule out mass effect). 3. Empiric Treatment in Emergency Department:    - Antibiotics: Immediate empirical antibiotic therapy is crucial. A combination of ceftriaxone (or cefotaxime) plus vancomycin is typically used to cover the most likely pathogens (Neisseria meningitidis, Streptococcus pneumoniae).    - Steroids: Dexamethasone to reduce the inflammatory response in bacterial meningitis.    - Supportive Care: Fluid resuscitation (carefully monitored, as overhydration can worsen cerebral edema), antipyretics for fever, and maintenance of adequate oxygenation.    - Management of DIC: Treatment is mainly supportive. Fresh frozen plasma, platelets, or cryoprecipitate can be used to correct severe coagulopathies, especially if there's active bleeding.   This condition is a medical emergency, and rapid initiation of treatment is essential to reduce the risk of mortality and serious complications. | 1. 5  2. 6  3. 6 | 1. 3  2. 3  3. 3 |

**Case 2** (Reference case 18)

| **Participants** | **Generated answer** | **Accuracy scores** | **Completeness scores** |
| --- | --- | --- | --- |
| **GP** | 1. The most probable diagnosis is sepsis due to acute urinary tract infection, caused by chronic immunosuppression due to Type 2 diabetes  2. Blood Culture, WBC, ESR, CRP, Urinalysis, Urine culture  3. Fluids should be administered, epinephrine may be considered for low blood pressure, antipyretics for fever control, and antibiotics (cephalosporins such as cefotaxime or ceftriaxone considering that the most likely etiology is urinary tract infection) | 1. 5  2. 5  3. 3 | 1. 2  2. 2  3. 2 |
| **R3** | 1. Septic shock due to urinary track infection  2. urine analysis, blood test, Culture (urinary, blood), x-ray, CT if needed.  3. Fluid resuscitation, IV empiric antibioitics, related symptom control (fever) | 1. 5  2. 5  3. 5 | 1. 2  2. 2  3. 2 |
| **R4** | 1. septic shock due to urinary tract infection  2. blood test(cbc, routine chemistry, inflammatory marker, lactic acid), blood culture, urine analysis, CT enhance scan if needed  3. IV hydration, broad coverage antibiotics, vasopressor if neeeded | 1. 5  2. 5  3. 5 | 1. 2  2. 2  3. 2 |
| **Chat-GPT** | The presentation of this 70-year-old woman suggests a diagnosis of severe urinary tract infection (UTI), likely progressing to urosepsis, given her systemic symptoms and vital signs.   1. Diagnosis and Likely Underlying Cause:    - Severe Urinary Tract Infection/Urosepsis:      - Supporting Evidence: History of dysuria, urinary frequency, fever, hypotension (blood pressure 80/40), tachycardia, and altered mental status (AMT score of 5/10). These signs and symptoms indicate a systemic response to infection.      - Risk Factors: History of type 2 diabetes and frequent UTIs, and residence in a care home setting where infections can be more common.    - Underlying Cause: The UTI, likely bacterial in nature, has potentially progressed to a more severe state, causing systemic inflammatory response syndrome (SIRS) and sepsis. 2. Next Steps in Investigation:    - Urinalysis and Urine Culture: To confirm UTI and identify causative organism.    - Blood Cultures: To check for bacteremia, a component of urosepsis.    - Complete Blood Count (CBC): Looking for leukocytosis or other signs of infection.    - Serum Biochemistry: Including renal function tests and electrolytes, as UTIs can impact kidney function, and sepsis can lead to metabolic derangements.    - Lactic Acid: Elevated levels can indicate poor tissue perfusion, a common feature in sepsis.    - Imaging: Ultrasound or CT scan of the abdomen and pelvis if there is suspicion of an obstructive process or complicated UTI. 3. Management of the Patient:    - Fluid Resuscitation: Aggressive fluid therapy to counteract the hypotension and potential septic shock.    - Empirical Antibiotic Therapy: Broad-spectrum antibiotics should be initiated as soon as possible, and then tailored based on culture results.    - Monitoring and Supportive Care: Close monitoring in a high-dependency or intensive care unit for vital signs, urine output, and mental status. Supportive care for any organ dysfunction.    - Blood Sugar Control: Management of her diabetes is important, as hyperglycemia can worsen infection outcomes.    - Review Medications: Especially metformin, as it may need to be held if there is any renal impairment or lactic acidosis.    - Further Assessment and Treatment: Depending on the response to initial treatment and findings from further investigations, additional management steps may be necessary.   This patient's condition is serious, with potential for rapid deterioration, necessitating prompt and aggressive management. Regular re-evaluation and adjustment of treatment based on her clinical response and investigation results are crucial. | 1. 6  2. 5  3. 6 | 1. 3  2. 3  3. 3 |

**Case 3** (Reference case 41)

| **Participants** | **Generated answer** | **Accuracy scores** | **Completeness scores** |
| --- | --- | --- | --- |
| **GP** | 1. The diagnosis is intra-cranial hemorrhage (ICH) due to traumatic brain injury (TBI)  2. Brain images should be taken, and considering the urgency of the situation, computed tomography (CT) should be prioritized over magnetic resonance imaging (MRI).  3. The patient should be hospitalized and routinely evaluated by brain CT. | 1. 3  2. 4  3. 2 | 1. 1  2. 2  3. 1 |
| **R3** | 1. Intracranial hemorrhage, DDx; spinal cord injury  2. Brain CT, cervical spine CT  3. absoulte bed rest, Neck collar apply, control blood pressure into target range in case of hemorrhage | 1. 4  2. 4  3. 5 | 1. 2  2. 1  3. 2 |
| **R4** | 1. ischemic stroke  2. brain ct scan  3. IV hydration, tPA, blood pressure management | 1. 5  2. 4  3. 4 | 1. 2  2. 1  3. 2 |
| **Chat-GPT** | The clinical presentation of this 63-year-old man suggests a cerebrovascular accident (CVA), more specifically, a stroke.   1. Diagnosis:    - Stroke:      - Supporting Evidence: Acute onset of neurologic deficits including slurred speech, inability to support himself, limb weakness (right arm appearing limp, diminished sensation and reflexes on the right side), and shaking in the left arm. His risk factors include hypertension, hyperlipidemia, and smoking.      - Type of Stroke: The symptoms suggest an ischemic stroke; however, a hemorrhagic stroke cannot be ruled out without imaging. 2. Investigations to Prioritize:    - Brain Imaging:      - Non-contrast CT Scan of the Head: The first-line imaging to distinguish between ischemic and hemorrhagic stroke. It's crucial to perform this quickly as the management differs significantly between these two types.    - Blood Tests:      - Complete Blood Count (CBC): To check for anemia or infection.      - Electrolytes, Renal Function, and Glucose Levels: Electrolyte imbalances or hyperglycemia can mimic stroke symptoms.      - Coagulation Profile: Especially if anticoagulant therapy is considered.    - ECG (Electrocardiogram):      - To rule out cardiac sources of emboli, such as atrial fibrillation, which is a common cause of ischemic strokes.    - Carotid Ultrasound:      - To assess for significant carotid stenosis, which could be a source of embolic stroke. 3. Management:    - Acute Management:      - Ischemic Stroke: If confirmed and within the therapeutic window (typically within 4.5 hours of symptom onset), intravenous thrombolysis with recombinant tissue plasminogen activator (rtPA) may be considered. Endovascular thrombectomy might also be an option in certain cases.      - Hemorrhagic Stroke: Management focuses on controlling blood pressure, reversing anticoagulation if present, and neurosurgical interventions in certain cases.    - Supportive Care:      - Blood Pressure Management: Careful control, especially in ischemic stroke. However, overly aggressive lowering in the acute phase should be avoided.      - Airway and Breathing Support: If necessary.      - Prevention of Complications: Such as deep vein thrombosis, aspiration pneumonia, and pressure sores.    - Secondary Prevention:      - Risk Factor Management: Addressing hypertension, hyperlipidemia, and smoking cessation.      - Anticoagulation/Antiplatelet Therapy: Depending on the type of stroke and the underlying cause.   The timing of intervention in stroke is critical for optimal outcomes, emphasizing the importance of rapid assessment and initiation of appropriate therapy. | 1. 5  2. 6  3. 5 | 1. 3  2. 3  3. 3 |

**Case 4** (Reference case 60)

| **Participants** | **Generated answer** | **Accuracy scores** | **Completeness scores** |
| --- | --- | --- | --- |
| **GP** | 1. The most likely diagnosis is bowel perforation due to peptic ulcer disease.  2. Endoscopic evaluation is contraindicated considering the likeliness of bowel perforation, and therefore an abdomen/chest X-ray to examine the presence of pneumoperitoneum is the most adequate investigation.  3. The patient should be referred to the general surgery department to undergo exploratory surgery as soon as possible. | 1. 3  2. 3  3. 4 | 1. 2  2. 2  3. 1 |
| **R3** | 1. DDx; pancreatitis, ileus due to mechanical obstruction, GI tract perforation. Gastric perforation is the most likely diagnosis.  2. chest and abdomen x-ray, abdomen CT in needed. Blood test  3. pain control, levin-tube insertion and drainage, empiric antibiotics, | 1. 6  2. 5  3. 5 | 1. 3  2. 2  3. 2 |
| **R4** | 1. panperitonitis due to gastric ulcer perforation most likely, Differential diagnosis - alcoholic gastritis, pancreatitis  2. chest AP xray, abdominal ct scan with enhancement  3. IV hydration, analgesics, nasogastric tube drainage, emergency operation | 1. 5  2. 5  3. 5 | 1. 2  2. 2  3. 2 |
| **Chat-GPT** | 1. Differential Diagnosis and Most Likely Diagnosis:    - Perforated Peptic Ulcer:      - Supporting Evidence: Severe epigastric pain, history of gastroesophageal reflux disease (GERD) and long-term use of omeprazole (which might mask ulcer symptoms), regular ibuprofen use (a risk factor for peptic ulcer), abdominal rigidity, and voluntary guarding. Smoking and alcohol use are additional risk factors.      - Other Considerations: Acute pancreatitis, biliary colic, or cholecystitis, but the acute onset with guarding and distension suggests perforation is more likely.    - Most Likely Diagnosis: Given the severity of the pain, the rigid abdomen, and the patient's position (indicative of peritoneal irritation), a perforated peptic ulcer is highly likely. 2. Investigations in the ED:    - Complete Blood Count (CBC): To look for signs of infection or anemia.    - Serum Electrolytes, Renal and Liver Function Tests: To assess overall organ function.    - Amylase and Lipase: To rule out pancreatitis.    - Abdominal X-ray: Upright or lateral decubitus view to look for free air under the diaphragm, which would suggest a perforation.    - CT Scan of the Abdomen and Pelvis with Contrast: For detailed evaluation of the abdominal organs and to confirm the site and extent of any perforation.    - Blood Cultures and Lactic Acid: If sepsis is suspected. 3. Management in the ED:    - Resuscitation: Initiate intravenous fluids to manage hypotension and tachycardia.    - Pain Management: Administer appropriate analgesics.    - Antibiotic Therapy: Broad-spectrum antibiotics to cover potential peritoneal contamination.    - Nasogastric Tube and Urinary Catheter: For decompression and monitoring output, respectively.    - Oxygen Supplementation: To maintain adequate oxygen saturation.    - Urgent Surgical Consultation: Perforated peptic ulcer is a surgical emergency.    - Monitor Vital Signs and Labs Closely: For signs of deterioration or sepsis.   The patient's presentation is suggestive of an acute surgical abdomen, likely due to a perforated peptic ulcer, necessitating prompt surgical intervention after initial stabilization. | 1. 6  2. 5  3. 6 | 1. 3  2. 3  3. 3 |

**Case 5** (Reference case 64)

| **Participants** | **Generated answer** | **Accuracy scores** | **Completeness scores** |
| --- | --- | --- | --- |
| **GP** | 1. The most likely diagnosis is unstable angina, considering the pain region and no guarding or rebound tenderness, presence of radiating pain, and constant pain which separates it from stable angina. The most important differential diagnosis is myocardial infarction.  2. An Electrocardiogram (EKG) is the exam of choice to diagnose unstable angina. Cardiac enzymes such as Troponin I are also necessary.  3. Nitroglycerin and Aspirin are the most adequate drugs for acute management of anginal pain. | 1. 2  2. 2  3. 2 | 1. 1  2. 1  3. 1 |
| **R3** | 1. Cholecystitis, cholangitis, diverticulitis, gastritis. Cholecystitis is the most likely diagnosis.  2. Abdomen ultrasound or CT  3. Pain control | 1. 5  2. 5  3. 3 | 1. 3  2. 2  3. 1 |
| **R4** | 1. cholecystitis most likely, Differential diagnosis - cholelithiasis, gastroenteritis  2. Abdominal US, abdominal ct scan with enhance if needed  3. analgesics, iv hydration | 1. 5  2. 5  3. 4 | 1. 2  2. 2  3. 1 |
| **Chat-GPT** | The clinical presentation of this 55-year-old woman suggests a few differential diagnoses, with the most likely being cholecystitis, possibly acute cholecystitis.   1. Differential Diagnosis and Most Likely Diagnosis:    - Cholecystitis, Likely Acute Cholecystitis:      - Supporting Evidence: Constant right upper quadrant and epigastric pain radiating to the back, nausea, vomiting, and tachycardia. The location and nature of the pain are typical for gallbladder pathology.    - Other Considerations:      - Biliary colic: Similar presentation, but pain is usually more colicky than constant.      - Peptic ulcer disease: Can cause epigastric pain but less likely to localize to the right upper quadrant.      - Pancreatitis: Usually presents with more diffuse abdominal pain, often radiating to the back.      - Hepatic pathology: Such as hepatitis or hepatic abscess, but less likely given the presentation. 2. Investigations in the Emergency Department:    - Blood Tests:      - Complete Blood Count (CBC): To assess for leukocytosis, indicative of infection or inflammation.      - Liver Function Tests (LFTs) and Serum Lipase: To evaluate liver and gallbladder function and to rule out pancreatitis.      - Serum Electrolytes: To assess overall metabolic status.    - Abdominal Ultrasound: The first-line imaging modality to evaluate the gallbladder for stones, wall thickening, and pericholecystic fluid, which are suggestive of cholecystitis.    - CT Scan of the Abdomen and Pelvis: If the ultrasound is inconclusive or to assess for complications and alternative diagnoses. 3. Acute Management:    - Pain Management: Adequate analgesia, typically with opioids given the severity of the pain.    - Anti-emetics: To control nausea and vomiting.    - Intravenous Fluids: To maintain hydration, especially if the patient has been vomiting.    - Antibiotics: If acute cholecystitis is suspected, as bacterial infection is often a component.    - NPO (Nothing by Mouth): To rest the gallbladder.    - Surgical Consultation: For potential cholecystectomy, especially if acute cholecystitis is confirmed.    - Monitoring: Vital signs and abdominal examination should be closely monitored for any signs of deterioration, which could indicate complications like gallbladder rupture or progression to more severe illness.   Early and appropriate management of cholecystitis is essential to prevent complications, and surgical intervention may be necessary depending on the severity and duration of the condition. | 1. 6  2. 5  3. 6 | 1. 3  2. 3  3. 3 |

**Case 6** (Reference case 83)

| **Participants** | **Generated answer** | **Accuracy scores** | **Completeness scores** |
| --- | --- | --- | --- |
| **GP** | 1. The diagnosis is acute exacerbation of asthma, considering the increased need for medications and poor control of asthma symptoms despite the increased use of drugs. Notable differential diagnoses are Chronic obstructive pulmonary disease (COPD) or pneumonia.  2. No further evaluations are needed. The diagnosis of asthma acute exacerbation can be made solely on the basis of clinical presentation.  3. The patient is currently on inhaled corticosteroids and short acting beta agonist for the control of asthma. A step up of drug use should be considered, such as the addition of a long-acting beta agonist or a leukotriene antagonist. | 1. 4  2. 3  3. 4 | 1. 2  2. 2  3. 2 |
| **R3** | 1. asthma acute exacerbation  2. chest x-ray  3. salbutamol, Ipratropium nebulizer, systemic steroid | 1. 6  2. 5  3. 5 | 1. 3  2. 2  3. 2 |
| **R4** | 1. asthma acute exarcabation  2. chest plain x-ray, arterial blood gas analysis if needed  3. beta agonist/steroid nebulizer, IV steroid, O2 supply | 1. 6  2. 5  3. 5 | 1. 2  2. 2  3. 2 |
| **Chat-GPT** | The clinical presentation of this 10-year-old child indicates a diagnosis of an acute asthma exacerbation.   1. Diagnosis:    - Acute Asthma Exacerbation:      - Supporting Evidence: History of worsening cough and shortness of breath, limited response to salbutamol (a short-acting beta-agonist), a known history of asthma, increased requirement of rescue inhaler in recent days, and auscultatory findings of bilateral wheezing with reduced air entry. The strong family history of atopy further supports this diagnosis. 2. Further Investigations in the Emergency Department:    - Pulse Oximetry: Continuous monitoring of oxygen saturation.    - Peak Expiratory Flow Rate (PEFR): Measurement when the child is able, to assess the severity of the asthma exacerbation and response to treatment.    - Blood Tests: Not routinely required but can include a Complete Blood Count (CBC) and arterial blood gases (ABG) in severe cases to assess for hypoxemia and respiratory acidosis.    - Chest X-ray: If there is suspicion of a complicating factor, such as pneumonia or pneumothorax.    - Spirometry: Post-acute treatment, to assess lung function. 3. Management of the Child:    - Bronchodilators:      - Short-Acting Beta-Agonists (SABA): Repeated doses of salbutamol via a nebulizer or spacer.    - Corticosteroids:      - Systemic corticosteroids (e.g., oral prednisolone) to reduce airway inflammation.    - Oxygen Therapy:      - Supplemental oxygen to maintain SpO2 > 94%.    - Monitoring:      - Close monitoring of respiratory rate, oxygen saturation, heart rate, and level of distress.    - Rehydration:      - Oral or intravenous fluids, especially if there is dehydration due to increased respiratory effort and decreased oral intake.    - Education:      - Asthma education for the patient and caregivers, including proper inhaler technique, trigger avoidance, and recognizing signs of exacerbation.    - Follow-Up and Review:      - Regular follow-up with a pediatrician or asthma specialist to optimize long-term asthma management.    - Consideration of Other Treatments:      - In severe cases or those not responding to initial treatment, other therapies like ipratropium bromide, magnesium sulfate, or intravenous bronchodilators may be considered.   Acute asthma exacerbations require prompt and aggressive treatment to relieve airway obstruction and inflammation and to prevent further respiratory compromise. The long-term goal is to achieve good asthma control with appropriate maintenance therapy and education. | 1. 6  2. 5  3. 6 | 1. 3  2. 3  3. 3 |

**Case 7** (Reference case 95)

| **Participants** | **Generated answer** | **Accuracy scores** | **Completeness scores** |
| --- | --- | --- | --- |
| **GP** | 1. The diagnosis is the pre-eclampsia, considering the presence of hypertension and nephrotic syndrome in a pregnant patient.  2. Uterus ultrasonography for the evaluation of adequacy for caesarean section.  The use of antihypertensives for blood pressure control is necessary, and a caesarean section is deemed necessary. | 1. 5  2. 2  3. 4 | 1. 2  2. 1  3. 2 |
| **R3** | 1. Eclampsia  2. NST test for fetus, blood test  3. magnesium sulfate, blood pressure control | 1. 2  2. 3  3. 5 | 1. 2  2. 2  3. 2 |
| **R4** | 1. preeclampsia  2. blood test(cbc, routine chemistry, electrolyte), fetal heart rate check  3. blood pressure control(ex, IV hydralazine, labetolol), IV magnesium | 1. 5  2. 3  3. 5 | 1. 2  2. 1  3. 2 |
| **Chat-GPT** | The clinical presentation and findings of this 35-year-old pregnant woman suggest a diagnosis of preeclampsia.   1. Diagnosis:    - Preeclampsia:      - Supporting Evidence: The combination of new-onset hypertension (blood pressure 170/101 mmHg), proteinuria (3+ on urinalysis), headache, visual disturbances (flashing lights), epigastric pain, and bilateral leg edema in a pregnant woman is highly suggestive of preeclampsia. The presence of hyperreflexia and clonus further supports this diagnosis. 2. Further Investigations:    - Blood Tests:      - Complete Blood Count (CBC): To check for thrombocytopenia, which can occur in preeclampsia.      - Liver Function Tests: Elevated liver enzymes can occur in preeclampsia and help differentiate it from other conditions.      - Renal Function Tests: To assess kidney function, as preeclampsia can affect renal function.      - Serum Uric Acid: May be elevated in preeclampsia.      - Coagulation Profile: To assess for any coagulopathy.    - Fetal Monitoring:      - Ultrasound: To assess fetal growth and amniotic fluid volume.      - Non-Stress Test or Biophysical Profile: To monitor fetal well-being. 3. Management:    - Blood Pressure Control: Antihypertensive therapy to manage severe hypertension. Medications like labetalol, nifedipine, or methyldopa are commonly used.    - Magnesium Sulfate: For seizure prophylaxis, as there is a risk of eclampsia.    - Hospitalization and Close Monitoring: Both maternal and fetal monitoring are necessary, including frequent blood pressure checks, and assessment of urine output, and laboratory values.    - Delivery Planning: Depending on the gestational age and severity of the preeclampsia, as well as the fetal condition, delivery may be indicated. This should be done in coordination with obstetricians, and possibly neonatologists if preterm delivery is anticipated.    - Counseling and Support: Providing information about the condition, potential outcomes, and management plans.   Preeclampsia is a serious condition in pregnancy that requires immediate medical attention and often necessitates close monitoring and potential early delivery to ensure the safety of both the mother and the baby. The management plan should be individualized based on the severity of the condition, gestational age, and the fetal condition. | 1. 6  2. 5  3. 6 | 1. 3  2. 3  3. 3 |

**Case 8** (Reference case 3)

| **Participants** | **Generated answer** | **Accuracy scores** | **Completeness scores** |
| --- | --- | --- | --- |
| **GP** | 1. Epiglottitis  2. Bacterial culture using exudates, Complete blood counts, Serum electrolytes, Serum ESR/CRP, Cervical X-ray  3. If oxygen saturation falls and mental status changes, airway intubation should be applied. Otherwise, empirical antibiotics (amoxicillin/clavulanate) can be applied before bacterial culture results are drawn. To reduce fever and inflammation, NSAIDs, such as naproxen can be taken. Next, he should be referred to ENT department to meet appropriate following medication needs. | 1. 5  2. 3  3. 3 | 1. 2  2. 2  3. 1 |
| **R3** | 1. epiglottitis  2. neck x-ray, abga, CBC, CRP, blood culture, neck CT if needed  3. early intubation, tracheostomy if intubation failed. IV antibiotics and steroid, epinephrine nebulizer | 1. 5  2. 5  3. 5 | 1. 2  2. 2  3. 2 |
| **R4** | 1. Deep neck infection / Acute epiglottitis  2. Neck Lat X-ray, contrast enhanced neck CT  3. Epinephrine nebulizer, IV streoid, keep the patient sitting position, bedside crycothyroidotomy prep for definite airway in case | 1. 5  2. 4  3. 4 | 1. 2  2. 2  3. 1 |
| **Chat-GPT** | The clinical presentation and examination findings of this 48-year-old man strongly suggest a diagnosis of acute epiglottitis, which is a potentially life-threatening condition characterized by inflammation of the epiglottis and surrounding structures.   1. Diagnosis: Acute Epiglottitis    - Reasoning: The key features supporting this diagnosis are:      - Severe sore throat and hoarseness.      - Painful swallowing (odynophagia).      - Inspiratory stridor and extended neck positioning, indicative of airway obstruction.      - High fever and tachycardia.      - Drooling and difficulty speaking, suggesting significant oropharyngeal involvement.      - Examination findings of a cherry red, swollen epiglottis, and edematous aryepiglottic folds.      - No history of chronic illness or immunosuppression, which is consistent with the acute nature of this condition. 2. Investigations:    - Blood Tests: Complete blood count (CBC), inflammatory markers (like C-reactive protein or erythrocyte sedimentation rate), and blood cultures.    - Imaging: Lateral neck X-ray (soft tissue) might show a swollen epiglottis ("thumb sign"). However, in a severely ill patient, it's important not to delay treatment for imaging.    - Microbiology: Throat swab cultures and, if intubation is required, samples from the epiglottis for culture to identify the causative organism.    - Monitoring: Continuous monitoring of vital signs and oxygen saturation. 3. Management:    - Immediate:      - Secure the airway. In severe cases, this might necessitate endotracheal intubation, preferably in a controlled setting like an operating room with an experienced anesthesiologist, as there's a risk of complete airway obstruction.      - Administer high-flow oxygen.    - Medical:      - Intravenous antibiotics: A broad-spectrum antibiotic like ceftriaxone or meropenem, often combined with vancomycin or clindamycin to cover both typical and atypical pathogens, including MRSA.      - Steroids: Such as dexamethasone, to reduce airway edema.      - Intravenous fluids for hydration and to maintain blood pressure.    - Monitoring and Support:      - Admission to an intensive care unit for close monitoring of the airway.      - Regular re-assessment of the airway, breathing, and circulation.   It's crucial to act swiftly with acute epiglottitis due to the rapid progression and potential for airway obstruction. Avoid agitating the patient unnecessarily, as this can precipitate complete airway closure. | 1. 6  2. 5  3. 6 | 1. 3  2. 3  3. 3 |

**Case 9** (Reference case 6)

| **Participants** | **Generated answer** | **Accuracy scores** | **Completeness scores** |
| --- | --- | --- | --- |
| **GP** | 1. Diabetic foot is caused by diabetes mellitus, and diabetic ketoacidosis and septic condition caused by diabetic foot should be ruled out.  2. Blood sugar level test, Serum electrolytes for potassium level check, blood bacterial culture, serum SER/CRP.  3. Blood sugar level test should be performed first. Simultaneously, at least 500ml of normal saline should be applied intravenously, and be rapidly dropped to lower blood sugar levels. Before applying insulin, serum potassium level should be corrected. After correction of acid/base conditions, his foot should be sterilized by povidone solution. After checking the bacterial infection, he can be referred to the General Surgery department or the Plastic Surgery department to consider the ambulation of the feet or removal of necrotizing lesion of the skin, respectively. | 1. 4  2. 3  3. 3 | 1. 1  2. 2  3. 1 |
| **R3** | 1. septic shock due to rt shin cellulitis, r/o impending DKA  2. blood sugar test, blood culture, CRP, beta ketone level test  3. IV hydration, early broad-spectrum antibiotics, inotropics & IV steroid if needed | 1. 5  2. 4  3. 3 | 1. 2  2. 2  3. 1 |
| **R4** | 1. Diabetic ketoacidosis, precipitating factor could be 2, 1 could be poor compliance of insulin, 2 could be infection(Rt leg cellulitis)  2. HbA1c, blood culture, x-ray on rt lower leg  3. massive hydration, continous insulin fluid infusion, empirical antibiotics, lactate follow up | 1. 6  2. 3  3. 2 | 1. 2  2. 1  3. 1 |
| **Chat-GPT** | The clinical presentation and investigation results suggest that this 27-year-old man with type 1 diabetes is likely suffering from diabetic ketoacidosis (DKA), complicated by a possible secondary bacterial skin infection (cellulitis) of his right lower leg.   1. Diagnosis and Precipitating Factor:    - Diabetic Ketoacidosis (DKA):      - Supporting Findings: High blood glucose (28 mmol/L), metabolic acidosis (pH 7.15, low HCO3), increased anion gap, and elevated lactate. His symptoms of nausea, vomiting, and general malaise are typical of DKA.      - Precipitating Factor: Likely the untreated or inadequately treated diabetes (poor compliance with insulin regimen and infrequent blood sugar checks) and possible infection (evidenced by erythema and tenderness on the right shin).    - Cellulitis:      - Evidence: Erythematous, warm, tender area on the right shin. This could be the precipitating factor for the DKA, as infections are common triggers. 2. Further Investigations:    - Infection Workup: Blood cultures, wound swab from the leg if there is any open area or discharge.    - Ketone Measurement: Urinary or blood ketones to confirm the presence of ketosis.    - Further Blood Tests: Electrolytes (to monitor and correct as needed, particularly potassium), renal function tests (creatinine and urea already indicate possible dehydration or pre-renal azotemia).    - Imaging: If there's suspicion of a deeper infection in the leg (like osteomyelitis), imaging such as an X-ray or MRI might be indicated. 3. Initial Management Steps:    - Fluid Resuscitation: Start with isotonic saline to correct dehydration and improve circulatory volume.    - Insulin Therapy: Continuous intravenous infusion of regular insulin to reduce blood glucose levels and suppress ketogenesis.    - Electrolyte Management: Particularly potassium, as insulin treatment will drive potassium back into cells, potentially leading to hypokalemia.    - Monitoring: Frequent monitoring of vital signs, blood glucose, ketone levels, and electrolytes. Regular reassessment of acid-base status with venous blood gases.    - Address Infection: Start empirical antibiotics after obtaining cultures, considering skin and soft tissue infection coverage.    - Education and Long-term Management: Once stabilized, the patient should receive education regarding diabetes management, particularly insulin therapy and monitoring blood glucose levels.   Prompt recognition and treatment of DKA are crucial to prevent potentially fatal complications. The simultaneous management of the underlying infection is also essential to fully resolve the DKA episode. | 1. 6  2. 6  3. 6 | 1. 3  2. 3  3. 3 |

**Case 10** (Reference case 24)

| **Participants** | **Generated answer** | **Accuracy scores** | **Completeness scores** |
| --- | --- | --- | --- |
| **GP** | 1. Leukopenia caused by chemotherapy  2. Investigations for infection caused by leukopenia should be taken. Blood bacterial cultures, Serum CRP/ESR.  3. Empirical antibiotics, such as 3rd generation cephalosporins should be applied. To decide whether chemotherapy continues, he should be referred to the Hematology & Oncology department and the Infectious Disease department for the further usage of antibiotics. | 1. 3  2. 2  3. 2 | 1. 1  2. 1  3. 1 |
| **R3** | 1. septic shock due to neutropenic fever  2. blood culture, blood gas analysis, lactic acid, CRP, chest X-ray  3. isolation, broad-spectrum antibiotics(4th cepha), IV hydration, inotropics if needed | 1. 5  2. 4  3. 5 | 1. 2  2. 2  3. 2 |
| **R4** | 1. neutropenic fever d/t recent Chemotherapy  2. lactate, inflammation marker including crp and procalcitonin / other physical exam and lab, imaging test to find any other infection source blood culture, urine analysis, chest x-ray  3. proper hydration, isolation from other patients to protect this patient, 4th generation cephalosporin IV antibiotics | 1. 4  2. 5  3. 5 | 1. 2  2. 2  3. 2 |
| **Chat-GPT** | The clinical presentation and laboratory findings for this 27-year-old man indicate a diagnosis of febrile neutropenia, a common and potentially serious complication of chemotherapy.   1. Diagnosis and Underlying Pathophysiology:    - Febrile Neutropenia:      - Supporting Evidence: Fever (38.9°C), profound neutropenia (neutrophil count of 0.1), and recent chemotherapy (BEP regimen for testicular cancer).      - Pathophysiology: Chemotherapy, particularly regimens like BEP, can cause bone marrow suppression, leading to a decrease in the production of white blood cells, including neutrophils. Neutrophils are crucial for fighting infections; thus, their reduction significantly increases the risk of serious infections. Fever in this context is a sign of possible infection. 2. Further Assessment and Investigations:    - Blood Cultures and Infection Workup: Obtain at least two sets of blood cultures (from different sites), urine culture, and cultures from any other suspected infection sites.    - Chest X-ray: To rule out respiratory infections, which may not present with typical signs in the setting of neutropenia.    - Complete Blood Count (CBC) and Differential: To monitor the degree of neutropenia and other cell lines.    - Serum Biochemistry: Including renal and liver function tests.    - Review of Medications: Particularly recent chemotherapy and antibiotic use.    - Thorough Physical Examination: Including skin, oral cavity, catheter sites, and any other potential sources of infection. 3. Key Principles in Treating This Patient:    - Empirical Broad-Spectrum Antibiotics: Immediate initiation is critical, even before identification of an infectious agent, due to the high risk of severe infection in neutropenic patients.    - Fluid Resuscitation: To address hypotension and potential sepsis.    - Close Monitoring: Vital signs, oxygen saturation, and clinical status need to be continuously monitored.    - Supportive Care: Management of fever, nutrition, and general care.    - Growth Factor Support: Consideration of granulocyte colony-stimulating factor (G-CSF) to stimulate neutrophil production.    - Adjust Antibiotics Based on Culture Results: Once specific pathogens are identified, tailor the antibiotic regimen accordingly.    - Monitor for Side Effects and Complications of Chemotherapy: Such as renal impairment and electrolyte imbalances.    - Patient Education: Informing the patient about the importance of prompt medical attention for any signs of infection in the context of neutropenia.   Febrile neutropenia is a medical emergency in oncology, requiring prompt hospitalization and aggressive management to prevent serious complications, including severe sepsis and death. | 1. 5  2. 6  3. 6 | 1. 2  2. 2  3. 3 |

**Case 11** (Reference case 50)

| **Participants** | **Generated answer** | **Accuracy scores** | **Completeness scores** |
| --- | --- | --- | --- |
| **GP** | 1. Toxic epidermal necrolysis, most likely.  2. Bacterial culture using pus for further antibiotics usage. X-ray for upper extremities.  3. He should be referred to the General Surgery department to decide to perform fasciotomy of affected lesion. | 1. 1  2. 3  3. 1 | 1. 1  2. 2  3. 1 |
| **R3** | 1. necrotizing fascitis, cellulitis  2. blood culture, CBC, CRP, coagulation test, forearm&hand CT with contrast  3. IV hydration, IV antibiotics, OS consult for debridement | 1. 4  2. 4  3. 4 | 1. 2  2. 2  3. 2 |
| **R4** | 1. pyogenic tenosynovitis  2. hand to forearm MRI, Blood test (CBC, routine chemistry lab, CRP, blood culture, lactate)  3. start empirical antibiotics as 1st generation cephalosporin if the patient did not use any antibiotics, if the patient already used other antibiotics, than use Piperacillin/Tazobactam + vancomycin | 1. 4  2. 4  3. 4 | 1. 1  2. 2  3. 2 |
| **Chat-GPT** | 1. Likely Diagnosis:    - Cat Bite-Induced Infection with Possible Tendon Sheath Involvement:      - Supporting Evidence: Progressive swelling, pus discharge from the wound, severe pain, inability to open the hand, fever, and tachycardia. The serpiginous line extending along the forearm could indicate lymphangitis. The presence of pain along the flexor tendons and pain on passive extension suggests possible involvement of the flexor tendon sheaths, which could be a sign of a deeper infection such as tenosynovitis.    - Risk Factors: Cat bites are known for causing deep tissue infections due to the nature of the wounds they inflict. 2. Initial Investigations:    - Blood Tests:      - Complete Blood Count (CBC): To look for evidence of infection (elevated white blood cells).      - Inflammatory Markers: Such as C-reactive protein (CRP) or erythrocyte sedimentation rate (ESR).      - Blood Cultures: To identify any bacteria that may have spread to the bloodstream.    - Wound Culture: To identify the causative organism and guide antibiotic therapy.    - Further Imaging: Although not immediately evident on initial radiographs, consider ultrasound or MRI to assess the extent of soft tissue involvement, especially if tenosynovitis is suspected. 3. Initial Management:    - Antibiotic Therapy: Start empirical antibiotics immediately. Cat bites often involve Pasteurella multocida, so a beta-lactam/beta-lactamase inhibitor (e.g., amoxicillin-clavulanate) is a good choice. Coverage for other common skin flora and potential resistant organisms should also be considered.    - Surgical Consultation: Prompt referral to a hand surgeon is crucial, especially if there is suspicion of tenosynovitis or if there is an abscess that requires drainage.    - Pain Management: Adequate analgesia to manage severe pain.    - Tetanus Prophylaxis: Update tetanus vaccination if necessary.    - Elevation and Immobilization of the Affected Hand: To reduce swelling and pain.    - Monitoring: Close monitoring of vital signs and local signs of infection to assess response to treatment.   Cat bite wounds, especially to the hand, can lead to severe infections that require aggressive management, including both medical and potentially surgical interventions. Prompt treatment is essential to prevent the spread of infection and reduce the risk of complications. | 1. 5  2. 6  3. 5 | 1. 3  2. 3  3. 3 |

**Case 12** (Reference case 61)

| **Participants** | **Generated answer** | **Accuracy scores** | **Completeness scores** |
| --- | --- | --- | --- |
| **GP** | 1. Hernia  2. Complete blood count, Serum electrolytes, Abdominal X-ray  3. The patient should be referred to General Surgery department for taxis surgery of hernia. If necessary, necrotized lesion can be removed. | 1. 1  2. 2  3. 2 | 1. 1  2. 1  3. 1 |
| **R3** | 1. r/o proctocolitis  2. CBC, blood gas analysis, CRP, abdominal x-ray, if needed) abdominal CT with contrast  3. IV hydration, GI medication | 1. 3  2. 4  3. 3 | 1. 2  2. 2  3. 2 |
| **R4** | 1. could be diverticulitis  2. lab test including CBC, routine chemistry, inflammation marker / most important thing could be contrast enhanced Computed tomography in Abdomino-pelvic area  3. painkiller, 3rd Generation cephalosporin + metronidazole | 1. 4  2. 4  3. 4 | 1. 1  2. 2  3. 2 |
| **Chat-GPT** | 1. Diagnosis:    - Diverticulitis:      - Supporting Evidence: The key features include constant left iliac fossa pain, fever, a history of constipation, and tenderness and guarding localized to the left iliac fossa. Diverticulitis typically occurs in patients over the age of 40 and is often associated with a history of constipation or other bowel habit changes. 2. Investigations:    - Blood Tests:      - Complete Blood Count (CBC): To assess for leukocytosis indicative of infection.      - C-reactive protein (CRP): As an indicator of inflammation.      - Serum Electrolytes, Renal Function, and Liver Enzymes: To evaluate overall organ function.    - Imaging:      - Abdominal CT Scan with Contrast: The gold standard to confirm the diagnosis of diverticulitis and assess the extent of inflammation or any complications like abscess formation.      - Abdominal Ultrasound: Can be an alternative if CT is not readily available or if there are contraindications to CT (such as allergy to contrast material or pregnancy).    - Urine Analysis: To rule out urinary tract infection, which can sometimes present similarly. 3. Management:    - Antibiotics: Broad-spectrum antibiotics are indicated for treating the infection. Choices may include a combination of metronidazole with either a fluoroquinolone or a beta-lactam antibiotic.    - Pain Management: Adequate analgesia to control pain.    - Fluid Resuscitation: Intravenous fluids if the patient is dehydrated or unable to maintain adequate oral intake.    - Dietary Management: Initially, a clear liquid diet is often recommended, progressing to a low-fiber diet as symptoms improve.    - Monitoring: Close monitoring for signs of improvement or complications. Severe cases or complications like abscesses may require hospital admission.    - Follow-up: Once the acute episode resolves, follow-up colonoscopy is usually recommended to assess for any underlying pathology, like colorectal cancer, and to confirm the diagnosis of diverticulosis.   Early recognition and appropriate management of diverticulitis are essential to prevent complications and ensure effective treatment. | 1. 6  2. 6  3. 5 | 1. 3  2. 3  3. 3 |

**Case 13** (Reference case 89)

| **Participants** | **Generated answer** | **Accuracy scores** | **Completeness scores** |
| --- | --- | --- | --- |
| **GP** | 1. Ectopic pregnancy is highly suspected, r/o pregnancy, r/o adnexal torsion and r/o endometritis caused by IUCD should be excluded.  2. Vaginoscopy, intravaginal ultrasound, bacterial culture using vaginal discharge, complete blood count, serum electrolytes. Seum ESR/CRP.  3. After bacterial culture and other labs are performed, she should be referred to OBGY department, and adnexectomy should be considered. | 1. 4  2. 3  3. 3 | 1. 2  2. 2  3. 1 |
| **R3** | 1. PID, fitz-hugh curtis syndrome. DDx : appendicitis  2. CBC, CRP, coagulation test, abdominal x-ray. abdominal CT with contrast  3. hydration, antibiotics, pain control | 1. 4  2. 4  3. 4 | 1. 2  2. 2  3. 1 |
| **R4** | 1. ectopic pregnancy, ddx could be pelvic inflammatory disease with Fitz-Hurg-Curtis  2. urine hcg, trans-abominal/vaginal ultrasonography, if Urine hcg confim neg pregnancy, than use contrast enhanced Abdomino-pelvic CT included arterial phase to find out FHC  3. massive hydration, painkiller, RBC prep for transfusion. | 1. 4  2. 5  3. 4 | 1. 2  2. 2  3. 1 |
| **Chat-GPT** | The clinical presentation of this 26-year-old woman suggests an ectopic pregnancy as the primary working diagnosis, with other considerations as differential diagnoses.   1. Working Diagnosis and Differential Diagnoses:    - Ectopic Pregnancy:      - Supporting Evidence: Severe sharp lower abdominal pain, particularly on the right side, dizziness, shoulder tip pain (suggesting diaphragmatic irritation), history of a missed period, spotting, and the presence of an IUCD. The history of sexually transmitted infection (chlamydia) increases the risk of ectopic pregnancy.    - Differential Diagnoses:      - Ruptured ovarian cyst.      - Appendicitis.      - Pelvic inflammatory disease (PID).      - Miscarriage. 2. First and Further Required Investigations:    - First Investigation:      - Serum Beta-hCG (Human Chorionic Gonadotropin): To confirm pregnancy and assist in diagnosing an ectopic pregnancy.    - Other Investigations:      - Transvaginal Ultrasound: To locate the pregnancy and check for signs of ectopic pregnancy.      - Complete Blood Count (CBC): Already done, to assess for anemia or infection.      - Blood Type and Rh Factor: Important in case Rhesus-negative women need anti-D immunoglobulin.      - Liver and Renal Function Tests: To assess overall health status. 3. Management in the Emergency Department:    - Stabilization: Ensure hemodynamic stability, given her low blood pressure and tachycardia, which may indicate internal bleeding. Intravenous fluid resuscitation may be necessary.    - Pain Management: Continue and adjust as necessary.    - Gynecological Consultation: Urgent referral to a gynecologist or obstetrician for further assessment and management, particularly if an ectopic pregnancy is confirmed or strongly suspected.    - Surgical Intervention: If an ectopic pregnancy is confirmed and depending on its location and the patient's stability, surgical intervention (such as laparoscopy) may be necessary.    - Rh Immunoglobulin Administration: If the patient is Rh-negative and there is any suspicion of pregnancy loss or ectopic pregnancy.    - Monitoring: Close observation for any signs of worsening condition, including increasing pain, dizziness, or changes in vital signs.   Ectopic pregnancy is a medical emergency that requires prompt diagnosis and intervention to prevent serious complications. The presence of an IUCD and a history of chlamydia increase the risk for ectopic pregnancy, making it a top differential diagnosis in this case. | 1. 6  2. 6  3. 5 | 1. 3  2. 2  3. 3 |

**Case 14** (Reference case 96)

| **Participants** | **Generated answer** | **Accuracy scores** | **Completeness scores** |
| --- | --- | --- | --- |
| **GP** | 1. Amniotic fluid embolism  2. serum electrolytes, complete blood count, chest CT.  3. If her vital sign maintains stable, close observation is needed. However, if blood pressure falls and heart rate still increases, she should be referred to the department of Cardiac Surgery for embolectomy or using embolytic drugs. | 1. 1  2. 2  3. 3 | 1. 1  2. 1  3. 2 |
| **R3** | 1. pulmonary thromboembolism  2. coagulation test, d-dimer, pulmonary angio CT  3. O2 supply, TPa or heparinization | 1. 5  2. 4  3. 4 | 1. 2  2. 2  3. 2 |
| **R4** | 1. Pulmonary Thromboembolism  2. Echocardiogram. consider pulmonary angio CT but she is pregant so i would like to start with EChocardiogram  3. massive hydration to prevent RV collapse, IV heparin use. | 1. 5  2. 4  3. 5 | 1. 2  2. 2  3. 2 |
| **Chat-GPT** | The clinical presentation and findings in this 30-year-old pregnant woman are suggestive of a pulmonary embolism (PE).   1. Diagnosis:    - Pulmonary Embolism:      - Supporting Evidence: Sudden onset of shortness of breath, right-sided chest pain worsened by inspiration, hypoxia (oxygen saturation of 91% on room air), tachycardia, and the 'S1, Q3, T3' pattern on the ECG are all indicative of a PE. Pregnancy increases the risk for thromboembolic events, and her elevated BMI further contributes to this risk. 2. Further Investigations:    - D-Dimer Test: However, it should be noted that D-dimer levels are commonly elevated in pregnancy and may not be as specific.    - CT Pulmonary Angiography (CTPA): The gold standard for diagnosing PE. However, considering her pregnancy, a risk-benefit analysis should be performed, considering potential radiation exposure to the fetus.    - Venous Doppler Ultrasound of the Legs: To check for deep vein thrombosis (DVT), which can be a source of emboli.    - Echocardiography: To assess for signs of right heart strain and to rule out other cardiac causes of her symptoms. 3. Management:    - Anticoagulation: Immediate initiation of anticoagulation is critical in suspected PE. Low molecular weight heparin (LMWH) is preferred in pregnancy due to its safety profile for both the mother and fetus.    - Oxygen Therapy: To maintain oxygen saturation above 94%.    - Hemodynamic Support: Fluid resuscitation and/or vasopressors if necessary to manage hypotension.    - Consultation with Multidisciplinary Team: Involving obstetrics, pulmonology, and possibly hematology, for coordinated care and decision-making regarding anticoagulation and monitoring.    - Follow-up and Monitoring: Regular monitoring of maternal and fetal well-being, adjusting anticoagulation therapy as needed, and postpartum planning for thromboembolism prophylaxis.    - Patient Education: Counseling about the signs and symptoms of PE and DVT, and the importance of adherence to anticoagulation therapy.   Pulmonary embolism in pregnancy is a serious condition that requires immediate intervention and coordinated care to ensure the safety of both the mother and the fetus. The management plan should be tailored to balance the risks and benefits of diagnostic procedures and treatment in the context of pregnancy. | 1. 6  2. 6  3. 6 | 1. 3  2. 2  3. 3 |

**Case 15** (Reference case 16)

| **Participants** | **Generated answer** | **Accuracy scores** | **Completeness scores** |
| --- | --- | --- | --- |
| **GP** | 1. Community-acquired pneumonia  2. Sputum culture, Chest X-ray, Chest CT, CBC, routine chemistry including serum CRP and lactate, blood culture  3. The carbon dioxide and carbonate ion concentration is slightly higher than normal. This has led to slight respiratory acidosis. Its severity is not that great, and probably does not require immediate attention. However, the patient has low oxygen concentration. High-flow oxygen should be administered. Other than this, the patient should be started on empirical antibiotics, most preferably a third generation cephalosporin with or without flouroquinolone. After culture results are returned, the antibiotics should be managed accordingly. | 1. 2  2. 4  3. 3 | 1. 1  2. 2  3. 2 |
| **R3** | 1. COPD exacerbation due to klebsiella infection  2. CXR, CBC and routine blood chemistry, Chest CT, EKG, cardiac enzyme, blood culture  3. Respiratory Acidosis with metabolic compensation, intial management: Non-invasivie positive pressure ventilation, Anticholingeric nebulizer, antibiotics treatment | 1. 3  2. 4  3. 4 | 1. 2  2. 2  3. 2 |
| **R4** | 1. copd ae combined pneumonia  2. chest xray, chest ct, blood test  3. hypoxia, hypercapnia, respiratory acidosis, management-> o2 apply, ventolin nebulizer, iv streoid, antibiotics | 1. 4  2. 3  3. 4 | 1. 2  2. 2  3. 2 |
| **Chat-GPT** | The clinical presentation and investigation results for this 71-year-old man with a history of chronic obstructive pulmonary disease (COPD) point towards an acute exacerbation of COPD, likely complicated by a bacterial respiratory infection.   1. Likely Diagnosis:    - Acute Exacerbation of COPD:      - Supporting Signs: Increased shortness of breath, productive cough with a change in sputum color (indicating a possible infection), and a history of COPD.      - Examination Findings: Marked dyspnea at rest, tachycardia, low oxygen saturation on room air, and widespread wheezing on lung auscultation. 2. Investigations in the Emergency Department (ED):    - Chest X-ray: To look for signs of infection, congestion, or other complications like pneumothorax.    - Sputum Culture and Sensitivity: Given the change in sputum color, to identify any bacterial pathogens and appropriate antibiotic sensitivity.    - Complete Blood Count (CBC): To check for signs of infection or anemia.    - Electrolytes, Renal Function, and Blood Glucose: As comorbid conditions and medications can affect these parameters.    - ECG (Electrocardiogram): To assess cardiac status, given his tachycardia and the potential for cardiac complications in COPD exacerbations. 3. ABG Analysis and Initial Management:    - ABG Results:      - pH 7.28: Acidotic.      - pO2 6.9 kPa: Hypoxemia.      - pCO2 6.7 kPa: Near the upper limit of normal, suggesting possible respiratory failure.      - HCO3 28 mmol/L: Compensated metabolic alkalosis, likely a compensatory mechanism for respiratory acidosis.    - Interpretation: The ABG suggests respiratory acidosis with partial compensation, consistent with acute respiratory failure due to COPD exacerbation.    - Initial Management:      - Oxygen Therapy: To improve oxygenation. Target saturation should be 88-92% in COPD patients to avoid worsening hypercapnia.      - Bronchodilators: Nebulized beta-agonists (e.g., salbutamol) and anticholinergics (e.g., ipratropium).      - Corticosteroids: Systemic steroids to reduce airway inflammation.      - Antibiotics: Empirical antibiotics if a bacterial infection is suspected based on sputum changes and clinical presentation.      - Fluid Management: Adequate hydration is important, but care must be taken to avoid fluid overload.      - Venous Thromboembolism Prophylaxis: Given the risk due to reduced mobility and acute illness.      - Monitoring: Close monitoring of respiratory status, ABG, and response to treatment.   This patient's condition requires careful management, balancing the need for oxygenation against the risk of worsening hypercapnia. The management should be dynamic and responsive to the patient's evolving clinical state. | 1. 5  2. 5  3. 6 | 1. 3  2. 2  3. 3 |

**Case 16** (Reference case 25)

| **Participants** | **Generated answer** | **Accuracy scores** | **Completeness scores** |
| --- | --- | --- | --- |
| **GP** | 1. Lobar pneumonia, RLL. The patient shows relative bradycardia. The most likely causative microbe is legionella.  2. Chest CT, Chest X-ray, sputum culture, serum CRP, lactate, blood culture  3. The patient should immediately be started on empirical antibiotics, most preferably a third generation cephalosporin with or without flouroquinolone. After culture results are returned, the antibiotics should be managed accordingly. Nasal oxygen can help manage her shortness of breath. | 1. 2  2. 4  3. 2 | 1. 1  2. 2  3. 2 |
| **R3** | 1. Pnuemonia c sepsis, r/o Tuberculosis, Lobar pneumonia is more likely  2. blood gas analysis, CXR, routine blood chemistry and CBC, urine analysis, consider chest CT, lactic acid level, ESR/CRP  3. Antipyretics, Blood culture, Antibiotics, IV fluids | 1. 3  2. 5  3. 4 | 1. 2  2. 2  3. 2 |
| **R4** | 1. pneumonia, bronchitis, .. pneumonia is most likely diagnosis  2. chest xray, ct scan if needed, inflammatory marker blood test  3. antibiotics , cough, sputum reliever | 1. 5  2. 4  3. 3 | 1. 2  2. 2  3. 1 |
| **Chat-GPT** | The clinical presentation and investigation results of this 70-year-old woman suggest a few differential diagnoses, with the most likely being a lower respiratory tract infection, such as pneumonia.   1. Differential Diagnoses:    - Community-Acquired Pneumonia (CAP):      - Supporting Evidence: Worsening productive cough with yellow-green sputum, fatigue, cough pain, fever, and crackles at the right base of the lung on examination.    - Other Possibilities:      - Acute bronchitis, particularly given the cough and sputum production.      - Congestive heart failure, considering her age and hypertension, although the lack of cardiac findings on examination makes this less likely.      - Pulmonary embolism, especially if there is sudden onset of symptoms, but again less likely given the presentation. 2. Further Investigations:    - Chest X-ray: To confirm pneumonia and assess the extent and location of any lung infiltrates.    - Blood Cultures: If there is suspicion of bacteremia or severe infection.    - Sputum Culture and Sensitivity: Particularly useful if pneumonia is suspected, to identify the causative organism and guide antibiotic therapy.    - Complete Blood Count (CBC): To monitor white blood cell count, which can be indicative of infection.    - Arterial Blood Gas (ABG) or Venous Blood Gas (VBG): To assess oxygenation and ventilation status.    - Influenza and Other Respiratory Virus PCR Testing: Depending on the season and local prevalence. 3. Treatment Initiation:    - Empirical Antibiotic Therapy: For suspected pneumonia, a broad-spectrum antibiotic such as amoxicillin-clavulanate or a respiratory fluoroquinolone might be started, depending on local resistance patterns and patient allergies.    - Supportive Care: Including oxygen supplementation to maintain oxygen saturation >94%, hydration, and fever management.    - Blood Sugar Control: Monitoring and managing her diabetes, as infections can cause fluctuations in blood glucose levels.    - Close Monitoring: Of respiratory status, vital signs, and response to treatment.    - Review and Adjust Treatment: Based on results from further investigations, particularly sputum and blood cultures.   Given the patient's age and comorbidities, a careful and thorough approach is necessary to manage her condition effectively and prevent complications. | 1. 6  2. 4  3. 5 | 1. 3  2. 3  3. 2 |

**Case 17** (Reference case 62)

| **Participants** | **Generated answer** | **Accuracy scores** | **Completeness scores** |
| --- | --- | --- | --- |
| **GP** | 1. Arterial obstruction due to thrombus, possibly originating from atrial fibrillation  2. Compression doppler on the right leg, angiography of the right leg, EKG, coagulation time  3. The patient should immediately go through vascular intervention. After the thrombus is removed, further investigation regarding his arrhythmia should be performed. Anti-arrhythmic agents should be considered. Taking into account his long history of smoking and type 2 DM, there may be peripheral aneurysms or PAOD involved. If any of these are found, proper management including stenting or surgical approach can be recommended. | 1. 4  2. 3  3. 3 | 1. 2  2. 2  3. 2 |
| **R3** | 1. r/o PAOD (most likely), r/o right popliteal vein DVT  2. routine chemistry, ESR/CRP, D-dimer, PT/aPTT, EKG, cardiac enzmye. Low Extremity Angio CT  3. Administration of thrombolytic agent | 1. 4  2. 4  3. 3 | 1. 2  2. 2  3. 1 |
| **R4** | 1. PAOD, rt leg  2. lower extremity angio ct scan for vessel work up  3. emergency angiography, pain control | 1. 4  2. 3  3. 3 | 1. 2  2. 2  3. 1 |
| **Chat-GPT** | The clinical presentation of this 84-year-old male is suggestive of acute limb ischemia, particularly affecting the right leg.   1. Diagnosis:    - Acute Limb Ischemia:      - Supporting Evidence: Sudden onset of severe leg pain, pallor, coldness, loss of sensation, absence of pulses below the knee, inability to move the leg, and pain on passive movement. His history of atrial fibrillation and smoking are significant risk factors for arterial thrombosis. 2. Investigations:    - Doppler Ultrasound Examination: To assess blood flow in the leg arteries and confirm the diagnosis of ischemia.    - Blood Tests:      - Complete Blood Count (CBC): To assess for anemia or infection.      - Renal Function Tests and Electrolytes: Kidney function can be affected by poor circulation and it's important for contrast dye use if angiography is needed.      - Coagulation Profile: Especially considering his history of atrial fibrillation and aspirin use.    - ECG (Electrocardiogram): To assess cardiac rhythm and rule out myocardial infarction, which could be a source of emboli.    - CT Angiography: To visualize the arterial anatomy and identify the location and extent of the blockage. 3. Management:    - Urgent Vascular Surgery Consultation: Acute limb ischemia is a surgical emergency.    - Anticoagulation: Immediate initiation of intravenous heparin to prevent further clotting.    - Pain Management: Adequate analgesia to control severe pain.    - Supplemental Oxygen: To improve oxygenation.    - Fluid Resuscitation: If indicated, to maintain adequate circulation.    - Revascularization: Surgical or endovascular intervention is often required urgently to restore blood flow and save the limb.    - Monitoring and Supportive Care: Close monitoring of vital signs, limb status, and overall patient condition.    - Risk Factor Management: Smoking cessation, optimal control of diabetes and hypertension, and adjustment of antithrombotic therapy.   The goal in managing acute limb ischemia is to restore blood flow as quickly as possible to prevent tissue death and potential limb loss. Time is critical in this condition, and rapid intervention is key to a successful outcome. | 1. 4  2. 5  3. 6 | 1. 3  2. 3  3. 3 |

**Case 18** (Reference case 66)

| **Participants** | **Generated answer** | **Accuracy scores** | **Completeness scores** |
| --- | --- | --- | --- |
| **GP** | 1. Intestinal strangulation: the patient has previously undergone a major bowel surgery.  Ureteral obstruction: the patient has previously undergone a major bowel surgery.  Ureter stone: the patient is dehydrated.  2. Abdominal X-ray, abdominal CT, dual-energy CT, abdominal ultrasound including the kidney, serum CRP, urinalysis  3. The patient should be put in NPO status, considering the possibility of an emergency operation. If intestinal strangulation is confirmed, the patient should go through immediate surgery. The patient should also be hydrated with IV saline, with analgesics. | 1. 2  2. 2  3. 2 | 1. 2  2. 2  3. 2 |
| **R3** | 1. Ureter stone (most likely), dehydration could be a risk factor,  2. CBC/Routine chemistry, inflammatory markers, non-contrast CT or contrast Abdomen CT for ddx with crohns disease acute exacerbation  3. NSAIDS, IV hydration, metoclopromide, | 1. 4  2. 4  3. 4 | 1. 2  2. 2  3. 2 |
| **R4** | 1. ureter stone, rt , , small bowel obstruction, / risk factors, history of small bowel resection and crohn's dz  2. abdomen xray, ct scan  3. pain control, | 1. 4  2. 3  3. 3 | 1. 2  2. 2  3. 2 |
| **Chat-GPT** | The presentation of this 30-year-old man suggests a likely diagnosis of a renal colic, most probably due to a kidney stone.   1. Likely Diagnosis and Risk Factors:    - Kidney Stone (Renal Colic):      - Supporting Evidence: The characteristic description of excruciating, intermittent, and radiating pain from the flank to the groin, along with the presence of hematuria on urine dipstick, is typical of a kidney stone.      - Risk Factors: His history of Crohn’s disease and extensive small bowel resection may contribute to the risk of kidney stones, possibly due to malabsorption, changes in urine pH, and dehydration. 2. Investigations in the ED:    - Non-Contrast CT of the Abdomen and Pelvis: This is the gold standard for diagnosing kidney stones and can identify the size, location, and number of stones.    - Blood Tests:      - Complete Blood Count (CBC): To check for signs of infection or anemia.      - Serum Creatinine and Electrolytes: To assess kidney function.      - Blood Urea Nitrogen (BUN): To evaluate hydration status.    - Additional Urine Tests:      - Urine Culture: Although the dipstick was negative for nitrites and leukocytes, a culture can be useful if infection is suspected.      - 24-hour Urine Collection: For metabolic evaluation, especially if the patient has recurrent stones (usually done as an outpatient). 3. Management:    - Pain Relief: Strong analgesics are often necessary, such as nonsteroidal anti-inflammatory drugs (NSAIDs) or opioids.    - Hydration: Oral or intravenous fluids, depending on the level of dehydration and pain.    - Medical Expulsive Therapy (MET): Medications like tamsulosin may aid in the passage of the stone.    - Monitoring: Especially if there’s suspicion of infection or obstruction, which may necessitate urological intervention.    - Follow-Up and Prevention:      - Urology Referral: Especially if the stone is large (>5-6 mm) or if there are signs of infection or obstruction.      - Dietary and Lifestyle Modifications: Based on the type of stone and metabolic evaluation.      - Management of Crohn’s Disease: Optimizing treatment to reduce the risk of recurrence.   It's important to manage both the acute episode and consider long-term strategies to prevent recurrence, especially in a patient with underlying risk factors like Crohn's disease. | 1. 5  2. 5  3. 5 | 1. 2  2. 3  3. 3 |

**Case 19** (Reference case 69)

| **Participants** | **Generated answer** | **Accuracy scores** | **Completeness scores** |
| --- | --- | --- | --- |
| **GP** | 1. Otitis media, right side  2. CT scan of the head and neck, patency of the Eustachian tube, Chest X-ray  3. A ventilation tube should be place on the right tympanic membrane. Further management includes antibiotics and analgesics. | 1. 3  2. 2  3. 1 | 1. 1  2. 2  3. 2 |
| **R3** | 1. r/o Acute Otitis media due to eustachian tube dysfunction (most likely), r/o Mastoiditis  2. Otoscopic evaluation, CXR, Temporal bone CT  3. Antipyretics, Antibiotics such as Amoxicillin, PO meds for rhinitis | 1. 4  2. 3  3. 3 | 1. 2  2. 2  3. 2 |
| **R4** | 1. Acute otitis media  2. facial ct with contrast, blood test  3. po antibiotics, ex, amoxicilin, analegsia, antipyretics | 1. 3  2. 2  3. 4 | 1. 1  2. 2  3. 2 |
| **Chat-GPT** | The clinical presentation of this 4-year-old child suggests a diagnosis of mastoiditis.   1. Diagnosis:    - Mastoiditis:      - Supporting Evidence: The child has a history of worsening otalgia (ear pain), fever, and now presents with a boggy red swelling behind the ear (retroauricular area), with the pinna displaced outward. This presentation, along with the finding of a bulging, red tympanic membrane on otoscopic examination, is characteristic of mastoiditis, an infection of the mastoid bone often secondary to acute otitis media. 2. Appropriate Investigations:    - Blood Tests:      - Complete Blood Count (CBC): To assess for signs of infection.      - Inflammatory Markers: Like C-reactive protein (CRP) to gauge the severity of the infection.    - Imaging:      - CT Scan of the Temporal Bone: To assess the extent of the infection in the mastoid air cells.    - Microbiology:      - Ear Swab or Aspirate Culture (if there is drainage): To identify the causative organism and guide antibiotic therapy.    - Audiometry: Might be considered to assess hearing, particularly if there's a history of recurrent ear infections. 3. Management:    - Antibiotic Therapy: Immediate initiation of intravenous broad-spectrum antibiotics, such as ceftriaxone or amoxicillin-clavulanate, is crucial.    - Analgesia: To manage pain.    - Surgical Consultation: In cases of mastoiditis, referral to an otolaryngologist (ENT specialist) is essential. Surgical intervention, like myringotomy or mastoidectomy, may be necessary depending on the response to antibiotics and the extent of the infection.    - Monitoring and Supportive Care: Monitor the child's response to treatment, including pain level, fever, and local signs of infection.    - Follow-up: To ensure resolution of the infection and assess for any complications or hearing loss.   Mastoiditis is a potentially serious condition that requires prompt diagnosis and aggressive treatment to prevent complications such as hearing loss, intracranial spread of infection, and other potentially severe outcomes. | 1. 5  2. 6  3. 5 | 1. 3  2. 3  3. 3 |

**Case 20** (Reference case 78)

| **Participants** | **Generated answer** | **Accuracy scores** | **Completeness scores** |
| --- | --- | --- | --- |
| **GP** | 1. Community-acquired pneumonia  2. Chest X-ray, blood culture, lateral view X-ray of the head and neck  3. The patient’s vaccination status has to be thoroughly reviewed, and planned. Fluid status of the patient should be closely monitored. To keep the patient well-hydrated, his urine should be kept rather transparent. Parenteral nutrition is done only when absolutely necessary. Paradoxical breathing suggests possible epiglottic swelling, so a proper management of the airway is needed. | 1. 2  2. 2  3. 3 | 1. 1  2. 2  3. 2 |
| **R3** | 1. Acute Bronchiolitis, Dehydration  2. CXR, CBC/Routine chemistry  3. Providing warm, moisturized O2 | 1. 5  2. 3  3. 3 | 1. 2  2. 2  3. 2 |
| **R4** | 1. Acute bronchiolitis  2. Chest xray  3. Give 02 ,hydration , nasal suction, nebulizer if needed | 1. 5  2. 2  3. 3 | 1. 1  2. 2  3. 2 |
| **Chat-GPT** | The clinical presentation of this 3-month-old male suggests a diagnosis of bronchiolitis.   1. Diagnosis:    - Bronchiolitis:      - Supporting Evidence: The history of cough, coryza, increased work of breathing with labored breathing, reduced fluid intake, and the presence of crackles and wheezing on auscultation are typical of bronchiolitis. It is common in young infants and often occurs following an upper respiratory tract infection, as suggested by the history of a sibling with coryzal symptoms. The child's prematurity and previous respiratory distress might increase his susceptibility to respiratory infections. 2. Further Investigations:    - Pulse Oximetry: Continuous monitoring of oxygen saturation.    - Blood Tests: Not routinely required in bronchiolitis unless there is a suspicion of a bacterial co-infection or other complications. However, a Complete Blood Count (CBC) and C-Reactive Protein (CRP) can be considered in complex cases.    - Nasopharyngeal Swab: To identify the virus causing bronchiolitis, such as Respiratory Syncytial Virus (RSV), although this is usually not necessary for management.    - Chest X-Ray: Not routinely recommended but may be considered if there is concern for complications such as pneumonia.    - Urinalysis: To assess hydration status, especially given the history of reduced fluid intake and fewer wet nappies. 3. Key Principles in Treating This Patient:    - Supportive Care:      - Oxygen Therapy: If oxygen saturation falls below a set threshold (usually <90-92%).      - Fluid Management: Ensuring adequate hydration, which may require oral or intravenous fluids, especially if there is reduced oral intake or signs of dehydration.    - Nasal Suction: To relieve nasal congestion and facilitate feeding and breathing.    - Frequent, Smaller Feeds: To maintain nutrition while avoiding fatigue from feeding.    - Monitoring: Close observation of respiratory rate, effort, oxygen saturation, hydration status, and ability to feed.    - Bronchodilators and Steroids: Not routinely recommended in bronchiolitis but may be trialed in some cases.    - Antibiotics: Not indicated unless there is a strong suspicion or confirmation of a secondary bacterial infection.    - Education for Caregivers: Guidance on symptom management, signs of worsening illness, and when to seek further medical attention.   Bronchiolitis is primarily managed supportively, with close monitoring and intervention aimed at maintaining adequate oxygenation and hydration. Most cases can be managed at home, but hospitalization may be required in more severe cases, particularly in infants with risk factors like prematurity or a history of respiratory distress. | 1. 6  2. 5  3. 6 | 1. 3  2. 3  3. 3 |

**Case 21** (Reference case 91)

| **Participants** | **Generated answer** | **Accuracy scores** | **Completeness scores** |
| --- | --- | --- | --- |
| **GP** | 1. Most likely: torsion, left side / DDx: PID  2. Vaginal ultrasound with doppler, pelvic CT  3. If the CT scan confirms torsion, an immediate detorsion is needed. If the case is otherwise confirmed as PID, the abscess should be drained first. Further surgical approaches may be necessary according to the result of the drainage. | 1. 3  2. 4  3. 3 | 1. 2  2. 2  3. 2 |
| **R3** | 1. r/o OVarian Torsion (most likely), r/o Tuboovarian Abscess  2. urine HCG, abd sonography,  3. Surgical Intervention, IV fluids, NSAIDS, | 1. 5  2. 4  3. 4 | 1. 2  2. 2  3. 2 |
| **R4** | 1. tuboovarian abscess, ddx) ovarian cyst rupture, acute gastroenteritis,,,  2. abdomen-pelvis ct  3. iv antibiotics, analgesics, drainage if needed. | 1. 4  2. 3  3. 3 | 1. 2  2. 1  3. 2 |
| **Chat-GPT** | The clinical presentation of this 16-year-old girl suggests the need to exclude ovarian torsion as the most likely diagnosis, given the sudden onset of severe pain, localization to the left lower quadrant, and associated findings on examination and investigation.   1. Most Likely Diagnosis to be Excluded and Differential Diagnoses:    - Ovarian Torsion:      - Supporting Evidence: Sudden onset of severe pain in the left lower quadrant, worsened from colicky to constant, associated with fever, tachycardia, and localized tenderness in the left adnexa.    - Differential Diagnoses:      - Appendicitis: Although typically presents with right lower quadrant pain, it can sometimes present atypically.      - Ectopic Pregnancy: The urinary pregnancy test was negative, making this less likely.      - Pelvic Inflammatory Disease (PID): Less likely given the lack of sexual activity and vaginal discharge.      - Ruptured Ovarian Cyst: Can present with sudden onset of pain and localized tenderness. 2. Additional Investigations:    - Transvaginal or Abdominal Ultrasound: To assess the ovaries for torsion, cysts, or other abnormalities. Doppler ultrasound can assess blood flow to the ovary, which is crucial in diagnosing ovarian torsion.    - Complete Blood Count (CBC) with Differential: To check for signs of infection or inflammation.    - Further Imaging: If the diagnosis remains uncertain after ultrasound, a CT scan of the abdomen and pelvis may be considered to rule out other causes of abdominal pain. 3. Management:    - Pain Management: Administer appropriate analgesia for pain control.    - Fluid Resuscitation: Ensure adequate hydration, especially if there has been vomiting.    - Gynecological Consultation: Urgent referral to a gynecologist is necessary, as ovarian torsion requires immediate surgical intervention to preserve ovarian function.    - Surgical Intervention: If torsion is confirmed, laparoscopic surgery to untwist the ovary and possibly fixate it to prevent recurrence.    - Monitoring and Supportive Care: Monitor vital signs and pain level, and provide support and reassurance to the patient and her family.   Ovarian torsion is a gynecological emergency requiring prompt diagnosis and intervention to prevent loss of the ovary. The clinical presentation can be similar to other causes of acute abdominal pain in females, making thorough evaluation and rapid diagnosis critical. | 1. 6  2. 5  3. 5 | 1. 3  2. 2  3. 3 |

**Case 22** (Reference case 17)

| **Participants** | **Generated answer** | **Accuracy scores** | **Completeness scores** |
| --- | --- | --- | --- |
| **GP** | 1. SVC syndrome can be the impression due to shortness of breath, low bp, elevated JVP, Hx of multiple myeloma. PTE secondary to DVT can be the candidate due to shortness of breath, swollen left leg, low bp. TLS can also be the candidate due to Hx of multiple myeloma, shortness of breath, low bp. The most likely diagnosis is TLS  2. Close inspection of face(for the confirmation of SVC syndrome), Blood tests like CBC, electrolyte(p, uric acid, potassium, calcium and so on), D-dimer, routine chemistry(ast, alt, Cr and so on) Doppler US of lower extremities. Above tests are needed in this order. And normal saline and loop diuretics are needed to manage this patient.  3. Further investigations like chest CT, chest x-ray are needed to treat this patients. And treatment of TLS is needed and reevaluation of multiple myeloma is needed. See if there are hypercalcemia, renal dysfunction, anemia, bone lesion) | 1. 3  2. 3  3. 3 | 1. 2  2. 2  3. 2 |
| **R3** | 1. cardiogenic shock due to heart failure, possible tamponade, also pulmonary thromboembolism  2. cardiac enzyme, pro-BNP, D-dimer, routine chemistry, electrolyte, pulmonary angio ct  3. IV hydration, vasopressor if necessary, If tamponade is the diagnosis, needle decompression is necessary, If Pulmonary thromboembolism is the diagnosis, anticoagulation is necessary | 1. 4  2. 4  3. 4 | 1. 2  2. 2  3. 2 |
| **R4** | 1. most likely : pulmonary embolism // differential diagnosis : myocardial infarction, heart failure, anemia due to multiple myeloma  2. lab(cardiac enzyme, d-dimer, CBC), pulmonary angio CT, lower extremity CT, echocardiogram  3. more oxygen therapy, the consideration for mechanical thrombectomy, anticoagulants like NOACs or warfarin, cardiac revascularization therapy if MI diagnosed | 1. 4  2. 5  3. 5 | 1. 2  2. 2  3. 2 |
| **Chat-GPT** | Based on the history and clinical findings of this 60-year-old man with multiple myeloma, several differential diagnoses can be considered, with the most likely being a pulmonary embolism (PE).   1. Differential Diagnosis and Most Likely Diagnosis:    - Pulmonary Embolism (PE):      - Supporting Evidence: Sudden onset of shortness of breath, collapse, hypotension, tachycardia, hypoxemia (despite supplemental oxygen), elevated JVP, and right bundle branch block (RBBB) on ECG. The presence of unilateral leg swelling further supports this diagnosis.      - Risk Factors: History of multiple myeloma (increased risk of thrombosis), oral chemotherapy, and smoking.    - Other Considerations: Acute coronary syndrome, heart failure, arrhythmia, or pneumonia, but these are less likely given the clinical presentation and initial investigations. 2. Initial Approach and Investigations:    - D-Dimer Test: Though the sensitivity of D-dimer for PE is high, in the setting of multiple myeloma and chemotherapy, it may be less specific.    - CT Pulmonary Angiography (CTPA): The definitive investigation for diagnosing PE.    - Echocardiogram: To assess for signs of right ventricular strain and evaluate cardiac function.    - Leg Ultrasound: To look for deep vein thrombosis (DVT), which can be a source of emboli.    - Further Blood Tests: Including troponins, BNP or NT-proBNP, and a complete blood count. 3. Treatment and Further Investigations:    - Anticoagulation: Immediate anticoagulation is crucial in suspected PE. Heparin (unfractionated or low molecular weight) is typically used initially.    - Thrombolytic Therapy: Considered in cases of massive PE with hemodynamic instability. However, it carries a risk of bleeding, especially in a patient on chemotherapy.    - Supplemental Oxygen: To maintain oxygen saturation.    - Fluids and Vasopressors: If necessary, to support blood pressure.    - Definitive Diagnosis: A CTPA or V/Q scan should be performed as soon as possible to confirm the diagnosis of PE.    - Considerations for Further Investigations: The patient's history of multiple myeloma and chemotherapy treatment may complicate the choice and dosing of anticoagulation and thrombolytic therapy. A careful balance between treating the suspected PE and managing the risk of bleeding is essential.   Immediate and aggressive management is warranted in suspected PE, especially in patients with multiple risk factors and signs of hemodynamic compromise. The treatment plan should be tailored to the individual patient's risk profile and underlying conditions. | 1. 5  2. 5  3. 6 | 1. 3  2. 2  3. 3 |

**Case 23** (Reference case 57)

| **Participants** | **Generated answer** | **Accuracy scores** | **Completeness scores** |
| --- | --- | --- | --- |
| **GP** | 1. Acute pancreatitis  2. He needs blood tests(CBC, routine chemistry like ast/alt, total bilirubin, indirect bilirubin, electrolyte, amylase, lipase), abdominal x-ray, abdomen CT, ultrasound of RUQ of abdomen.  3. He needs bowel rest. So NPO and vigorous fluid resuscitation. | 1. 5  2. 4  3. 4 | 1. 2  2. 2  3. 2 |
| **R3** | 1. Acute cholecystitis, Gallstone, possible acute gastritis  2. routine chemistry, eletrolyte, inflammatory marker (esr/crp), abdominal ct  3. If acute cholecystitis is the diagnosis, surgical approach is necessary. antibiotics can be used, pain killer & antiemetics to reduce the symptoms | 1. 4  2. 3  3. 4 | 1. 2  2. 2  3. 2 |
| **R4** | 1. acute cholecystitis  2. abdominal sonography or CT, lab(CBC, CRP)  3. pain control with some NSAIDs, definitively he needs cholecystectomy | 1. 4  2. 5  3. 4 | 1. 2  2. 2  3. 2 |
| **Chat-GPT** | The clinical presentation of this 43-year-old overweight male with diabetes and hypertension suggests a diagnosis of acute pancreatitis.   1. Diagnosis:    - Acute Pancreatitis:      - Supporting Evidence: The key features include upper abdominal pain radiating to the back, vomiting, fever, and tachycardia. The pain characteristic (worsening and longer duration than usual) and location (upper abdomen) are typical for pancreatitis. His lifestyle factors (significant alcohol intake and smoking) are also known risk factors for pancreatitis. 2. Investigations:    - Serum Amylase and Lipase: Elevated levels are indicative of pancreatitis.    - Complete Blood Count (CBC): To check for signs of infection or inflammation.    - Liver Function Tests (LFTs): To assess liver involvement and rule out biliary causes.    - Calcium and Lipid Profile: As hypercalcemia and hyperlipidemia can cause pancreatitis.    - Abdominal Ultrasound: To evaluate the gallbladder (for gallstones) and pancreas.    - CT Scan of the Abdomen: If the diagnosis is uncertain or to assess the severity of pancreatitis.    - Chest X-ray: To check for complications like pleural effusion. 3. Management:    - Fluid Resuscitation: Aggressive intravenous hydration is crucial, especially in the first 24-48 hours, to prevent hypovolemia.    - Pain Management: Adequate analgesia, often with opioids.    - Nutritional Support: Initially, nothing by mouth to rest the pancreas, followed by a low-fat diet. If unable to tolerate oral intake, enteral nutrition may be needed.    - Monitoring: Close monitoring of vital signs, fluid balance, and symptoms. Monitoring in a high-dependency or intensive care unit may be necessary for severe cases.    - Treatment of Underlying Cause: Cessation of alcohol and smoking. If gallstones are identified, management of gallstones may be required.    - Blood Sugar Control: Management of his diabetes, as pancreatitis can exacerbate hyperglycemia.    - Prevention of Complications: Monitoring for and treating complications like infection, pancreatic necrosis, or organ failure.   Early recognition and management of acute pancreatitis are essential to prevent complications and improve outcomes. Lifestyle modifications and addressing the underlying cause are also crucial aspects of long-term management. | 1. 5  2. 5  3. 5 | 1. 2  2. 2  3. 3 |

**Case 24** (Reference case 63)

| **Participants** | **Generated answer** | **Accuracy scores** | **Completeness scores** |
| --- | --- | --- | --- |
| **GP** | 1. Acute appendicitis  2. Abdomen CT  3. Through abdomen CT, we have to see if abscess is formed near the appendix. If there is abscess, we need interval appendectomy. Before appendectomy, we need fluid resuscitation, antibiotics. After the inflammation is resolved, we can operate the surgery. If there isn’t abscess, we can operate emergent appendectomy | 1. 4  2. 3  3. 4 | 1. 2  2. 2  3. 2 |
| **R3** | 1. acute appendicitis, diverticulitis, possible inflammatory bowel disease (e.g. crohn, UC)  2. abdominal ct  3. if acute appendicitis is the diagnosis, surgical approach is necessary, diverticulits & inflammaotry bowel disease require medication (antibiotics) | 1. 5  2. 3  3. 4 | 1. 2  2. 2  3. 2 |
| **R4** | 1. maybe acute appendicitis, or diverticulitis  2. abdominal CT  3. pain control with NSAIDs, definitively he needs appendectomy | 1. 5  2. 3  3. 3 | 1. 2  2. 2  3. 2 |
| **Chat-GPT** | The presentation of this 19-year-old male is suggestive of appendicitis.   1. Diagnosis:    - Appendicitis:      - Supporting Evidence: Lower right-sided abdominal pain, initially cramping and then becoming constant, associated with anorexia, nausea, feverish feeling, and loose stools. Physical examination findings of tenderness in the right iliac fossa and the absence of renal angle pain, abdominal mass, or organomegaly further support this diagnosis. 2. Appropriate Investigations:    - Complete Blood Count (CBC): To assess for leukocytosis, which is common in appendicitis.    - C-Reactive Protein (CRP): Elevated levels can support the diagnosis of an inflammatory process.    - Urinalysis: To rule out urinary tract infection or kidney stones, though a trace of blood can sometimes be seen in appendicitis.    - Abdominal Ultrasound: Particularly useful in younger patients, to visualize the appendix and assess for inflammation.    - CT Scan of the Abdomen and Pelvis: Provides a more detailed view and is helpful if the diagnosis is uncertain or if complications like abscess formation are suspected. 3. Management:    - Surgical Consultation: Appendicitis typically requires surgical intervention, usually an appendectomy.    - Antibiotics: Empirical antibiotics are often started preoperatively and may continue postoperatively, especially if there is evidence of perforation or abscess.    - Fluid Resuscitation: Intravenous fluids to address dehydration and any hemodynamic instability.    - Pain Management: Providing adequate pain relief while being cautious not to mask symptoms that might be important for diagnosis and monitoring.    - Monitoring: Close observation of vital signs, abdominal examination findings, and laboratory values.    - Avoiding Oral Intake: Until surgery, to reduce the risk of aspiration and because surgery is likely.   Timely diagnosis and management of appendicitis are crucial to prevent complications such as perforation, which can lead to peritonitis or abscess formation. | 1. 5  2. 6  3. 5 | 1. 3  2. 2  3. 3 |

**Case 25** (Reference case 71)

| **Participants** | **Generated answer** | **Accuracy scores** | **Completeness scores** |
| --- | --- | --- | --- |
| **GP** | 1. Right-sided otitis media  2. Blood tests like CBC, routine chemistry(crp, esr and so on), electrolyte are needed.  3. Antibiotics like amoxicillin are needed. | 1. 3  2. 2  3. 3 | 1. 2  2. 2  3. 2 |
| **R3** | 1. acute otitis media  2. temporal contrast ct would be beneficial  3. drainage, antibiotics | 1. 3  2. 3  3. 4 | 1. 1  2. 2  3. 2 |
| **R4** | 1. malignant otitis externa  2. lab test, brain/facial CT  3. broad spectrum antibiotics, surgery if needed | 1. 5  2. 4  3. 5 | 1. 2  2. 2  3. 2 |
| **Chat-GPT** | The presentation of this 78-year-old diabetic gentleman suggests a diagnosis of malignant (necrotizing) otitis externa.   1. Diagnosis:    - Malignant Otitis Externa:      - Supporting Evidence: Severe otalgia, purulent otorrhoea, hearing loss, vertigo, and tinnitus in the context of a prolonged history of otitis externa, especially in a diabetic patient. The presence of facial nerve palsy is particularly concerning and indicates the possibility of extension of the infection beyond the external auditory canal, a hallmark of malignant otitis externa. 2. Investigations:    - Blood Tests:      - Complete Blood Count (CBC): To look for signs of infection.      - Erythrocyte Sedimentation Rate (ESR) and C-Reactive Protein (CRP): Elevated levels can indicate severe infection or inflammation.      - Blood Cultures: If there's suspicion of systemic infection.      - Blood Glucose and Hemoglobin A1c: To assess diabetic control, as poor control can exacerbate infections.    - Microbiology:      - Ear Swab Culture: To identify the causative organism, typically Pseudomonas aeruginosa in malignant otitis externa.    - Imaging:      - CT Scan and/or MRI of the Temporal Bone: To assess the extent of infection, particularly bone involvement and potential spread to nearby structures.    - Audiometry: Might be considered to assess the extent of hearing loss. 3. Management:    - Antibiotic Therapy: Prompt initiation of intravenous antibiotics effective against Pseudomonas (e.g., ciprofloxacin) is crucial. Long-term therapy is often required based on culture sensitivity and clinical response.    - Pain Management: Adequate analgesia for severe otalgia.    - Local Care: Cleaning and debridement of the ear canal to remove debris and pus.    - Glycemic Control: Tight control of blood glucose levels, as poor control can impair the immune response and exacerbate the infection.    - ENT and Infectious Disease Consultation: Multi-disciplinary management involving an otolaryngologist and infectious disease specialist is essential.    - Monitoring and Follow-up: Close monitoring of clinical signs and symptoms, inflammatory markers, and imaging studies to assess the response to treatment and identify complications.   Malignant otitis externa is a serious, potentially life-threatening condition requiring aggressive treatment and close follow-up, particularly in diabetic patients and the elderly. The involvement of the facial nerve indicates a more advanced disease that necessitates urgent intervention. | 1. 5  2. 6  3. 6 | 1. 3  2. 2  3. 3 |

**Case 26** (Reference case 73)

| **Participants** | **Generated answer** | **Accuracy scores** | **Completeness scores** |
| --- | --- | --- | --- |
| **GP** | 1. Sinusitis. bad cold of past week  2. Water’s, Caldwell view of x-ray. CBC, routine chemistry, electrolyte  3. Amoxicillin is needed | 1. 3  2. 3  3. 3 | 1. 2  2. 2  3. 2 |
| **R3** | 1. acute rhinitis, presetal orbital cellulitis  2. water's view xray, orbital contrast ct  3. antibiotics | 1. 4  2. 4  3. 4 | 1. 2  2. 2  3. 1 |
| **R4** | 1. orbital cellulitis, due to sinusitis  2. facial CT, lab test  3. antibiotics | 1. 4  2. 3  3. 4 | 1. 2  2. 2  3. 1 |
| **Chat-GPT** | The presentation of this 6-year-old child suggests a diagnosis of preseptal cellulitis.   1. Diagnosis and Cause:    - Preseptal (Periorbital) Cellulitis:      - Supporting Evidence: Right eyelid inflammation and erythema without restriction of eye movements, pain on eye movement, chemosis, or visual acuity compromise. The history of a 'bad cold' with significant nasal discharge suggests a preceding upper respiratory tract infection, which is a common predisposing factor for preseptal cellulitis in children.    - Cause: Likely bacterial infection secondary to the recent upper respiratory tract infection. The green nasal discharge and swollen nasal mucosa indicate a significant nasal infection, which could have spread to the eyelid. 2. Appropriate Investigations:    - Blood Tests:      - Complete Blood Count (CBC): To assess for signs of infection.      - C-Reactive Protein (CRP): As an indicator of inflammation.    - Imaging:      - Orbital CT Scan or MRI: If orbital cellulitis (a more serious condition involving the orbit behind the septum) is suspected based on worsening symptoms or if there are signs of orbital involvement like proptosis, ophthalmoplegia, or impaired vision.    - Microbiology:      - Nasal Swab Culture: To identify the causative organism.      - Blood Cultures: If there's suspicion of systemic infection.    - Eye Swab Culture: If there is any discharge from the eye. 3. Management:    - Antibiotic Therapy: Systemic antibiotics that cover common pathogens (like Staphylococcus aureus and Streptococcus species). Oral antibiotics are often sufficient for preseptal cellulitis unless the patient is systemically unwell or not improving.    - Nasal Decongestants and Saline Sprays: To manage the underlying nasal infection.    - Pain and Fever Management: Paracetamol or ibuprofen can be used for symptomatic relief.    - Close Monitoring: Monitor for signs of worsening infection or progression to orbital cellulitis, which would require more aggressive treatment, possibly including intravenous antibiotics and hospitalization.    - Follow-up: Regular follow-up to ensure resolution of the infection and to reassess if symptoms worsen or do not improve with initial treatment.   Preseptal cellulitis, while less severe than orbital cellulitis, still requires prompt treatment and careful monitoring to prevent complications and ensure complete recovery. | 1. 5  2. 6  3. 5 | 1. 2  2. 2  3. 3 |

**Case 27** (Reference case 81)

| **Participants** | **Generated answer** | **Accuracy scores** | **Completeness scores** |
| --- | --- | --- | --- |
| **GP** | 1. Non-inflammatory diarrhea, and the most likely cause is food poisoning  2. CBC, routine chemistry(crp, esr and so on), electrolyte  3. Especially, restoration of dehydration in children is more important than that in adults. So, fluid(normal saline) resuscitation is needed. And based on the blood test, if the case is inflammatory diarrhea, antibiotics are needed. | 1. 3  2. 3  3. 4 | 1. 2  2. 1  3. 2 |
| **R3** | 1. acute gastroenteritis  2. abdomainal xray, routine chemistry, inflammatory marker, blood sugar, vbga  3. IV hydration, gi medication, admission if necessary | 1. 4  2. 4  3. 4 | 1. 2  2. 2  3. 1 |
| **R4** | 1. acute gastroenteritis, due to some viral infection  2. lab test to find out how severe his dehydration is  3. Continue oral feeding as much as possible, but supplement any deficit with IV fluids. | 1. 4  2. 5  3. 4 | 1. 2  2. 2  3. 2 |
| **Chat-GPT** | The clinical presentation of this 2-year-old male suggests a diagnosis of gastroenteritis.   1. Most Likely Diagnosis and Cause:    - Gastroenteritis:      - Supporting Evidence: The symptoms of diarrhea and vomiting, with reduced urine output and slight dehydration (evidenced by dry mucous membranes), are typical of gastroenteritis. The fact that his father has similar symptoms suggests a likely infectious cause, possibly viral.      - Most Likely Cause: Given the symptom profile and the familial occurrence, a viral etiology, such as rotavirus or norovirus, is most probable. 2. Further Assessment and Investigations:    - Assessment of Hydration Status: A thorough assessment to gauge the degree of dehydration, which includes monitoring urine output, assessing skin turgor, and checking mucous membranes.    - Blood Tests: May not be necessary in uncomplicated cases but can be considered in severe cases, including Complete Blood Count (CBC) and serum electrolytes to check for electrolyte imbalances.    - Stool Sample: To identify the causative organism, especially if there is blood or mucus in the stool or if the patient has a high fever, though this is not always necessary in viral gastroenteritis.    - Weight Measurements: To monitor fluid loss, especially if dehydration is a concern. 3. Key Principles in Treating This Patient:    - Oral Rehydration Therapy (ORT): The mainstay of treatment for mild to moderate dehydration. Oral rehydration solutions (ORS) are preferred to plain water to replace lost electrolytes.    - Dietary Management: Continue regular diet as tolerated. Avoid sugary or highly fatty foods which can worsen diarrhea.    - Monitoring Fluid Intake and Output: Keep track of how much fluid the child is taking in and how much urine is being produced.    - Control of Nausea and Vomiting: If persistent, antiemetic medication can be considered.    - Probiotics: Can be helpful in some cases of viral gastroenteritis.    - Avoidance of Antibiotics: Unless there is strong evidence or suspicion of bacterial infection.    - Education for Caregivers: Advice on symptoms monitoring, ORT administration, and when to return to the hospital (e.g., signs of worsening dehydration, inability to tolerate fluids, bloody stool).    - Follow-up: Ensure follow-up if symptoms do not improve or worsen.   In most cases, gastroenteritis in children is self-limiting, with the focus of treatment being on preventing dehydration and ensuring adequate nutritional intake. Hospital admission is rarely needed unless dehydration is severe or there are other complicating factors. | 1. 6  2. 5  3. 5 | 1. 3  2. 2  3. 3 |

**Case 28** (Reference case 92)

| **Participants** | **Generated answer** | **Accuracy scores** | **Completeness scores** |
| --- | --- | --- | --- |
| **GP** | 1. PID, N.gonorrhea and C.trachomatis  2. To see if there is cervicitis, she needs culture of cervix. As PID is the ascending infection of cervicitis, this will help investigate PID.  3. Antibiotics like ceftriaxone, azithromycin are needed. If the patient has severe symptoms, she needs admission and IV antibiotics treatment. If the patient can tolerate, she can get oral antibiotics and have follow-up in out-patient clinic. | 1. 4  2. 3  3. 4 | 1. 2  2. 2  3. 2 |
| **R3** | 1. pelvic inflammatory disease  2. abdominal contrast ct  3. antibiotics | 1. 4  2. 2  3. 3 | 1. 1  2. 1  3. 1 |
| **R4** | 1. most likely pelvic inflammatory disease, due to IUCD or STD(transmitted by new partner)  2. transvaginal pelvic sonography, abdominal CT, lab test  3. removal of IUCD, giving antibiotics, controlling pain | 1. 5  2. 4  3. 4 | 1. 2  2. 2  3. 2 |
| **Chat-GPT** | The clinical presentation of this 45-year-old woman suggests a diagnosis of pelvic inflammatory disease (PID), potentially related to the recent insertion of a copper intrauterine contraceptive device (IUCD).   1. Most Likely Diagnosis and Cause:    - Pelvic Inflammatory Disease (PID):      - Supporting Evidence: Symptoms of lower abdominal pain, deep dyspareunia, feverish feeling, and foul-smelling green vaginal discharge, along with cervical excitation and bilateral adnexal tenderness on examination, are characteristic of PID. The recent IUCD insertion and new sexual partner are significant risk factors for PID.    - Cause: The infection is likely caused by a sexually transmitted infection (STI), possibly compounded by the recent IUCD insertion, which can sometimes facilitate the ascent of bacteria into the upper genital tract. 2. Investigations:    - Swabs for STIs: Endocervical swabs for gonorrhea and chlamydia PCR testing.    - Vaginal Discharge Culture: To identify causative organisms and guide antibiotic therapy.    - Complete Blood Count (CBC): To look for signs of infection (e.g., elevated white blood cells).    - C-Reactive Protein (CRP): As an inflammatory marker.    - Pelvic Ultrasound: To assess for complications of PID such as tubo-ovarian abscess.    - Urine Pregnancy Test: To exclude pregnancy, as ectopic pregnancy can present with similar symptoms.    - Liver Function Tests (LFTs): If upper abdominal pain is a concern or if hepatitis is suspected. 3. Key Principles in Treating This Patient:    - Antibiotic Therapy: Broad-spectrum antibiotics to cover typical organisms involved in PID, such as Chlamydia trachomatis and Neisseria gonorrhoeae, as well as other common bacteria. Treatment often starts empirically and is adjusted based on culture results.    - Pain Management: Analgesia to manage abdominal pain and discomfort.    - Evaluation of the IUCD: Consideration for removal of the IUCD, particularly if it's thought to be contributing to the infection.    - Fluid Resuscitation and Supportive Care: Especially if the patient is dehydrated or showing signs of systemic infection.    - Education and STI Prevention: Discussing safe sex practices, the importance of STI screening, and partner notification and treatment.    - Follow-up: Close follow-up to ensure resolution of symptoms and to manage any complications.    - Reproductive Health Counseling: Discussing the impact of PID on future fertility and possible complications.   PID requires prompt and effective treatment to prevent complications such as chronic pelvic pain, infertility, and ectopic pregnancy. The recent IUCD insertion and the new sexual relationship are important factors to consider in both the diagnosis and the management of this condition. | 1. 5  2. 6  3. 6 | 1. 3  2. 2  3. 3 |

**Case 29** (Reference case 30 - image-included questions)

| **Participants** | **Generated answer** | **Accuracy scores** | **Completeness scores** |
| --- | --- | --- | --- |
| **R3-1** | 1. atrial fibrillation caused by alcohol or LC induced cardiomyopathy.  2. blood test including cardiac enzyme, pro-BNP, chest and abdomen x-ray, CT to evaluate ascites, echocardiogram  3. Diltiazem or digoxin to treat a.fib , thiamine to treat alcoholism. Consider the ascites and cardiomyopathy. | 1. 6  2. 5  3. 5 | 1. 2  2. 2  3. 2 |
| **R3-2** | 1. SCMP, QRS widening, deep S-wave, ST depression in lateral leads  2. CBC, ammonia, liver function test, electrolyte, cardiac enzyme, pro-BNP, brain CT  3. paracentesis, duphalac enema, symptoms follow up after enema | 1. 4  2. 4  3. 3 | 1. 3  2. 2  3. 2 |
| **R3-3** | 1. Afib RVR, SCMP due to liver cirrhosis  2. echocardiogram, CBC/Blood Chemistry, FAST, Brain CT, liver panel, CXR, blood gas analysis  3. secure airway, inspect any signs of trauma, consider coagulopathy and decreased Intravascular volume due to LC | 1. 4  2. 4  3. 3 | 1. 2  2. 2  3. 2 |
| **R3-4** | 1. a.fib, possible eletrolyte imbalance due to alcoholism  2. LFT, PT/aPTT, T.b/D.b, abdominal ct if necessary  3. paracentesis to reduce ascites, I would consider child-pugh score to evaluate the severity of his LC. | 1. 4  2. 3  3. 2 | 1. 2  2. 1  3. 2 |
| **R4-1** | 1. arterial fibrillation, cardiomyopathy due to alcohol and liver cirrhosis  2. chest x-ray, CBC, coagulation panel, liver function test, cardiac marker, pro-BNP  3. paracentesis, use diuretics | 1. 5  2. 4  3. 4 | 1. 2  2. 2  3. 1 |
| **R4-2** | 1. Aterial fibiliation, triggered by hypoxia d/t pulmoary edema or pleural effusion as a result of alcoholic liver disease and asites  2. echocardiogram, plain chest x-ray, abdomino-pelvic contrast enhanced CT along with chest ct if chest xr reveal abnormality. blood ammonia test, non-enhanced brain ct, diagnostic paracentesis  3. no need to make any rate control if he has no cardio-pulmoary symptom. lactulose enema for r/o HEP / for Afib, we need to consider onset time | 1. 6  2. 5  3. 5 | 1. 2  2. 2  3. 2 |
| **R4-3** | 1. atrial filbibrilation, irregularly irregular rhythm  2. blood test (ammonia, LFT, amylase, lipase, d-dimer, glucose), abdomen ct  3. in acute phase, hydration, analgesisc is needed, when making decisions on treatment, i'll consider his bleeding tendency | 1. 4  2. 3  3. 2 | 1. 1  2. 1  3. 1 |
| **R4-4** | 1. atrial fibrillation, presumably induced by prolonged drinking, dehydration, without eating well  2. lab test(to find out his acid-base status, dehydration, kidney/liver function), echocardiogram, chest x-ray  3. IV or oral hydration is needed to correct dehydration, but heart failure with a-fib is also a possibility, so fluid in/out needs to be well controlled. Due to poor compliance, hospitalization is preferred. | 1. 4  2. 4  3. 3 | 1. 2  2. 2  3. 2 |
| **Chat-GPT** | 1. **ECG Findings**:    - The ECG demonstrates a rapid irregular rhythm without discernible P waves, which is consistent with Atrial Fibrillation (AF). There are also varying QRS complexes which can suggest aberrant conduction or possibly a variable block. There is no clear ST-segment elevation or depression. The T waves are not well-defined in some leads, which may not be specific in the context of an irregular rhythm.   **Precipitation Suspicions**:   - - The patient's history of alcoholism can precipitate atrial fibrillation, commonly referred to as "holiday heart syndrome," especially after bouts of heavy drinking. His background of cardiomyopathy and cirrhosis can also contribute to the development of atrial fibrillation due to altered hemodynamics and electrolyte imbalances.  1. **Investigations in the ED**:    - Comprehensive metabolic panel, including liver function tests, to assess for cirrhosis complications and electrolyte imbalances.    - Complete blood count to identify any concurrent infection or anemia.    - Coagulation studies, given the risk of bleeding or need for anticoagulation.    - Imaging of the abdomen, such as an ultrasound, to evaluate for ascites and assess liver size and texture.    - Echocardiogram to assess cardiac function and structure, particularly the presence of any valvular disease or thrombi.    - Blood alcohol level and toxicology screen, considering the history of alcoholism. 2. **Management**:    - Address airway, breathing, and circulation, provide supplemental oxygen if needed to maintain adequate oxygenation.    - Initial stabilization might include rate control for atrial fibrillation with medications such as beta-blockers or calcium channel blockers, considering his hemodynamic status.    - Thiamine administration to prevent Wernicke’s encephalopathy in the context of alcoholism.    - Management of ascites which may include paracentesis.    - Review the need for anticoagulation to prevent thromboembolic events, which must be balanced against the risk of bleeding, especially in the context of liver disease.    - Nutritional support and vitamins, especially if there is evidence of malnutrition.    - Plan for alcohol withdrawal treatment and arrange for follow-up care, including consideration for rehabilitation services.   This patient's condition is complex due to the multiple chronic issues from alcoholism, and he requires a comprehensive approach to manage both the immediate and long-term complications of his condition. | 1. 6  2. 5  3. 6 | 1. 3  2. 3  3. 3 |

**Case 30** (Reference case 39 - image-included question)

| **Participants** | **Generated answer** | **Accuracy scores** | **Completeness scores** |
| --- | --- | --- | --- |
| **R3-1** | 1. subarachnoid hemorrhage. Intracranial artery aneurysm is the likely underlying cause. Lumbar puncture is not indicated.  2. brain angio ct or angiography, blood test including coagulation test.  3. intubation and mechanical ventilation, sedation, blood pressure control. | 1. 5  2. 5  3. 5 | 1. 2  2. 2  3. 2 |
| **R3-2** | 1. SAH on basal cystern, sylvian fissure. intracranial aneurysm rupture. LP not indicated  2. CBC, coagulation test, ecg, brain CT with angiograpgy  3. NS consult for emergency op or angiography | 1. 5  2. 5  3. 4 | 1. 2  2. 2  3. 2 |
| **R3-3** | 1. SAH, Intracranial aneurysm, hypertension can be an underlying cause, lumbar puncture is not indicated  2. CBC/Blood chemistry, PT/aPTT, CXR for surgical preparation  3. secure airway with definitive airway, administrate tranexamic acid, try to lower BP under 160, refer to NS for surgical intervention | 1. 5  2. 4  3. 4 | 1. 2  2. 2  3. 2 |
| **R3-4** | 1. SAH, aneurysm rupture, lumbar pucture is contraindicated  2. brain angio ct  3. surgical approach | 1. 5  2. 4  3. 4 | 1. 2  2. 2  3. 1 |
| **R4-1** | 1. subarachnoid hemorrhage, due to cerebral aneurysm rupture / lumbar puncture is contraindicated  2. CT angiography  3. Blood pressure control / secure the airway, endotracheal intubation if needed | 1. 4  2. 4  3. 4 | 1. 2  2. 2  3. 2 |
| **R4-2** | 1. high density on Cistern. that means she has a Subarachnoid hemorrhage (SAH), lumbar puncture is not needed  2. CT angiography for find any intracerebral aneurysm  3. Strict blood pressure contol(SBP<140), recurrent Mental check, intubation if the patient has lower GCS score than 8, refer the patient Neurosurgeon immediately | 1. 4  2. 4  3. 5 | 1. 2  2. 2  3. 2 |
| **R4-3** | 1. SAH, may be right pcom aneursym , LP is not indicated on this patient  2. brain angio ct  3. call NS doctor, blood pressure control, emergency surgery | 1. 5  2. 4  3. 3 | 1. 2  2. 1  3. 2 |
| **R4-4** | 1. SAH, probably had a cerebral aneurysm and the rupture caused a hemorrhage. If it's not clear on CT but you suspect SAH, you might try a lumbar puncture, but in this case it's clear and probably not necessary.  2. Evaluation of blood vessels, such as a cerebral angio CT, is needed  3. refer to neurosurgeon to undergo surgery | 1. 5  2. 4  3. 4 | 1. 2  2. 2  3. 2 |
| **Chat-GPT** | 1. **CT Scan Findings and Underlying Cause**:    - The CT scan shows evidence of a hyperdense area within the left hemisphere, suggesting an intracranial hemorrhage. The location and shape could indicate a hemorrhagic stroke, possibly an intracerebral hemorrhage. The mass effect from the hemorrhage appears to be causing a midline shift to the right.    - Given the acute presentation with headache, vomiting, confusion, photophobia, pupillary asymmetry, and gaze palsy, the clinical picture is concerning for increased intracranial pressure due to the hemorrhage.    - A lumbar puncture is contraindicated in this setting due to the risk of brain herniation caused by the introduction of a pressure gradient through the needle insertion. 2. **Other Investigations**:    - An urgent neurological assessment is required.    - Further imaging such as a CT angiogram might be necessary to look for an underlying vascular abnormality, such as an aneurysm or arteriovenous malformation.    - Blood tests including coagulation profile, especially if there is a history of anticoagulant use or if an underlying coagulopathy is suspected.    - Continuous monitoring of vital signs and neurological status, possibly with intracranial pressure monitoring depending on local protocols and available expertise. 3. **Management**:    - The patient should be managed in a high-dependency or intensive care unit with neurosurgical input.    - Management of intracranial pressure is crucial, which may include head elevation, sedation, and possibly osmotherapy (e.g., mannitol or hypertonic saline).    - If an underlying coagulopathy is identified, it must be corrected.    - Seizure prophylaxis might be considered according to local guidelines.    - Surgical intervention may be required to evacuate the hematoma and alleviate the pressure, depending on the size and location of the hemorrhage, the patient's clinical status, and the neurosurgical team's assessment.    - Supportive care including fluid management, fever control, and management of other medical issues is also important.   Immediate consultation with a neurosurgeon is essential for further assessment and management decisions, and the patient should be managed in a setting with neurosurgical facilities. | 1. 3  2. 5  3. 6 | 1. 3  2. 2  3. 2 |

**Case 31** (Reference case 53 - image-included questions)

| **Participants** | **Generated answer** | **Accuracy scores** | **Completeness scores** |
| --- | --- | --- | --- |
| **R3-1** | 1. ACL injury. narrow joint space on lateral meniscus  2. hyperextension injury like skiing  3. cylinder splint apply, ice pack apply, pain control, coultation for op . | 1. 5  2. 4  3. 5 | 1. 2  2. 2  3. 2 |
| **R3-2** | 1. ACL rupture, r/o patella dislocation  2. ski, soccer  3. pain control, immobilization, consider op | 1. 5  2. 4  3. 4 | 1. 2  2. 2  3. 2 |
| **R3-3** | 1. r/o ruptured ACL  2. Patella dislocation or tendon injury  3. Apply cylinder splint, provide analgesics | 1. 5  2. 4  3. 5 | 1. 2  2. 2  3. 2 |
| **R3-4** | 1. ACL rupture, radiograph looks unremarkable  2. ACL injury  3. surgical approach | 1. 5  2. 3  3. 3 | 1. 2  2. 1  3. 1 |
| **R4-1** | 1. Anterior cruciate ligament tear, no fracture in radiograph  2. meniscus tear, hemarthrosis  3. pain control, splint apply, MRI scan, surgery if needed | 1. 5  2. 3  3. 5 | 1. 2  2. 2  3. 2 |
| **R4-2** | 1. ACL ligament injury, X-ray shows upward displacement of patella  2. PCL injury  3. Cylinder splint apply, Knee MR for evaluate ligament injury | 1. 5  2. 2  3. 5 | 1. 2  2. 2  3. 2 |
| **R4-3** | 1. ACL injury, there is no acute fracture on this xray  2. hemarthrosis?  3. refer to os doctor, pain control, surgery if needed | 1. 5  2. 3  3. 4 | 1. 1  2. 1  3. 1 |
| **R4-4** | 1. anterior cruciate ligament injury, suspected concomitant avulsion fracture  2. ankle injury  3. first apply splint and think surgical repair is needed | 1. 4  2. 2  3. 4 | 1. 2  2. 1  3. 1 |
| **Chat-GPT** | 1. **Diagnosis and Radiograph Findings**:    - The diagnosis is likely an Anterior Cruciate Ligament (ACL) injury. This is suggested by the mechanism of injury (fall while skiing), the initial swelling, the feeling of instability particularly on walking up and down stairs, and a positive anterior drawer test.    - The radiographs provided do not show any obvious bone fractures or dislocations in the knee joint, which correlates with the information given to the patient at the sports clinic. The bones appear intact, the joint spaces are preserved, and there are no visible signs of acute osseous injury on these images. 2. **Associated Injuries**:    - With ACL injuries, it's not uncommon to have associated damage to other structures within the knee, such as:      - Meniscus tears, particularly the medial meniscus.      - Injury to the collateral ligaments, although collateral ligament tests were negative in this case.      - Bone bruises or microfractures, which might not be visible on plain radiographs.      - Possible injury to the posterior cruciate ligament (PCL) or other secondary stabilizers of the knee. 3. **Management**:    - Referral to an orthopedic specialist for further evaluation is advised. An MRI of the knee would be beneficial for assessing the soft tissue structures, such as the ACL, PCL, menisci, and cartilage, which are not visible on standard X-rays.    - RICE (Rest, Ice, Compression, Elevation) therapy can help manage swelling and pain.    - NSAIDs (Non-Steroidal Anti-Inflammatory Drugs) can be prescribed for pain relief unless contraindicated.    - Continued use of crutches to avoid weight bearing on the affected leg until the patient can walk without pain or instability.    - Knee brace might be used to provide stability to the knee joint.    - Physiotherapy for strengthening and stability exercises, which is crucial whether or not surgery is indicated.    - If an ACL tear is confirmed, surgical reconstruction might be considered, especially given the patient's young age and activity level. The decision for surgery would be based on the individual patient's goals, activity level, and the degree of knee instability.   The exact management plan would be tailored to the patient after a comprehensive assessment by an orthopedic specialist, including the consideration of an MRI for a definitive diagnosis. | 1. 6  2. 5  3. 6 | 1. 2  2. 3  3. 3 |

**Case 32.** (Reference case 59 - image-included question)

| **Participants** | **Generated answer** | **Accuracy scores** | **Completeness scores** |
| --- | --- | --- | --- |
| **R3-1** | 1. traumatic lung contusion, alveolar hemorrhage, rib fracture  2. chest contrast CT, blood test (cbc, coagulation)  3. oxygenation, pain control. | 1. 4  2. 4  3. 3 | 1. 2  2. 2  3. 1 |
| **R3-2** | 1. rt pneumothorax  2. CBC, coagulation test, inflammatory marker  3. O2 supply, chest tube insertion | 1. 5  2. 4  3. 4 | 1. 2  2. 2  3. 2 |
| **R3-3** | 1. right Pneumothorax, Left rib 5th rib fx  2. Lung ausculation, Chest CT, FAST, cardiac markers, EKG  3. Analgesics, Chest tube insertion for right pneumothorax, supplemental O2 apply | 1. 5  2. 4  3. 5 | 1. 2  2. 2  3. 2 |
| **R3-4** | 1. r/o rib fracture  2. chest ct  3. pain control, observation | 1. 2  2. 3  3. 2 | 1. 1  2. 2  3. 1 |
| **R4-1** | 1. traumatic pneumothorax  2. CBC, routine chemistry, coagulation panel, rib x-ray, chest ct scan if needed  3. tube throacotomy, IV crystalliod, pain control | 1. 3  2. 5  3. 5 | 1. 2  2. 2  3. 2 |
| **R4-2** | 1. Rt pneumotrhoax, Lt. traumatic multiple rib fracture  2. contrast enhanced chest/abdomino-pelvic ct, focused ultrasonography for trauma  3. rt side chest tube/troca insertion for large penumothroax | 1. 5  2. 5  3. 5 | 1. 2  2. 2  3. 2 |
| **R4-3** | 1. traumatic pneumthorax combined rib fracture  2. chest xray, chest ct if needed  3. chest tube insertion, iv analgesics | 1. 4  2. 3  3. 4 | 1. 2  2. 1  3. 2 |
| **R4-4** | 1. rib fracture with hemothorax  2. contrast chest ct, maybe contrast abdominal ct (to assess for accompanying damage)  3. proper oxygen therapy, chest tube insertion, maybe surgery for bleeding control or severe fracture with displacement | 1. 2  2. 4  3. 3 | 1. 1  2. 2  3. 2 |
| **Chat-GPT** | 1. **Diagnosis**:    - The patient likely has a Rib Fracture, suggested by the history of trauma, marked bruising, tenderness over the ribs, and decreased chest expansion and breath sounds on the left side. The unilateral nature of the symptoms and the mechanism of injury support this diagnosis. Furthermore, the decreased oxygen saturation and increased respiratory rate indicate compromised respiratory function, which can occur with rib fractures.    - There is also concern for a possible underlying Pulmonary Contusion or Pneumothorax, which can result from blunt chest trauma and lead to acute shortness of breath. 2. **Investigations**:    - Detailed assessment with a Chest X-ray to evaluate for rib fractures, pneumothorax, pulmonary contusion, or other chest injuries.    - Arterial blood gas (ABG) analysis to assess oxygenation and ventilation status.    - Complete blood count (CBC) and other relevant blood tests to evaluate the overall health status and rule out infection.    - If pneumothorax or hemothorax is suspected, a CT scan may provide more detail. 3. **Management**:    - Pain Management: Adequate analgesia is crucial to allow for proper breathing and coughing, which can prevent complications like atelectasis or pneumonia. NSAIDs or acetaminophen, possibly supplemented with opioids or a nerve block, may be considered.    - Respiratory Support: Supplemental oxygen to maintain SpO2 above 94%. In more severe cases, such as a large pneumothorax, more aggressive interventions such as chest tube placement may be required.    - Monitoring: Close observation for signs of respiratory distress or the development of complications like a tension pneumothorax.    - Breathing Exercises: Encourage deep breathing and use of incentive spirometry to prevent atelectasis.    - Follow-up: Arrange follow-up for re-assessment, as complications can develop even after initial presentation.    - Lifestyle Changes: Addressing his heavy smoking and alcohol use through appropriate referrals for support and counseling.   Given the complexity of potential chest trauma, it's important to consider a broad differential and manage accordingly, with a low threshold for further imaging and specialist input if the patient's condition does not improve or deteriorates. | 1. 6  2. 5  3. 5 | 1. 3  2. 2  3. 3 |
